# Male Circumcision for HIV Prevention in High HIV Prevalence Settings: What Can Mathematical Modelling Contribute to Informed Decision Making?

**DOI:** 10.1371/journal.pmed.1000109

**Published:** 2009-09-08

**Authors:** UNAIDS/WHO/SACEMA Expert Group on Modelling the Impact and Cost of Male Circumcision for HIV Prevention

## Abstract

Experts from UNAIDS, WHO, and the South African Centre for Epidemiological Modelling report their review of mathematical models estimating the impact of male circumcision on HIV incidence in high HIV prevalence settings

**Summary Points**

- Mathematical models can estimate the population-level impact of male circumcision on HIV incidence in high HIV prevalence settings, but different methods, assumptions, and input variables can produce conflicting results.
- UNAIDS/WHO/SACEMA recently convened experts to review the outcomes of six simulation models on key policy and programmatic decision-making questions.
- Large benefits of male circumcision among heterosexual men in low male circumcision, high HIV prevalence settings were found: one HIV infection being averted for every five to 15 male circumcisions performed, and costs to avert one HIV infection ranging from US$150 to US$900 using a 10-y time horizon.
- The models predicted that both premature postoperative resumption of sexual intercourse and behavioural risk compensation, if confined to newly or already circumcised men and their partners, have only small population level effects on the anticipated impact of male circumcision service scale-up on HIV incidence.
- Women benefit indirectly from reduced HIV prevalence in circumcised male partners and male circumcision service scale-up acts synergistically with other strategies to reduce HIV disease burden.
- The modelling results have informed development of a pragmatic decision-makers' programme planning tool.

## Background

Three recent randomised controlled trials [1]–[3] in Kenya, South Africa, and Uganda have confirmed previous observational studies [4] and ecological experience [5] and demonstrated beyond reasonable doubt that male circumcision performed by well-trained medical professionals reduces the risk of men acquiring HIV through female-to-male transmission by approximately 60% [5],[6]. Furthermore, results from the Kenyan trial indicate that the protective effects of circumcision are sustained for at least 42 mo [7], which suggests that circumcision is likely to provide life-long partial protection.

Although the evidence from the randomised trials is compelling, the longer-term population-level impact of introducing or expanding safe male circumcision services within comprehensive HIV prevention programmes remains unknown. Consequently, although some countries with a high prevalence of HIV have held stakeholder meetings and are developing policies on male circumcision for HIV prevention, many have not done so. In addition, the introduction and/or expansion of male circumcision programmes for HIV prevention raises a host of ethical, legal, and human rights issues [8]–[10]. Furthermore, the introduction/expansion of these programmes could be hindered by weak health infrastructures, scarce human resources for health [11], cultural concerns, political barriers, and financial constraints. In the face of these challenges, some decision-makers in sub-Saharan Africa are asking whether the introduction or expansion of male circumcision services for the reduction of HIV incidence will be cost-effective over the short, medium, and long term.

Estimating the long-term population impact and cost-effectiveness of male circumcision programmes requires mathematical modelling approaches. However, when different modelling approaches use different baseline assumptions and input variables, they sometimes produce conflicting results. The Joint United Nations Programme on HIV/AIDS (UNAIDS), the World Health Organization (WHO), and the South African Centre for Epidemiological Modelling and Analysis (SACEMA) recently convened three expert group meetings in Geneva (2005), Stellenbosch (2007), and London (2008) to review published and unpublished modelling work. Specifically, the expert group meetings assessed the potential population-level effects of male circumcision on HIV incidence predicted by these models and determined the relevance of mathematical modelling approaches to informed decision-making about the scale-up of male circumcision programmes.

## The Mathematical Models

At the 2008 meeting, the expert group reviewed the following mathematical models for the effects of male circumcision on HIV incidence and prevalence:

- A deterministic compartmental model based on scenarios for settings similar to Botswana and Nyanza Province, Kenya [12];
- A stochastic simulation model that included parameters empirically derived from a cohort in Rakai, Uganda [13];
- A very simple compartmental model using South African data to estimate epidemiological parameters and to construct an aggregate model for sub-Saharan Africa [14];
- Two deterministic compartmental models of heterosexual HIV spread in populations stratified for sex and risk behaviour [15],[16];
- An individual-based micro-simulation model with formation and dissolution of heterosexual relationships and HIV transmission modelled as stochastic events [17].

To estimate the costs associated with changes in HIV prevalence and incidence predicted by each model, the expert group used data from a cost-effectiveness study based on the randomised controlled South African trial [18],[19], costing information from the randomised controlled Kenyan [20] and Ugandan trials [13], and data from costing studies conducted in Lesotho [21], Swaziland [22], and Zambia [23].

Summary properties of these models (and two additional modelling exercises published after the third meeting [24],[25]), are shown in Table 1, together with key results.

**Table 1:** Comparison of models and results from HIV dynamic transmission models used to evaluate the impact of male circumcision.

Citation	Model Type	Setting (HIV Prevalence)	Stratification/Heterogeneities in Model Population	Published Model Analysis Incorporates:						Range of Estimates, Assuming Universal/Near Universal Circumcision Coverage^a^			
				Effect of ART/Other Interventions	Effect of STI Cofactors	Reduction in Male-to-Female Transmission	Circumcision of Infected Men	Risk Compensation among Circumcised Men	Risk Compensation in Population Overall	Percentage Reduction in Incidence (per Person-Year at Risk), 10 y after Intervention Starts/at Equilibrium	Percentage Reduction in HIV Prevalence Due to Intervention, 10 y after Intervention Starts/at Equilibrium	Number of Operations Required per Infections Averted, over 10 and 20 y	HIV Epidemic Aborted by Optimistic Circumcision Intervention Alone
**Williams et al. ** [**14**]	Closed-solution analysis and deterministic simulation	Africa and South Africa (22%)	Homogeneous	No	No	No	No	No	No	37% (eq)	24%	Not quoted	No
**Nagelkerke et al. ** [**12**]	Closed-solution analysis and deterministic simulation	Botswana (35%) and Nyanza, Kenya (20%)	Sexual risk behaviour	No	No	No	No	No	No	55% (eq)	17%–20% (eq)	Not quoted	No
**Gray et al. ** [**13**]	Stochastic simulation	Rural Uganda (11%)	Sex, age, sexual-risk behaviours	No	No	Yes	Yes	Yes	No	10%^b^^,^ c	Not quoted	39 (10 y)^c^	No^b^
**Hallett et al. ** [**15**]	Deterministic simulation	Southern/Eastern Africa (23%)	Sex, sexual-risk behaviour	Yes	No	Yes	Yes	Yes	No	40% (10 y)	23%	15 (10 y)	No
												10 (20 y)	
**White et al. ** [**17**]	Stochastic network simulation	Kisumu, Kenya (25%)	Sex, sexual-risk behaviour, age	Yes	Yes	Yes	Yes	Yes	Yes	38% (10 y)	23%	8 (10 y)	No
												3 (20 y)	
**Alsallaq et al. ** [**16**]	Closed-solution analysis and deterministic simulation	Kisumu, Kenya (25%) and Africa	Sex, sexual-risk behaviour	No	No	Yes	No	Yes	Yes	29%	19%	6 (10 y)	No
												5 (20 y)	
**Podder et al. ** [**25**]	Deterministic simulation	Not specific	Sex	Yes	No	No	No	No	No	Not quoted	Not quoted	Not quoted	No
**Londish and Murray ** [**24**]	Deterministic simulation	Sub-Saharan Africa	Sex, sexual-risk behaviour, age	No	No	Yes	No	Yes	No	Not quoted	50% (13 y)	Not quoted	No

**Figure 1:**
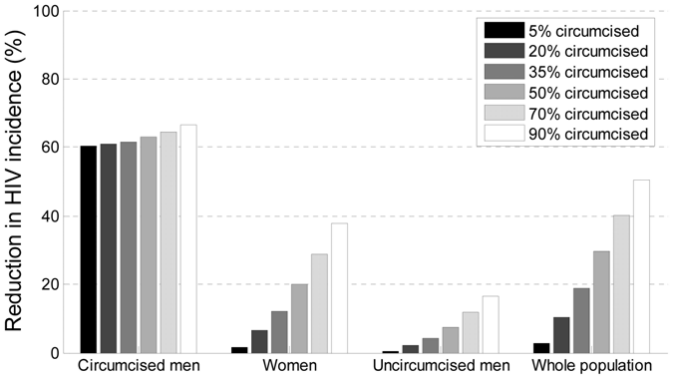
Reductions in HIV incidence by coverage level. This figure shows model estimates for the reduction in HIV incidence 10 y after the programme begins, among circumcised men, women, uncircumcised men, and the population overall, at varying levels of circumcision uptake (from a baseline of 0%). The model [15] is a deterministic compartmental simulation of the heterosexual spread of HIV in a sex- and sexual-activity stratified population, parameterised for Southern and Eastern African populations. The model assumes that there is a 60% reduction in female-to-male transmission for circumcised men, that there is no direct reduction in male-to-female transmission from circumcised men, and that 5%, 20%, 35%, 50%, 70%, and 90% of men are circumcised within 10 y of the intervention being scaled-up. Note: Since the fraction of men circumcised increases over time, the weighted-average of reductions in incidence in these demographic groups at year 10 is not expected to equal the reduction in incidence in the whole population over the first 10 y of the intervention.

The first set of columns show the differences in model structure and the issues investigated in published analyses; the second set of columns show the results from each model making a standard set of assumptions.

a Model simulations with universal or near universal uptake of male circumcision were compared to maximise comparability of outputs since high coverage levels would tend to exaggerate differences in model results. For White et al. [17], circumcision is scaled up from 25% to 75%. Simulated coverage reaches scale within 5 to 10 y for all models. The impact on HIV infections averted with more realistic coverage levels is shown in Figure 1. Where possible, the following standard sets of assumptions are used: 60% reduction in female-to-male transmission, 0% reduction in male-to-female transmission associated with HIV, and no risk compensation.

b Basic reproductive number approximately equal to one, indicating that the epidemic would be on the verge of terminal decline and incidence declines gradually.

c This estimate is based on a simplified model for low risk populations. In the revised and expanded model [16], the estimate for the number of operations per infection averted over the first 10 y of the intervention in a population representative of Rakai, Uganda is 11.

eq, equilibrium.

The six models considered by the expert group had been independently applied to various settings to estimate the overall impact on HIV incidence of the scale-up of male circumcision. The models had also been used to estimate the relative impact of the scale-up of male circumcision among different population subgroups, on the numbers needed to treat, and on cost-effectiveness. Finally, the models had been used to investigate the influence of factors such as declining HIV incidence, potential changes in risk behaviour, and the effects of other HIV prevention programmes. Because observational data on HIV risk and circumcision status among men who have sex with men do not suggest a strong protective effect [26], this population was not included in any of the models. With one recently submitted exception [16], the models had all been published in peer-reviewed scientific journals with detailed supplementary material before being considered by the expert group.

As shown in Table 2, although the models used different methods, baseline assumptions, and input variables, their essential components were similar. For example, the models' programmatic, biological, and behavioural variables generally included age group targets, risk group targets, speed of service scale-up, final level of male circumcision coverage reached, presumed risk of female-to-male and male-to-female HIV transmission, HIV acquisition and transmission risk during postoperative wound healing, and potential risk compensation [27], such as less frequent condom use and increased numbers of sex partners. Modelled outcomes included impact on HIV incidence and HIV prevalence, and the number of male circumcisions required to avert one HIV infection. A brief summary of the models and the analyses conducted with each one is provided in Text S1, and the presentations made at the last two expert group meetings are provided in Text S2.

**Table 2:** Range of variables in the different models.

Author	All Models	Williams et al. [14]	Nagelkerke et al. [12]	Gray et al. [13]	Hallett et al. [15]	White et al. [17]	Alsallaq et al. [16]
**Baseline HIV incidence (per 100 person-years)**	1.2–4.5	2.4	2.2–4.5	1.2	3.2	1.3–3.2	3.9
**Baseline HIV prevalence (%)**	11–36	20	18–36	11	23	11–25	28.8
**Baseline male circumcision prevalence (%)**	0–50	35	10	16	0	0–50	27.5
**Reduction of female-to-male transmission due to circumcision (%)**	30–76	60	40–75	40–70	60	30–76	60
**Target age groups**	5-y age groups	No age structure	No age structure	15–49	5-y age groups^a^	5-y age groups	No age structure
**Target risk groups**	High- versus low-risk behaviour men	All men	All HIV-negative men	All men	High- versus low-risk behaviour men^a^	All HIV-negative men and all men	High- versus low-risk behaviour men
**Time to reach intended coverage (y)**	0–20	5 or 10	Approximately 10	0–10	5	0–20	0–10
**Final circumcision prevalence reached (%)**	25–100	100	50–80	25–100	90	50–100	100
**Reduction of male-to-female transmission risk (%)**	0–70	0	0–25	40–70	0–30	0–50	0
**Proportion of men who resume sex before wound healing (%)**	0–60	N/A	N/A	N/A	0–40	15–60	N/A
**Relative risk of acquiring or transmitting HIV during wound healing**	Up to 2.3	N/A	N/A	N/A	0–2	2.3^b^	N/A
**Increase in sexual partner numbers postcircumcision (%)**	0–200	0	0	0–100	0	0	0–200
**Reduction in condom use (%)**	0–100	N/A	0–100	N/A	0–100 in casual partnerships	0–100 in casual and sex worker contacts	N/A

a These analyses published in [43].

b >6 mo.

N/A, not applicable.

## Application of the Models to Key Questions

Before its third meeting, the expert group identified eight key questions with implications for policy and programmatic decision-making. They then considered the findings from the models relevant to each of the questions in turn at the meeting. Not all of the models addressed all of the topics. Furthermore, in many cases, the quantitative outputs from the models could not be directly compared because alternative underlying assumptions had been made and their results related to different contexts. For this reason, the expert group did not attempt to quantify the variation in model results formally even though each of the published articles contained an analysis of the uncertainty in the relevant model's projections.

During the discussion of each preselected topic, a broad qualitative consensus emerged from the findings of the specific models being examined, which was agreed upon by all the members of the expert group. Indeed, the varied nature of the models and the independence of the researchers involved in these modelling exercises provide support for the generalisability of the findings of the expert group.

## What Is the Expected Impact on HIV Incidence of Scaling Up Male Circumcision Programmes?

The expected impact of scaling up male circumcision services depends on several critical factors including baseline male circumcision and HIV prevalence; whether HIV incidence is increasing, stable, or declining; the time period of model projections; and the speed of scale-up.

WHO/UNAIDS guidance on programme implications [6] states that the greatest potential public-health impact will be in settings where HIV is hyperendemic (HIV prevalence in the general population exceeds 15%) and is spread predominantly through heterosexual transmission, and where a substantial proportion of men (e.g., greater than 80%) are not circumcised. The six models, therefore, focused on settings that have an epidemic profile similar to this.

The models predicted that, using a 10-y time horizon, one new HIV infection would be averted for every five to 15 men newly circumcised. For the most successful interventions, where almost all men are circumcised, HIV incidence could be reduced by ∼30%–50% over the same period, with prevalence following this decrease with some delay (Figure 1; Table 1). Inevitably, the absolute number of male circumcisions required to avert one HIV infection increases as HIV incidence declines over time.

In countries with lower levels of HIV prevalence and incidence, such as Uganda, the number of male circumcisions required to avert one new infection is higher (Table 1) [13]. However, on the basis of its analysis of the model predictions, the expert group agreed that even in such countries, programmes that focus on subpopulations with a high HIV prevalence and incidence would have substantial impact on HIV incidence. Programmes that fall into this category might include, for example, those that focus on geographic areas of low male circumcision prevalence or on subgroups of heterosexual men at higher risk of HIV exposure. These subgroups include HIV-negative men in serodiscordant couples and men more likely to have multiple sex partners, such as soldiers, truck drivers, miners, labour migrants, or patients attending sexually transmitted disease (STD) clinics. The expert group noted that, according to a systematic review and meta-analysis, men at higher risk of STD benefit from higher levels of protection when circumcised (adjusted risk ratio [RR] = 0.29, 95% confidence interval [CI] 0.20–0.41) [4].

## What Is the Overall Impact on HIV Incidence in Women?

As sexual partners and parents, women are affected by male circumcision [28],[29]. Although an observational study suggested that circumcision of HIV-positive men might reduce transmission to HIV-negative female partners [30], no such direct effect was observed in a trial that was prematurely closed for futility [31]. However, among those couples who resumed sexual activity soon after circumcision a nonstatistically significant but nonetheless concerning trend was found in this trial toward an increased risk of HIV infection in women assessed 6 mo after their partners' circumcision.

All six models showed that women, even if not directly protected, would benefit indirectly from the introduction or expansion of male circumcision services because their probability of encountering an HIV-infected male sexual partner gradually declines with programme scale-up. In the models, these indirect benefits increase over time, taking some years to become evident (Figure 1). The expert group noted that these indirect benefits would eventually reduce the number of women needing services to prevent mother-to-child HIV transmission, although the proportion of people living with HIV who are women would increase [14].

In addition, the expert group reviewed empirical data that show that male circumcision reduces the acquisition of herpes simplex virus type-2 [32], syphilis, and chancroid in HIV-negative men [3],[33], and accumulating evidence that the circumcision of HIV-positive men provides direct benefit to women by reducing genital ulcer disease [34], which may decrease the likelihood of HIV transmission. In the Ugandan trial, women who were the sexual partners of circumcised HIV-negative men had less genital ulcer disease and bacterial vaginosis, and fewer *Trichomonas vaginalis* infections than women with uncircumcised male partners [35]. Although all these conditions, with the possible exception of bacterial vaginosis [36], are associated with an increased risk of female HIV acquisition, only one of the models analysed by the expert group explicitly included this mechanism [17], which was also not fully represented in another recent study [37].

Overall, the expert group concluded that any of these mechanisms for the reduction in HIV acquisition for women could enhance the overall impact of male circumcision and could hasten reductions of HIV incidence among women.

## What Is the Impact of Circumcising HIV-Positive Men?

WHO/UNAIDS advise against promoting male circumcision for HIV-positive men, but state that it should not be denied unless medically contraindicated [6]. HIV testing is recommended for all men seeking male circumcision, but is not mandatory [6]. The systematic refusal to circumcise HIV-positive men based on their HIV status alone may increase stigma for all uncircumcised men.

The expert group found that one model predicted that circumcision of HIV-positive men in the context of capacity constraints would mean that fewer HIV-negative men would be circumcised, thus reducing the population-level impact of a circumcision programme on HIV incidence over the short term [17]. However, the model also suggested that under some circumstances, this negative result may be partially offset by lower rates of genital ulcer disease and reduced onward HIV transmission to female partners [17].

Two models showed that, assuming no direct effect of circumcision on male-to-female HIV transmission, premature postoperative resumption of sex by HIV-positive men is unlikely to have an adverse population-level effect on overall HIV incidence because any increased risk only applies for a short period of time, a matter of weeks [15],[17]. When the proportion of HIV-positive men resuming sex early was set at 60%, one model showed that the population-level effect on anticipated HIV incidence remains small [17]. It should be noted that in the trials, only 4%–20% of HIV-negative men resumed sex early [1]–[3]. Nonetheless, the expert group concluded that, regardless of serostatus, it is important to counsel newly circumcised men and their partners on the potential for disruption of wound edges if sex is resumed too soon after surgery.

The proportion of male circumcisions performed on HIV-positive men will depend on HIV prevalence and on how many clinically eligible HIV-positive men request surgery. This number is influenced by scale-up strategies (e.g., there is a higher HIV prevalence in STD clinics), the perception of circumcision in the community (e.g., whether it is perceived as a marker of HIV-negative status or as a marker of higher risk), and messages given to men testing positive or declining HIV testing. Notably, one of the models indicates that good uptake among men with the highest risk of HIV exposure could amplify the impact of circumcision programmes [15], even though focusing programmes on such subgroups will likely lead to more men who are already infected being circumcised.

## What Is the Effect of Risk Compensation?

As observed with antiretroviral treatment, a decrease in perceived risk can result in an increase in sexual risk-taking behaviour, a phenomenon termed “risk compensation” [27],[38],[39]. The randomised trials of male circumcision [1],[3],[40] and an observational study [41] found minimal or no behavioural risk compensation among recently circumcised men, although intensive health education during the trials might have mitigated risk compensation.

The models showed that if risk compensation is confined to newly or already circumcised men and their partners, it has only a small effect on the projected population-level impact of male circumcision on HIV incidence [15]–[17]. At high levels of risk compensation (e.g., no male or female condom use), women who partner with circumcised men believing them to be HIV-negative may be placed at increased individual risk despite lower HIV incidence in the whole population [15].

Furthermore, the models suggested that the beneficial impact of male circumcision for both men and women would be substantially reduced if risk behaviours increase across the entire adult population, including among uncircumcised men and their partners. In light of these findings, the expert group agreed that there is a clear need for intensive social change communication campaigns, aimed at the whole population, to prevent increases in risk behaviours.

## How Do the Effects Vary by Age Group Circumcised?

It is clear that a scale-up of male circumcision that prioritises the treatment of subgroups of heterosexual men at the highest risk of HIV exposure will have the most rapid initial impact. These subgroups vary by country but include seronegative men in discordant couples identified during couple counselling and testing, STD clinic attendees, and adult males 15–34 y old. In many settings HIV incidence is highest among 25- to 34-y-old men [42] rather than 15- to 24-y-olds. Because changes may occur over time, HIV incidence monitoring in relevant subpopulations is essential to ensure that priority groups continue to be accurately identified.

The models indicated that circumcising men who have not started sexual activity leads to the greatest population-level benefit in the long term, whereas circumcising 25- to 34-y olds has the biggest benefit over the following 10 to 20 y; circumcising 50-y-old men has little effect on HIV incidence [17],[43]. In the context of parents and guardians deciding to circumcise their sons [9] as neonates rather than when they are older, since the procedure is simpler, cheaper, and incurs fewer adverse events, the models show that reductions in population incidence would probably take 20 to 25 y to become evident, other factors being equal. Of course, circumcising both adult males and neonates would maximise the short- and long-term impact of circumcision on HIV incidence. But, if a fully effective HIV vaccine for adults is widely accessible by 2025 or high levels of treatment uptake are achieved [44], some neonatal circumcision performed now solely for the purpose of HIV prevention would have been unnecessary and thus the projections of cost-effectiveness of this strategy would be exaggerated.

## How Do the Effects Vary with Speed of Service Scale-up?

All the models showed that rapid expansion of male circumcision coverage will result in earlier and larger effects on HIV incidence (Figure 1), assuming that safety standards and the quality of counselling and postoperative care are maintained. The models showed that whether scale-up rates are constant, faster initially then slowing, or slower initially with subsequent acceleration, they can still achieve a specified goal by the target date. However, studies in Lesotho, Swaziland, and Zambia found that a faster initial scale-up would avert between 13.7% and 16.1% more infections by 2015 compared to a linear scale-up, whereas a slower initial scale-up would result in −19.7% to −14.5% fewer infections averted, assuming a target coverage in each country of around 50% by 2015 [45]. Thus, the expert group concluded from both the models and empirical data that rapid initial scale-up accrues direct and indirect effects earlier and is considerably more cost-effective, with fewer circumcisions required to avert one infection and more HIV infections averted at lower cost per infection averted over time.

## How Does Scale-up of Other Prevention Initiatives at the Same Time Affect the Impact of Male Circumcision Scale-up?

The introduction or expansion of male circumcision services will occur in settings where behavioural prevention programmes (e.g., campaigns to increase male and female condom use or to reduce numbers of sexual partners) and biomedical measures (e.g., antiretroviral treatment) may be reducing sexual HIV transmission. Unlike other HIV prevention strategies that depend on user-adherence, male circumcision, once performed, is likely to provide lifelong partial protection against HIV, on the basis of the available evidence. Furthermore, the scale-up of male circumcision to reduce HIV incidence provides an opportunity to enhance other prevention strategies such as counselling to reduce risky behaviours, to increase correct and consistent male and female condom use, and to encourage knowledge of HIV serostatus.

All the models showed that male circumcision would not, in isolation, have sufficient impact to stop the HIV epidemic (Table 1). However, one model showed that substantial synergies are likely to be achieved by combining approaches [15] with the greatest impact generated when circumcision is scaled up in parallel with an intensified focus on reducing sexual risk behaviour. Importantly, the expert group agreed that increasing prevention choices for people while treatment access is expanding could potentially speed the decline of the epidemic.

## What Are the Discounted Savings?

The estimated costs per adult male circumcision are between $30 and $60 [45] depending on the programme setting, with neonatal circumcision costing about one-third this amount. The models estimate costs per infection averted of between $150 and $900 in high HIV prevalence settings over a 10-y time horizon, and $100 to $400 when including infections averted to 20 y. All the models indirectly confirmed that the most favourable cost-effectiveness ratios will be seen where HIV incidence is highest. By comparison, estimates of discounted lifetime treatment costs typically exceed $7,000 per HIV infection if only first-line treatment is provided, and twice as much if second-line treatment is available [46]. This estimate assumes first line antiretroviral treatment costs of $300 per patient per year rising to $500 by 2015, laboratory and service delivery costs of $300 per patient per year, survival of 85% in the first year after treatment initiation and 95% in subsequent years, and 3% discount rate. Thus, circumcising sexually active males of any age is likely to be cost saving [17],[18].

## From Models to Decision Making

To assist countries in scaling up safe, voluntary male circumcision services, WHO, UNAIDS, and partners have produced extensive guidance and several useful tools. For example, they have produced human rights guidance, a situational analysis toolkit, a communications framework, a surgical manual and training modules, a legal and regulatory self-assessment tool, and a monitoring and evaluation tool.

Importantly, the Futures Institute in collaboration with UNAIDS and the Health Policy Initiative has produced a pragmatic, decision-makers' programme planning tool [47] that helps analysts and decision-makers understand the costs and impacts of policy options. This tool calculates the cost of male circumcision services by delivery mode on the basis of clinical guidelines and locally derived inputs for staff time and salaries, supplies, equipment, and shared facility and staff costs. It allows decision-makers to make programmatic choices of the intended target population by age (newborn, adolescent, adult) and risk, while varying service delivery modes and ancillary services, scale-up rates, and coverage goals. The tool estimates HIV incidence, HIV prevalence, AIDS deaths, overall costs, and net cost per HIV infection averted as a function of numbers of male circumcisions performed for each service delivery and coverage timeframe option.

The expert group confirmed that this simple, user-friendly tool produces results consistent with the academic mathematical models that they considered. Because academic models cannot be parameterized for every setting and cannot explore every possible type of intervention, the published results from modelling studies cannot be directly relevant to all settings. However, by using the models to refine and validate a user-friendly tool that can be deployed locally, decision-makers can indirectly access the main findings from academic modelling studies.

Why was it important to use modelling studies to refine a tool to be used by decision-makers to design tailored local and national programmes for the scale-up of male circumcision services? Rapid scale-up of male circumcision services in high HIV prevalence settings in sub-Saharan Africa will require substantial funding and many skilled personnel in the short term [19]. It is critical that unintended consequences of scale-up be monitored and rectified; that additional funding is provided; that the new services strengthen existing surgical, sexual, and reproductive health programmes; and that this biomedical addition to combination HIV prevention acts synergistically with other strategies to stimulate and maintain HIV incidence declines. Thus, mathematical modelling by itself is important because it shows the potential for substantial reductions in HIV transmission through the introduction or expansion of male circumcision services. But more importantly, the decision-makers' programme planning tool, which is informed by the modelling consensus presented here, shows what programmes for the scale-up of male circumcision can achieve in specific settings, over what time frame, and at what cost. Finally, provided it is updated with coverage and cost figures as programmes scale up, the tool can also be used to monitor progress and optimize strategies.

## Supporting Information

Alternative Language Summary S1 **Arabic translation of the abstract by Abdel-Hamid Saleh-Hamdan.**
(0.03 MB DOC)

Alternative Language Summary S2 **Chinese translation of the abstract by Yuhua Fan.**
(0.03 MB DOC)

Alternative Language Summary S3 **Danish translation of the abstract by Nicolai Lohse.**
(0.04 MB DOC)

Alternative Language Summary S4 **French translation of the abstract by Jacqueline Rossel.**
(0.03 MB DOC)

Alternative Language Summary S5 **Russian translation of the abstract by Elena Sannikova.**
(0.03 MB DOC)

Alternative Language Summary S6 **Spanish translation of the abstract by Roger Biosca.**
(0.03 MB DOC)

Text S1 **Details of mathematical models presented to the expert group.** Full details and technical specifications of the models presented have been published in the peer-reviewed literature and the presentations made to the Expert Group are provided in Text S2. This document provides a brief unified summary of the setting and the model structure, including key assumptions, along with the analyses performed using the models.
(0.63 MB PDF)

Text S2 **Presentations from meetings held in Stellenbosch, November 2007 and London, March 2008.**
(4.28 MB PDF)
