# Correction: Male Circumcision for HIV Prevention in High HIV Prevalence Settings: What Can Mathematical Modelling Contribute to Informed Decision Making?

**DOI:** 10.1371/annotation/f1c3679d-18d8-4fbd-bd3f-59db0b284b19

**Published:** 2009-12-17

**Authors:** UNAIDS/WHO/SACEMA Expert Group on Modelling the Impact and Cost of Male Circumcision for HIV Prevention

There was a spelling error in the name of a member of the UNAIDS/WHO/SACEMA Expert Group on Modelling the Impact and Cost of Male Circumcision for HIV Prevention. The correct name is Laith Abu-Raddad.

