# Supplementary material for: Male Circumcision for HIV Prevention in High HIV Prevalence Settings: What Can Mathematical Modelling Contribute to Informed Decision Making?
Source: PLoS Med. 2009 Sep 8;6(9):e1000109. doi: 10.1371/journal.pmed.1000109 (PMC2731851; doi:10.1371/journal.pmed.1000109)
Supplement: Alternative Language Summary S1 — Arabic translation of the abstract by Abdel-Hamid Saleh-Hamdan. (0.03 MB DOC) [file pmed.1000109.s001.doc]

- إن النماذج الرياضية يمكنها تقدير الأثر المحتمل لختان الذكورعلى مدى حدوث الإصابات بفيروس نقص المناعة البشري على المستوى السكاني في المناطق ذات مستوى الانتشار المرتفع، إلا أن الاختلاف في الأساليب والافتراضات الأساسية ومتغيرات المدخلات يمكن أن تسفر عن نتائج متضاربة لصانعي القرار.
- ولمساعدة صانعي القرار، قام برنامج الأمم المتحدة المشترك المعني بالإيدز ومنظمة الصحة العالمية ومركز جنوب إفريقيا للنمذجة والتحليل الوبائي (SACEMA) مؤخراً بعقد اجتماع للخبراء بهدف مراجعة ومقارنة النتائج التي قدمتها ستة نماذج محاكاه على ثمانية أسئلة رئيسية لاتخاذ القرارت الخاصة بالسياسات والبرامج.
- وقد نتج عن النماذج نتائج مماثلة، وهي أن فوائد ختان الذكور بين الرجال من ذوي التوجه الجنسي الغيري تكون كبيرة في المناطق التي ينخفض فيها ختان الذكور وينتشر فيها فيروس نقص المناعة البشري، حيث يتم تفادى حالة إصابة واحدة بفيروس نقص المناعة البشري لكل خمس أو خمس عشرة حالة لختان الذكور، كما أن تكاليف تفادي حالة إصابة واحدة بفيروس نقص المناعة البشري تتراوح بين 150$ و900$ على مدى عشر سنوات.
- وفي ظل الافتراضات المقبولة، توقعت النماذج أن يكون لكل من الاستئناف المبكر للمارسة الجنسية بعد عملية الختان والتعويض عن المخاطر السلوكية المقصورة على الرجال الذين تم ختانهم حديثاً أو المختنون من قبل وشركائهم آثار صغيرة على مستوى السكان فيما يتعلق بالأثر المتوقع للتوسع في خدمات ختان الذكورعلى حدوث الإصابات الجديدة بفيروس نقص المناعة البشري.
- واخيراً، أظهرت النماذج أن النساء يستفدن بشكل غير مباشر من انخفاض انتشار فيروس نقص المناعة البشري في شركائهن الرجال، كما اقترحت أنه بالرغم من أن التوسع في ختان الذكور لن يستطيع وحده أن يوقف وباء فيروس نقص المناعة البشري من الانتشار، إلا انه قد يتآزر مع استراتيجيات أخرى لخفض عبء الفيروس.
- لقد أمدت نتائج النمذجة صانعي القرار بالافتراضات الأساسية الخاصة بأداة التخطيط العملية للبرامج المعنية بختان الذكور.
