# Supplementary material for: Male Circumcision for HIV Prevention in High HIV Prevalence Settings: What Can Mathematical Modelling Contribute to Informed Decision Making?
Source: PLoS Med. 2009 Sep 8;6(9):e1000109. doi: 10.1371/journal.pmed.1000109 (PMC2731851; doi:10.1371/journal.pmed.1000109)
Supplement: Alternative Language Summary S2 — Chinese translation of the abstract by Yuhua Fan. (0.03 MB DOC) [file pmed.1000109.s002.doc]

- 数学模型能估计出在艾滋病高流行地区，男性包皮环切术对人群的艾滋病发病率的潜在影响；然而，不同的方法、最初假设和模型中输入的变量可能为决策者提供迥异的结果。
- 为了帮助决策者们, 联合国艾滋病规划署、世界卫生组织和南非流行病模型和分析中心最近邀请了一些专家，回顾和比较了六个模拟模型所得出的结果，这些模型中包含了对政策制定和项目决策至关重要的八个问题。
- 这些模型产生了一些相似的发现：在艾滋病高流行率以及包皮环切术并不普遍的地区，包皮环切术可以为异性性交的男性带来很大的益处，每5-10个包皮环切术可以防止1例艾滋病感染；按照10年的时间跨度进行计算，防治1例艾滋病传染的预防成本为150-900美元。
- 在合理的推定下，这些模型推测,尽管可能有一些刚刚做完包皮环切术或已做包皮环切术的男性及其配偶，在术后过早恢复性交或由于认为感染风险减少而有不安全的性性行为发生，但是从总体人群角度考虑,这些负面影响同包皮环切术推广的预期正面效果相比是很小的。
- 最后,这些模型显示,由于男性伴侣艾滋病感染率下降，女性也可以间接地受益。虽然单纯推广男性包皮环切术，不会遏制艾滋病的流行，但是它可以和其他的策略协同作用，共同减少和降低艾滋病所导致的负担。
- 这些模拟结果影响了一个重实效的决策者在计划男性包皮环切术项目方案时所采取的内在假定.
