# Supplementary material for: Male Circumcision for HIV Prevention in High HIV Prevalence Settings: What Can Mathematical Modelling Contribute to Informed Decision Making?
Source: PLoS Med. 2009 Sep 8;6(9):e1000109. doi: 10.1371/journal.pmed.1000109 (PMC2731851; doi:10.1371/journal.pmed.1000109)
Supplement: Alternative Language Summary S3 — Danish translation of the abstract by Nicolai Lohse. (0.04 MB DOC) [file pmed.1000109.s003.doc]

Mandlig omskæring for forebyggelse af HIV i områder med høj HIV-prævalens: hvordan matematisk modellering kan bidrage til informeret beslutningstagning

- Matematiske modeller kan estimere den potentielle populationsbaserede effekt af mandlig omskæring på incidensen af HIV i områder med høj HIV-prævalens, men forskelle i metoder, basale antagelser og input-variable kan dog betyde at modellernes resultaterne ikke bliver entydige for beslutningstagere.
- For at hjælpe beslutningstagere samlede UNAIDS, WHO og SACEMA for nylig en ekspertgruppe med det formål at gennemse og sammenligne resultater fra seks matematiske modeller vedrørende otte spørgsmål som er væsentlige for politisk og programmatisk beslutningstagning.
- Modellernes resultater var sammenlignelige, nemlig at fordelene ved omskæring af heteroseksuelle mænd i områder med lav forekomst af mandlig omskæring og høj forekomst af HIV vil være store med én HIV-infektion undgået for hver 5 til 15 udførte omskæringer, og at udgifterne for hver undgået HIV-infektion over en 10-årig periode vil være mellem 150 og 900 US dollars.
- Under rimelige antagelser forudsagde modellerne, at hvis præmatur postoperativ genoptagelse af seksuel aktivitet samt risikokompenserende adfærdsændringer er begrænset til nyligt eller allerede omskårne mænd og deres partnere, vil dissse faktorer have beskeden indflydelse på den forventede populationsbaserede ændring i HIV-incidens som ville følge af en stigning i antallet af mandlige omskæringer.
- Endelig viste modellerne at mandlig omskæring indirekte vil komme kvinder til gode gennem lavere HIV-prævalens i mandlige partnere, og selvom øgede omskæringsaktiviteter ikke alene vil stoppe en HIV-epidemi vil de kunne virke synergistisk med andre forebyggelsesstrategier til at reducere byrden af HIV-sygdom.
- Modellernes resultater har bragt information til de bagvedliggende antagelser i et pragmatisk planlægningsværktøj for mandlige omskæringsprogrammer.

Translation: Nicolai Lohse
