# Supplementary material for: Male Circumcision for HIV Prevention in High HIV Prevalence Settings: What Can Mathematical Modelling Contribute to Informed Decision Making?
Source: PLoS Med. 2009 Sep 8;6(9):e1000109. doi: 10.1371/journal.pmed.1000109 (PMC2731851; doi:10.1371/journal.pmed.1000109)
Supplement: Alternative Language Summary S4 — French translation of the abstract by Jacqueline Rossel. (0.03 MB DOC) [file pmed.1000109.s004.doc]

Francais:

*       Les modèles mathématiques peuvent estimer les répercussions potentielles de la circoncision masculine sur l’incidence du VIH dans des populations où la prévalence du VIH est élevée. Toutefois, selon les méthodes, les hypothèses et les paramètres utilisés, ces modèles peuvent aboutir à des conclusions contradictoires pour les décideurs.

*      Pour aider ces derniers, L’ONUSIDA, l’OMS et le SACEMA ont récemment réuni des experts pour examiner et comparer les résultats donnés par six modèles de simulation répondant à huit questions clés pour la prise de décisions politiques et programmatiques.

*       Ces modèles ont produit des résultats comparables: lorsque le taux de circoncision est faible et que la prévalence du VIH est élevée, les avantages de la circoncision pour les hétérosexuels sont importants avec une infection à VIH évitée pour 5 à 15 circoncisions et avec un coût de 150$ à 900$ par infection à VIH évitée sur une échelle de temps de 10 ans.

*       Sous des hypothèses réalistes, les modèles ont prédit que la reprise prématurée postopératoire des rapports sexuels ainsi que la compensation des risques comportementaux des hommes récemment ou déjà circoncis, et leurs partenaires, n’ont que peu d’effets à l'échelle de la population sur l’impact de la généralisation de la circoncision sur l’incidence du VIH.

*       Enfin, les modèles ont montré que femmes bénéficiaient de la diminution de la prévalence du VIH chez leur partenaire masculin et, bien que la généralisation de la circoncision ne puisse éradiquer à elle seule une épidémie de VIH, la circoncision agit de manière synergique avec les autres stratégies de prévention pour réduire les effets délétères imputables à l'infection à VIH.

*      Les résultats des modèles ont alimenté les hypothèses d’un outil pragmatique de planification d’aide à la décision pour la circoncision.
