# Supplementary material for: Male Circumcision for HIV Prevention in High HIV Prevalence Settings: What Can Mathematical Modelling Contribute to Informed Decision Making?
Source: PLoS Med. 2009 Sep 8;6(9):e1000109. doi: 10.1371/journal.pmed.1000109 (PMC2731851; doi:10.1371/journal.pmed.1000109)
Supplement: Alternative Language Summary S5 — Russian translation of the abstract by Elena Sannikova. (0.03 MB DOC) [file pmed.1000109.s005.doc]

Пункты резюме

• Математические модели могут оценивать потенциальное влияние популяционного уровня мужского обрезания на обусловленную ВИЧ заболеваемость в условиях высокой распространенности ВИЧ; однако различные методы, базовые допущения и исходные переменные могут привести к противоречивым результатам для тех, кто принимает решения.

• ЮНЭЙДС, ВОЗ и Южноафриканский центр эпидемиологического моделирования и анализа SACEMA созвали экспертов для рассмотрения и сравнения шести имитационных моделей по вопросам, имеющим ключевое значение для принятия решений в области политики и программ.

• Модели принесли сопоставимые результаты: польза от обрезания гетеросексуальных мужчин в условиях низкой распространенности мужского обрезания и высокой распространенности ВИЧ велика – на каждые 5-15 случаев обрезания приходится один случай преодтвращенной ВИЧ-инфекции, при этом стоимость предотвращения одного случая ВИЧ-инфекции варьируется от 150 до 900 долл. США в условиях 10-летнего временного горизонта прогнозирования.

• При правдоподобных допущениях, как преждевременное послеоперационное возобновление половой жизни, так и компенсация поведенческого риска, относящаяся только к вновь или уже обрезанным мужчинам и их партнерам, имеют лишь незначительное популяционное влияние.

• Женщины получают косвенную выгоду от снижения распространенности ВИЧ среди партнеров мужского пола, и хотя само по себе расширение масштабов мужского обрезания не способно прекратить эпидемию ВИЧ, оно действует в синергии с другими стратегиями для снижения бремени заболеваемости, связанной с ВИЧ.

__________
