# Supplementary material for: Male Circumcision for HIV Prevention in High HIV Prevalence Settings: What Can Mathematical Modelling Contribute to Informed Decision Making?
Source: PLoS Med. 2009 Sep 8;6(9):e1000109. doi: 10.1371/journal.pmed.1000109 (PMC2731851; doi:10.1371/journal.pmed.1000109)
Supplement: Alternative Language Summary S6 — Spanish translation of the abstract by Roger Biosca. (0.03 MB DOC) [file pmed.1000109.s006.doc]

- Los modelos matemáticos pueden estimar el efecto potencial de la circuncisión masculina sobre la incidencia del VIH en lugares de alta prevalencia; no obstante, diferentes métodos, supuestos y datos utilizados pueden producir resultados contradictorios para las personas responsables de tomar decisiones.
- El ONUSIDA, la OMS y el SACEMA convocaron a expertos para examinar y comparar seis modelos de simulación sobre cuestiones clave para la toma de decisiones de política y programáticas.
- Los modelos obtuvieron resultados comparables: las ventajas de la circuncisión masculina entre varones heterosexuales en lugares con bajo nivel de circuncisión y alta prevalencia del VIH fueron significativas, pues se evitó una infección por VIH por cada 5 a 15 circuncisiones masculinas realizadas y el costo de prevenir una infección por VIH osciló entre US$150 y US$900 en un escenario de 10 años.
- De acuerdo con supuestos verosímiles, tanto la reanudación posoperatoria prematura del coito sexual como la compensación del comportamiento de riesgo limitada a los varones recién circuncidados o ya circuncidados y sus parejas tienen tan sólo pequeños efectos en la población.
- Finalmente, los modelos muestran que las mujeres se benefician indirectamente de una menor prevalencia del VIH en sus parejas masculinas, y sugieren que aunque la circuncisión masculina se amplíe, por sí sola no puede contener una epidemia de VIH, pero puede actuar de forma sinérgica con otras estrategias para reducir la carga de morbilidad por el VIH.
- Los resultados de los modelos han proporcionado los principales supuestos para un instrumento para programas de planificación sobre circuncisión masculina para personas encargadas de la toma de decisión.
