# Supplementary material for: Male Circumcision for HIV Prevention in High HIV Prevalence Settings: What Can Mathematical Modelling Contribute to Informed Decision Making?
Source: PLoS Med. 2009 Sep 8;6(9):e1000109. doi: 10.1371/journal.pmed.1000109 (PMC2731851; doi:10.1371/journal.pmed.1000109)
Supplement: Text S2 — Presentations from meetings held in Stellenbosch, November 2007 and London, March 2008. (4.28 MB PDF) [file pmed.1000109.s008.pdf]

Cost of the roll-out of male circumcision in sub-Saharan Africa

Bertran Auvert	INSERM	France
Jim Kahn	UCSF	USA
Eline Korenromp	The Global Fund	Switzerland
Jamie Llyod-Smith	Pennsylvania State University	USA
Stephane Helleringer	University of Pennsylvania	USA
Remi Sitta	INSERM	France
Dirk Taljaard	Progressus	South Africa
John Hargrove	SACEMA	South Africa
Brian Williams	WHO	Switzerland
Elliot Marseille	UCSF	USA

UNAIDS/WHO/SACEMA Consultation
Making Decisions on Male Circumcision for HIV Risk Reduction: Modelling the Impact and Costs
Stellenbosch, South Africa – 15-16 November 2007

Objectives

Key health economics estimates of the roll-out of MAMC* in sub-Saharan Africa:

a) Number of circumcisers needed

b) Cost of the roll-out (Discounted to present 3% annually)

c) Net cost (after adjustment for averted HIV medical costs)

d) Cost per HIV infection averted (cost-effectiveness)

e) Number of circumcisions to avoid one HIV infection

*MAMC=medicalized adult male circumcision

Methods

Geographical setting (country, province…)

HIV and demographics models

Demographics model

Cost model

HIV model

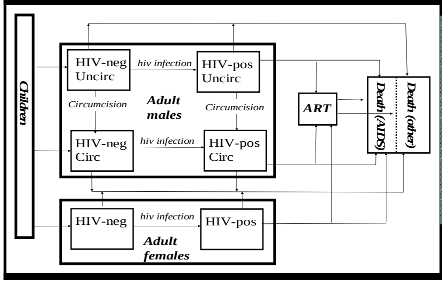

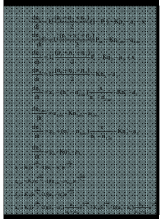

Differential equations

Input parameters

31 input parameters: One set per country
Sources: WHO, The Global Fund, OF, publications, opinion of experts

Input parameters		
Initial population size of adults in the geographic setting	6 260 000	
Birthrate	4.0	% per year
Percent of newborns reaching adulthood	70	%
Life expectancy when becoming adult (without HIV)	35	years
Adult males circumcised (in traditional setting)	10	%
Percent of males who will not accept circumcision	15	%
Duration to reach maximum male circumcision prevalence	5	years
Initial number of circumcisers	235	
Number of circumcisions per day per circumciser	10	
Number of working days per year	230	
MC effect (reduction of female to male transmission)	60	%
MC effect (reduction of male to female transmission)	10	%
Initial HIV prevalence among adults	24.6	%
Uncirc.male-to-female/f-to-uncirc.m ratio of transmissibility	1.5	
Percent of HIV(+) receiving treatment before ARV-eligible	30	%
Cost of this treatment (total)	799	US$
Percent of HIV(+)s eligible for ARVs who receive ARVs	30	%
Life expectancy on ARVs	13	years
Cost of ART (annual)	639	US$
Percent of HIV(+) eligible for ARVs who receive non-ARV treatment	30	%
Cost of this treatment (total)	1 764	US$
Discount rate (annual)	3.0	% per year
Cost model (1=Public 0=Private)	1	
Private: Geographic setting level (communication, management, M&E)	10	%
Private: Circumcision cost (inclusive)	22.0	US$
Public: Geographic setting level (communication, management, M&E)	20	%
Public: Initial investment per circumcision unit	7 482	US$ for year #1
Public: Number of circumcisers per circumcision unit	2	
Public: Initial training per circumciser	674	US$
Public: Salary of each circumciser	169	US$ per month
Public: Circumcision cost (variable)	10.4	US$

Output parameters

a) **Number of circumcisers needed**

b) **Cost of the roll-out** (Discounted to present 3% annually)

c) **Net cost** (after adjustment for averted HIV medical costs)

d) **Cost per HIV infection averted (cost-effectiveness)**

e) **Number of circumcisions to avoid one HIV infection**

Sensitivity analysis

Delta(input) → Delta (output)
Not presented

Range for each output

Monte Carlo simulation (aggregate uncertainty from all inputs)
Each input → range
random samples of input parameters → random samples of outputs
Range = 2.5 – 97.5 percentile

Input parameters: Demographics, HIV transmission, discounting

Initial population size of adults in the geographic setting	6 260 000	
Birthrate	4.0	% per year
Percent of newborns reaching adulthood	70	%
Life expectancy when becoming adult (without HIV)	35	years
Number of working days per year	230	
MC effect (reduction of female to male transmission)	60	%
MC effect (reduction of male to female transmission)	10	%
Initial HIV prevalence among adults	24.6	%
Uncirc.male-to-female/f-to-uncirc.m ratio of transmissibility	1.5	
Discount rate (annual)	3.0	% per year

Input parameters: Intervention

Adult males circumcised (in traditional setting)	10	%
Percent of males who will not accept circumcision	15	%
Duration to reach maximum male circumcision prevalence	5	years
Initial number of circumcisers	235	
Number of circumcisions per day per circumciser	10	
Number of working days per year	230	

Input parameters: Cost of the intervention

Private: Geographic setting level (communication, management, M&E)	10	%
Private: Circumcision cost (inclusive)	22.0	US$
Public: Geographic setting level (communication, management, M&E)	20	%
Public: Initial investment per circumcision unit	7 482	US$ for year #1
Public: Number of circumcisers per circumcision unit	2	
Public: Initial training per circumciser	674	US$
Public: Salary of each circumciser	169	US$ per month
Public: Circumcision cost (variable)	10.4	US$

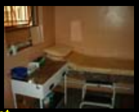

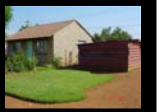

Input parameters: Cost HIV treatment

Percent of HIV(+) receiving treatment before ARV-eligible	30	%
Cost of this treatment (total)	799	US$
Percent of HIV(+)s eligible for ARVs who receive ARVs	30	%
Life expectancy on ARVs	13	years
Cost of ART (annual)	639	US$
Percent of HIV(+) eligible for ARVs who receive non-ARV treatment	30	%
Cost of this treatment (total)	1 764	US$

Where in sub-Saharan Africa ?

42 countries
Adult population = 331 million
Uncircumcised adult men= 54 million (33% of adult men)

To estimate the economic and human resources required for this roll-out :

Countries with “high” HIV prevalence (CEA criterion): > 5%
And
Countries with “low” MC prevalence < 80%

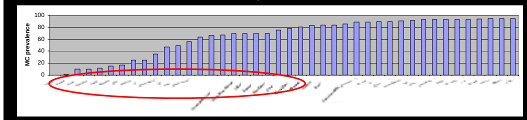

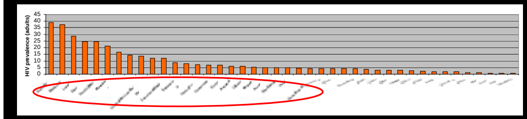

Where in sub-Saharan Africa ?

Countries: **42 countries** **→ 14 countries (33%)**
Uncircumcised men: **54 million** **→ 25 million (46%)**
HIV positive: **24 million** **→ 14 million (58%)**

	HIV (%)	MC (%)	Uncirc (million)	% total uncirc
**South Africa**	25	35	8.0	**32%**
**Zimbabwe**	25	10	2.8	11%
**Tanzania**	9	70	2.7	11%
**Malawi**	14	17	2.3	9%
**Zambia**	17	12	2.2	9%
**Mozambique**	12	56	2.0	8%
**Rwanda**	5	10	1.8	7%
**Burundi**	6	2	1.7	7%
**Lesotho**	29	0	0.4	2%
**Namibia**	21	15	0.4	2%
**Botswana**	37	25	0.3	1%
**CAR***	14	67	0.3	1%
**Liberia**	6	70	0.2	1%
**Swaziland**	39	50	0.1	1%
Total			***25.2***	*100%*

14 countries

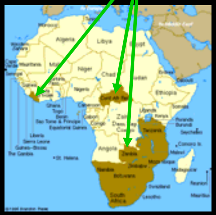

* Central African Republic

Conclusion

Preliminary results: Input data to be refined
(esp. private sector costs, currently exploratory only)

Expensive but reasonable in view of favorable cost-effectiveness and saving

Implementation model (private, public, both…) ?
(local context, availability…)

User friendly tool to be designed for use by decision makers, researchers and funders

Is cost the main issue ?

Costing: Where are we now?

Lori Bollinger

Prepared for:
UNAIDS/WHO/SACEMA Consultation
Making Decisions on Male Circumcision for HIV Risk Reduction: Modelling the Impact and Costs

Stellenbosch, South Africa – 15-16 November 2007

Futures INSTITUTE

MC costing studies

- 7 studies citing unit cost
  - All from Southern Africa
  - Quality of costing data varies
    - Not all studies have complete descriptions of intervention elements
      - Sometimes study focus is impact rather than cost
      - Exception: LSZ2 studies
  - Target population is adult males

Futures INSTITUTE

Summary of MC Studies

Country	*Year of data collection*	*Service delivery mode*
Kenya *	2005	Government & private
Lesotho	2007	Public/NGO hospitals
South Africa *	2003	Charge at GP offices
Swaziland	2007	Public/private hospitals, NGO clinic
Uganda	2006/7	n/a
Zambia 1 *	2003	Hospital
Zambia 2	2007	Public hospitals, NGO/private clinics

* Inflated to 2006 US$ (South Africa, inflated in study itself)

MC unit cost (US$)

Country	*Unit cost*	*General protocol*
Kenya	$20.78 – supplies only	5 visits
Lesotho	$45.35, $56.35 *	4 visits
South Africa	$55.76	Cost charged per surgery in GP office + complications + IEC ($5)
Swaziland	$40.30, $51.30 *	Cost per surgery including post-op care
Uganda	$69	Cost per surgery including post-op care
Zambia 1	$8.62 – supplies, not anesthesia or painkillers	n/a
Zambia 2	$35.79, $46.82 *	4 visits

* Comprehensive unit cost

MC costing studies (comprehensive unit costs)

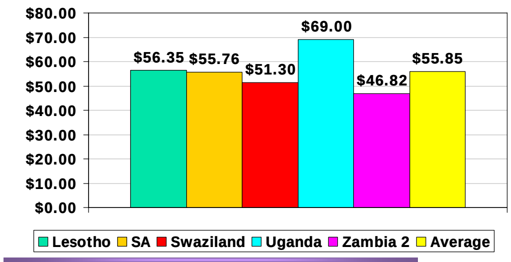

Futures INSTITUTE

Supplies portion of MC unit cost

Country	*Unit cost (supplies only)*	*Anesthesia*	*Antibiotics*	*Analgesics*	*Dressing applied visit #3*
Kenya	$20.78	Local	n/a	Paracetamol (5 days)+tramadol hydrochloride (2 nights)	n/a
Lesotho	$10.61	Local (private: general)	Routinely prescribed	Ibuprofen/ Paracetamol	100% re-applied
Swazi	$14.23	Local	Not routinely prescribed	Paracetamol	50% re-applied
Zambia 1	$8.62	Excluded	n/a	Excluded	n/a
Zambia 2	$9.81	Local	Not routinely prescribed	Paracetamol	Not re-applied

Marginal increase in unit cost due to treating complications

Country	*Marginal increase in unit cost*	*Type/frequency of complications*
Kenya	n/a	1.3% infections, 0.8% bleeds, 0.8% delayed healing/disrupted wounds, 0.4% swelling, 0.2% anesthetic reaction, 0.2% erectile dysfunction
Lesotho	$1.18	11% hemorrhage, 3% sepsis
South Africa	$1.04	3.7% short-term, 0.13% inpatient, 0.93% long-term
Swaziland	$0.74	2% hemorrhage, 5% sepsis
Zambia 2	$1.16	2.1% hemorrhage, 13% sepsis

Recommendation

- Use average unit cost of $55.85
  - In sensitivity analysis, vary unit cost from $47 to $69

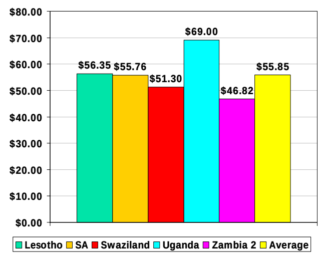

Observation #1:

- 23% of men reported having had sex since their circumcision at 30 days after surgery (Kenya)
- Conclusion: IEC materials *must* include information for sexual partners of circumcised men

Observation #2:

- Zambia: Private providers at University Teaching Hospital (UTH) in Lusaka recently were charging K350,000 vs. the K10,000 charge in public sector
  - US$89.45 vs. US$2.55
  - HPI Zambia study had private sector price of K200,000
- Conclusion: Pent-up demand exists

Costing: Where are we now?

Lori Bollinger

Prepared for:
UNAIDS/WHO/SACEMA Consultation
Making Decisions on Male Circumcision for HIV Risk Reduction: Modelling the Impact and Costs

Stellenbosch, South Africa – 15-16 November 2007

Understanding the Impact of Circumcision on the Heterosexual Spread of HIV in Zimbabwe

Tim Hallett*, Jennifer Smith, Simon Gregson & Geoff Garnett
Imperial College London

*timothy.hallett@imperial.ac.uk

Two Phases Of Work

1. With MoH/CDC in Zimbabwe:
   - Tool for analysing impact (for distribution)
   - Age targeting
2. Other work:
   - Quantifying uncertainty in the potential impact of MC interventions
     - In Zimbabwe
     - Generally…?

Three Models

- In common:
  - Deterministic representation of heterosexual transmission of HIV in 'risk'-stratified population: ODE/PDE
  - HIV in four stages + ART
  - Mixing can vary between "assortative" and "random" wrt risk.
  - Men can go: uncircumcised -> wound healing -> circumcised
  - Transmission depends on condom use / sex of infected partner / circ-status of man / risk-group of partners
  - MC intervention rolled-out over time
- Differences:
  - Model A: not age-structured, Excel spreadsheet for distribution
  - Model B: age-structured & parameterised for Zimbabwe (compiled code)
  - Model C: not age-structured, compiled code optimised for speed, designed for use in uncertainty analysis

---

Tool for analysing impact (for distribution)

(Model A)

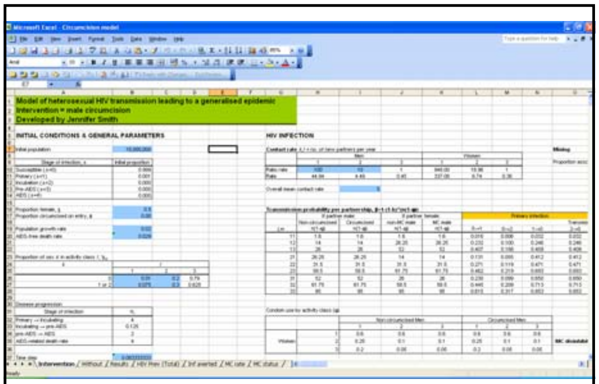

Excel Model

INPUTS

- Sexual behaviour
  - Mean and distribution of partner change rate
  - Pattern of mixing
  - Num sex acts and condom use in high and low-risk partnerships
- HIV transmission
  - Rate per act; varies by stage of infection
- Impact of interventions
  - Magnitude & timing (when start, max rate, speed of scale-up, character of men)
  - Biological effect (aq & tr / wound)
  - Behavioural changes (reduce condom use)

OUTPUTS

- Prevalence (t)
- Incidence (t)
- Infections averted (T)
- AIDS deaths averted (T)
- Number of operations (T)

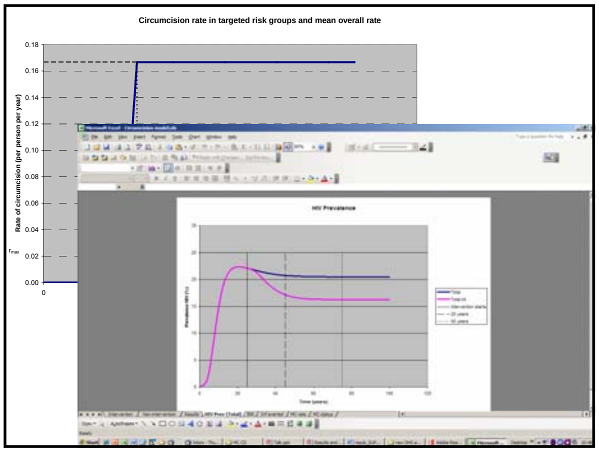

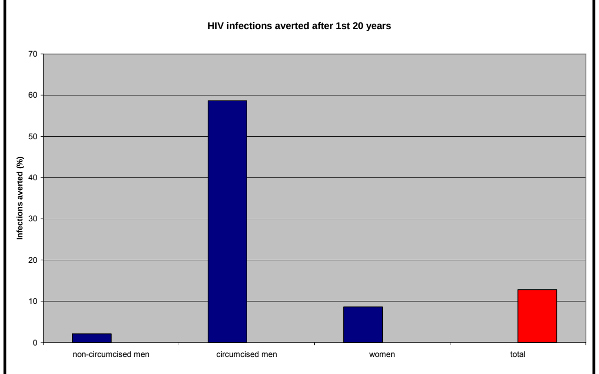

Result From Model:

- Basic epidemiological insights
- Basic predictions of impact of interventions
- Preliminary assessment of uncertainty about impact of circumcision interventions

DIRECT & INDIRECT Effects

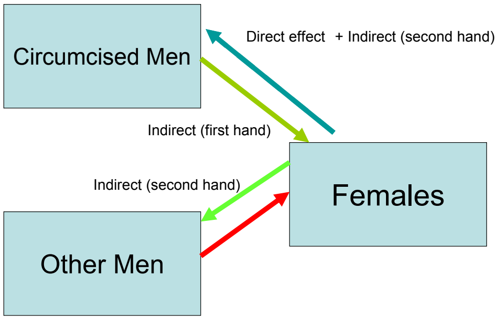

[Arrows show routes of transmission]

HIV infections averted over the first 20 years of the intervention

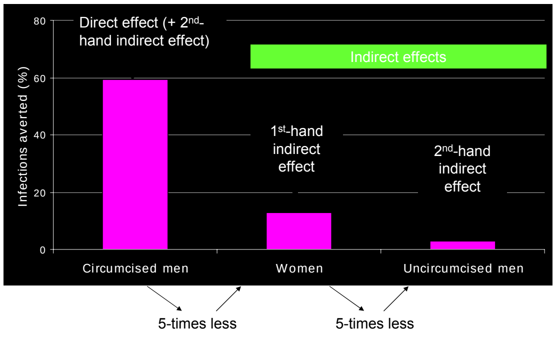

Programmatic Factors – coverage and timing

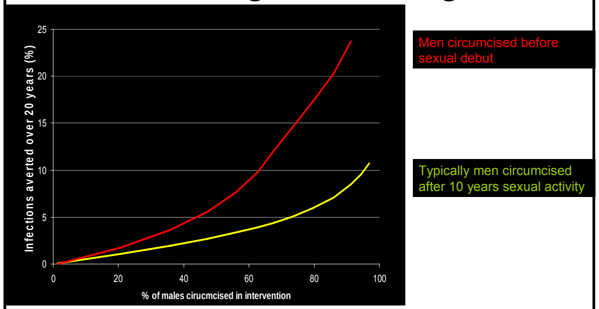

But, circumcision infants will have no impacts on epidemic for many years

Programmatic Factors

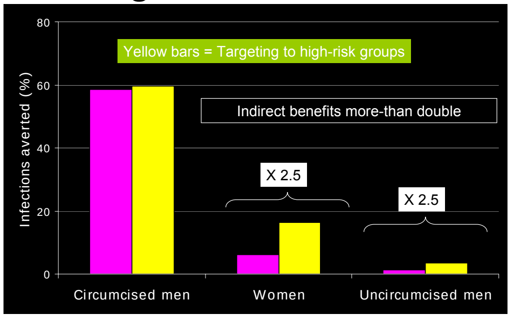

Biological factors

Green bars = Assuming 30% reduction in male-to-female transmission

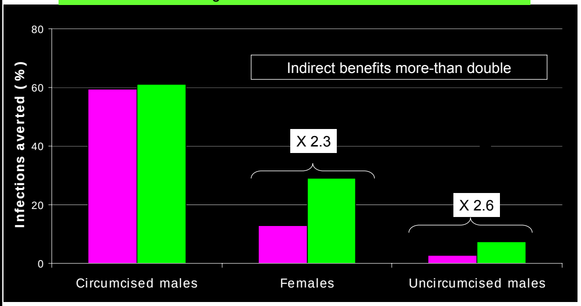

Behavioral factors

Behavioural disinhibition = fraction of men that do not use condoms after circumcision [keeping the same number of sexual partners]

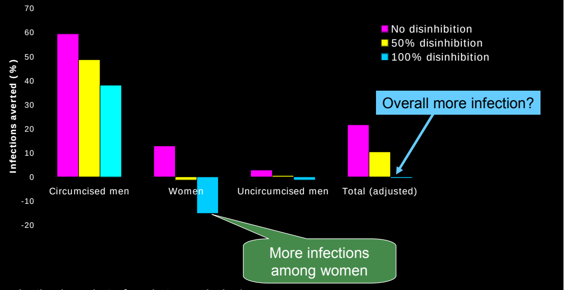

(No reduction in male-to-female transmission)

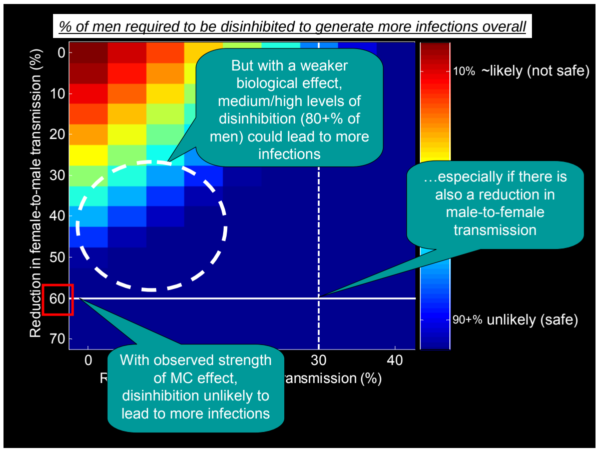

Age Targeting

At what age should men in Zimbabwe be circumcised?

The Model: Parameterised using Manicaland data

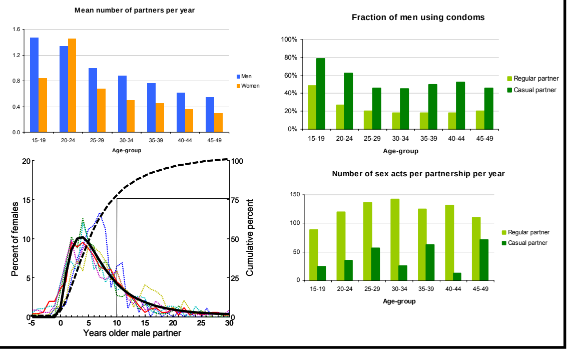

Baseline Model Output

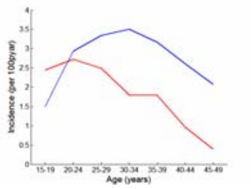

(DATA:)

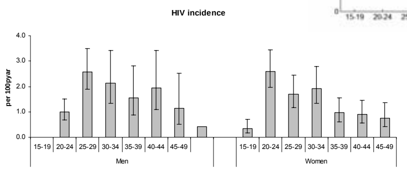

Impact of Alternative Age-targeting Interventions

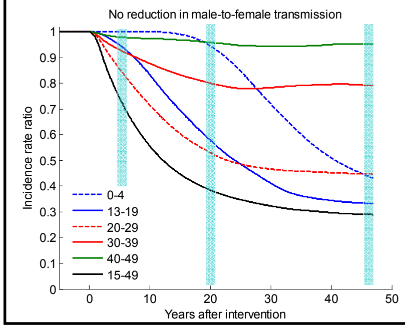

Best in short-term: 20-29s

Best in medium-term: 20-29s

Best in long-term: 13-19s

-- Ultra-long-term <5s

Impact of Alternative Age-targeting Interventions

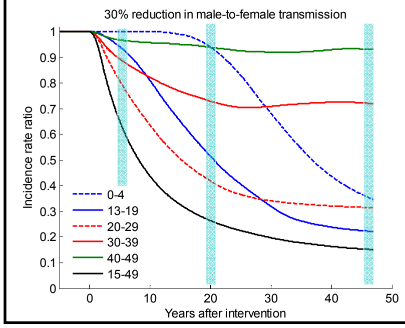

Best in short-term: 20-29s

Best in medium-term: 20-29s

Best in long-term: 13-19s

-- Ultra-long-term <5s

(Impact greater; relative benefit of circ old men increased; but overall conclusions - same)

Efficiency

~ Cumulative infections averted per cumulative operations*

Five years in

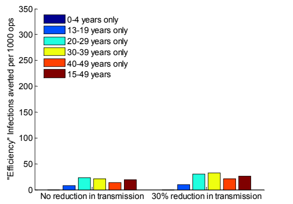

Ten years in

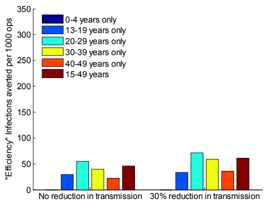

*Theoretical obstacle: not perfect comparison of intervention impact

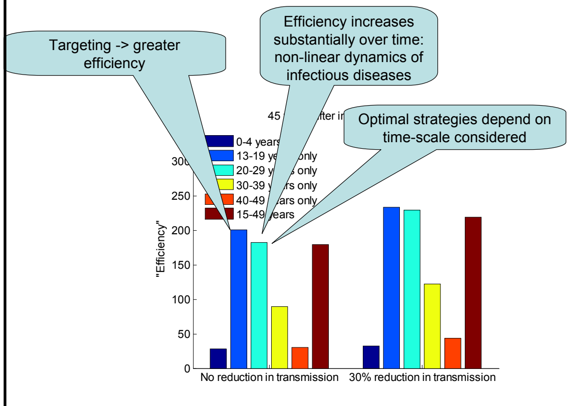

SLOW speed of intervention impact

In Zimbabwe, circumcision intervention not likely to contribute substantially to achieving targets in reducing HIV Prevalence.

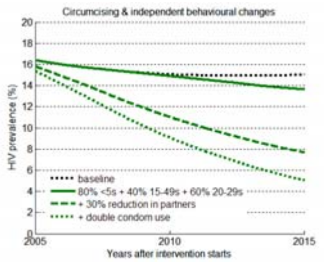

Why is it slow?

- Main (only) effect on reducing infection among men. INDIRECT effect therefore has to wait for prevalence to subside – which takes 5-10 years.
- Intervention cannot start at max capacity instantaneously. Here we assume 2 year roll-out.
- Roll-out among young boys and young men gives lead-time before any impact because they are not sexually active.

Quantifying uncertainty in the potential impact of MC interventions

Approach

- Aim: Calculate expected impact of intervention, quantify uncertainty and identify key determinants of impact
- To do this: Quantify variation in the many parameters that might have a bearing on the expected impact of the intervention (e.g. reduction in incidence).
- STEP 1: Fit model to Zimbabwe prevalence time series
- STEP 2: Apply range of interventions and measure effect.

Methods (Step 1)

- Background parameters determine profile of epidemic before intervention (e.g. condom use, partner change rate, mixing pattern, etc)
- Estimates of parameter value based on data from Manicaland (rural Eastern area). (Possible range of values determined by 95% confidence interval or credible range)
- Use LHS to generate 200,000 possible parameter combination.
- Reject those that do not generate epidemic consistent with observed prevalence trends.

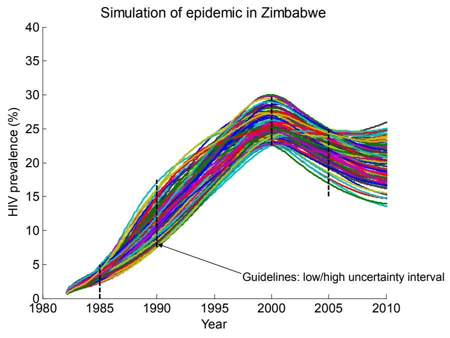

100 parameter-sets were found that were “consistent” with Zim prevalence curve.

Methods (Step 2)

- Quantify range of possible values for “intervention” parameters
  - Coverage of intervention: 80% of population (fixed for all simulations)
  - RR acquire infection if circumcised:

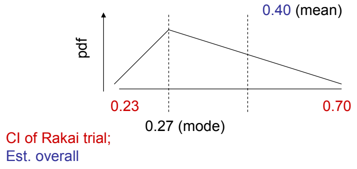

Methods (Step 2)

- RR transmit infection if circumcised:

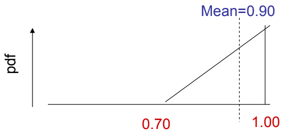

- Period of wound healing = 8 weeks (fixed)
- RR acquire infection during wound healing: 0.8-1.2 (mean=1)
- RR transmit infection during wound healing: 1-3 (mean=2)
- Fraction of men sexually active during wound healing: 5-20% (mean=10%)

Methods (Step 2)

- Risk compensation (=behavioural disinhibition):
  - Less condom use with non-regular partner
  - Less condom use with regular partner
  - More sexual partners

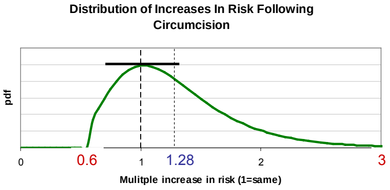

Methods (Step 2)

- Distribution of output will approximate expected distribution of predicted effect (quantifies uncertainty).

→ Can identify which parameters most determine the output.

→ Can quantify impact of changing selected parameters without specifying precise values for other parameters (analysis shows 'average' effect over all regions of parameter space).

- Generate 12,000 different intervention scenarios
- Pair randomly with baseline scenarios
- (→ each baseline scenario is tested with ~120 different intervention scenarios)

Model

- Deterministic ODE, solved numerically
- Population stratified by gender/risk/circumcision-status (not, wound-healing, circumcised)
- Pattern of sexual partnership formation determined by: "balance", "replacement" and "mixing" (-- *will explain* --)
- Sexual behaviour can change after circumcision (num partners, condom use with regular and/or non-regular partners)
- ART roll-out represented.

Model

Keep scale of intervention the same in all runs (=optimistic).

- Intervention scaled-up starts in 30 years after epidemic starts.
- Instantaneously get to full-scale
- 20% of sexually men get circumcised a year
- + 80% of men starting sex have already been circumcised

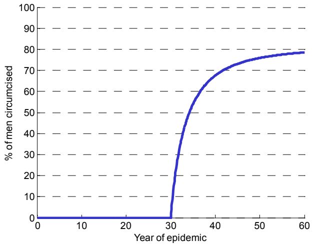

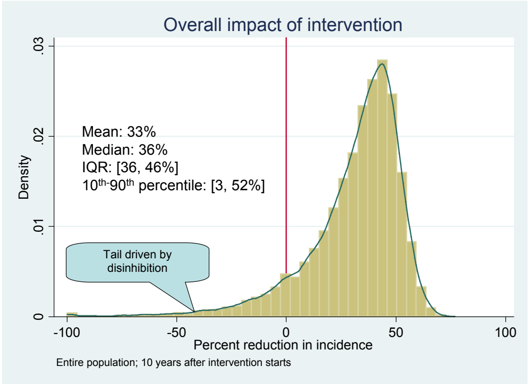

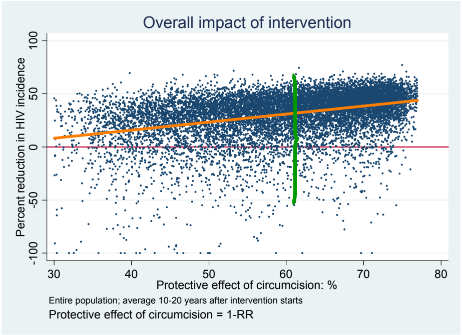

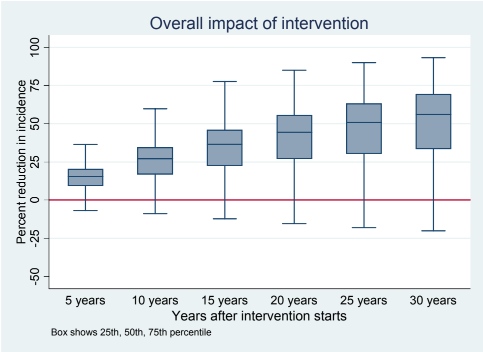

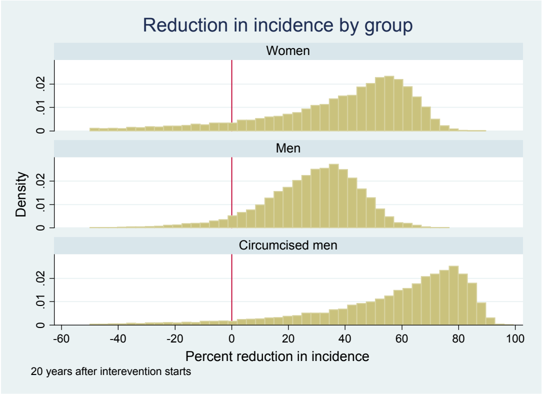

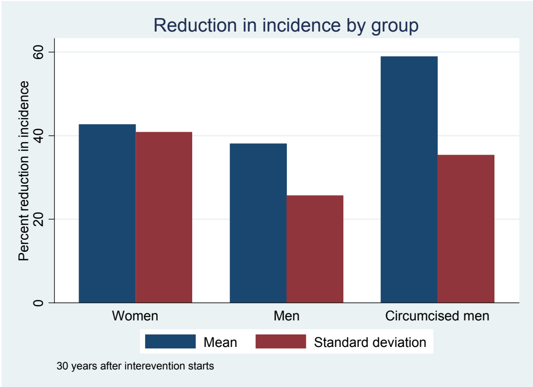

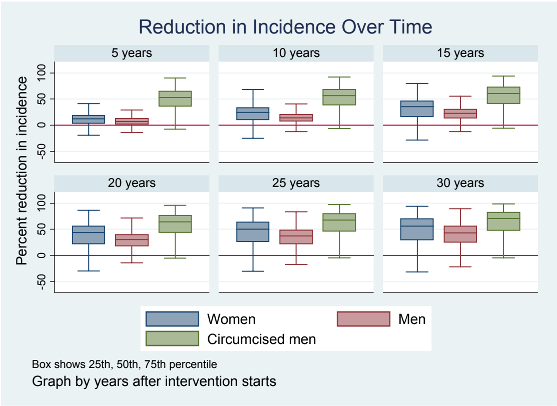

Sensitivity Analysis

With which parameters are the outcome most closely associated?

Two categories of parameters:

1. Background parameters (sexual behaviour)
2. Intervention parameters (biological and behavioural changes following circumcision)

Shown are the “Top Five” parameters in each category and overall

PRCC; partial rank correlation coefficients – independent association (monotonicity) between input and output variables, keeping other variables at their expected value.

Output= reduction in incidence across whole population, 30 years after intervention starts

**'All parameters:'**	"
'Increase in rate of partner change'	-0.74
'RR aquire infection if circumcised'	-0.72
'Increase risk with reg ptr if cirumcised'	-0.64
'Increase risk with non-reg if cirumcised'	-0.58
'RR transmit infection if circumcised'	-0.38
**'Background parameters'**	"
'Male control mixing'	-0.22
'Condom use in regular partnerships'	-0.16
'Recent changes in partner change rate'	-0.12
'Fraction of males in low risk group'	0.11
'Sex acts in non-regular partnerships'	-0.10
**'Intervention parameters'**	"
'Increase in rate of partner change'	-0.74
'RR aquire infection if circumcised'	-0.72
'Increase risk with reg ptr if cirumcised'	-0.64
'Increase risk with non-reg if cirumcised'	-0.58
'RR transmit infection if circumcised'	-0.38

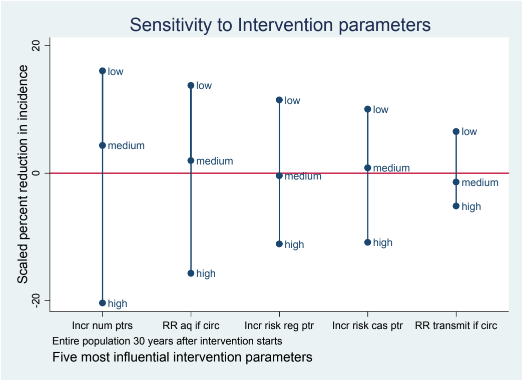

All

**'All parameters:'**	"
'Increase in rate of partner change'	-0.74
'RR aquire infection if circumcised'	-0.72
'Increase risk with reg ptr if cirumcised'	-0.64
'Increase risk with non-reg if cirumcised'	-0.58
'RR transmit infection if circumcised'	-0.38
**'Background parameters'**	"
'Male control mixing'	-0.22
'Condom use in regular partnerships'	-0.16
'Recent changes in partner change rate'	-0.12
'Fraction of males in low risk group'	0.11
'Sex acts in non-regular partnerships'	-0.10
**'Intervention parameters'**	"
'Increase in rate of partner change'	-0.74
'RR aquire infection if circumcised'	-0.72
'Increase risk with reg ptr if cirumcised'	-0.64
'Increase risk with non-reg if cirumcised'	-0.58
'RR transmit infection if circumcised'	-0.38

Men

**'All parameters:'**	"
'RR aquire infection if circumcised'	-0.68
'Increase risk with non-reg if cirumcised'	-0.60
'Increase risk with reg ptr if cirumcised'	-0.57
'Increase in rate of partner change'	-0.44
'RR transmit infection if circumcised'	-0.34
**'Background parameters'**	"
'Male control mixing'	-0.22
'Recent changes in partner change rate'	-0.20
'Condom use in regular partnerships'	-0.12
'Risk ratio in medium-risk group (males)'	-0.09
'Fraction of males in low risk group'	0.08
**'Intervention parameters'**	"
'RR aquire infection if circumcised'	-0.68
'Increase risk with non-reg if cirumcised'	-0.60
'Increase risk with reg ptr if cirumcised'	-0.57
'Increase in rate of partner change'	-0.44
'RR transmit infection if circumcised'	-0.34

Women

**'All parameters:'**	"
'Increase in rate of partner change'	-0.79
'RR aquire infection if circumcised'	-0.74
'Increase risk with reg ptr if cirumcised'	-0.66
'Increase risk with non-reg if cirumcised'	-0.55
'RR transmit infection if circumcised'	-0.40
**'Background parameters'**	"
'Male control mixing'	-0.17
'Condom use in regular partnerships'	-0.15
'Recent changes in partner change rate'	-0.13
'Risk ratio in medium-risk group (males)'	-0.09
'Sex acts in non-regular partnerships'	-0.08
**'Intervention parameters'**	"
'Increase in rate of partner change'	-0.79
'RR aquire infection if circumcised'	-0.74
'Increase risk with reg ptr if cirumcised'	-0.66
'Increase risk with non-reg if cirumcised'	-0.55
'RR transmit infection if circumcised'	-0.40

Circumcised Men

**'All parameters:'**	"
'RR aquire infection if circumcised'	-0.83
'Increase in rate of partner change'	-0.82
'Increase risk with non-reg if cirumcised'	-0.65
'Increase risk with reg ptr if cirumcised'	-0.60
'RR transmit infection if circumcised'	-0.25
**'Background parameters'**	"
'Male control mixing'	-0.15
'Recent changes in partner change rate'	-0.14
'Condom use in regular partnerships'	-0.13
'Sex acts in non-regular partnerships'	-0.13
'Fraction of males in low risk group'	0.07
**'Intervention parameters'**	"
'RR aquire infection if circumcised'	-0.83
'Increase in rate of partner change'	-0.82
'Increase risk with non-reg if cirumcised'	-0.65
'Increase risk with reg ptr if cirumcised'	-0.60
'RR transmit infection if circumcised'	-0.25

Risk compensation

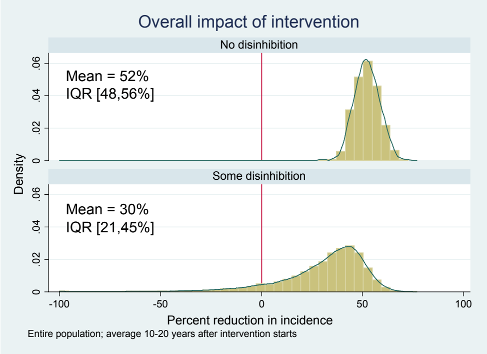

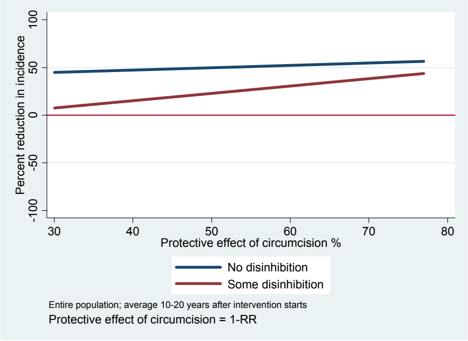

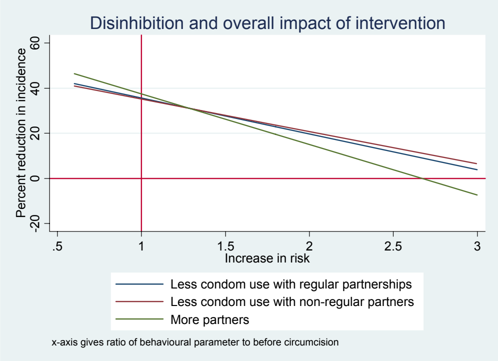

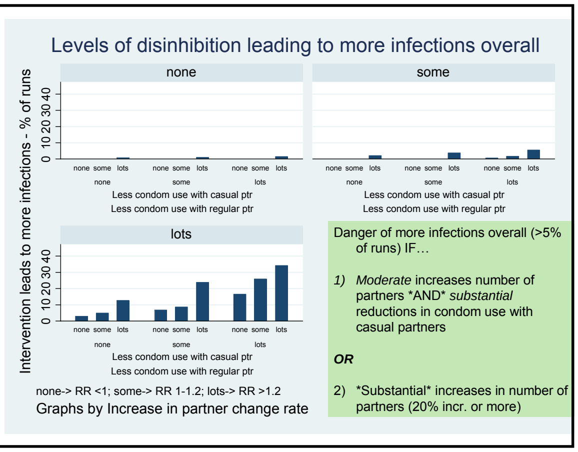

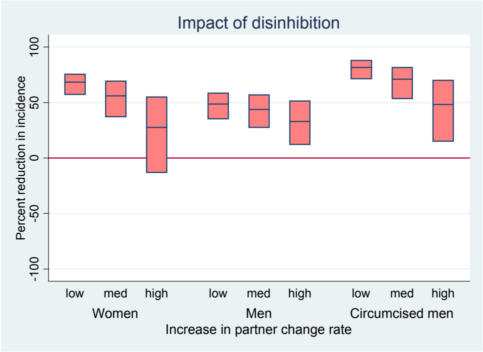

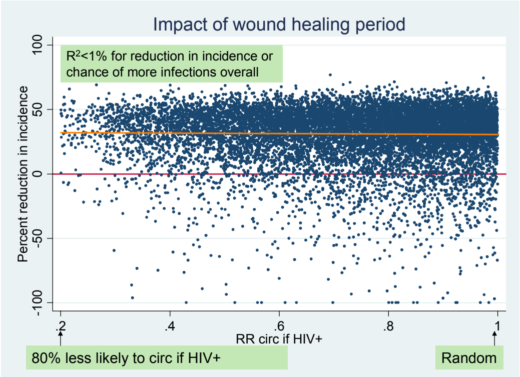

General Uncertainty Analysis

- Which of these conclusion are specific to Zimbabwe?
- How much does information of sexual behaviour/prevalence in Zimbabwe help refine predictions?
- Can an "overall" measure of the impact of circumsion interventions be quantified?

ALL BACKGROUND PARAMETERS CONSIDERD (NOT JUST ZIMBABWE)

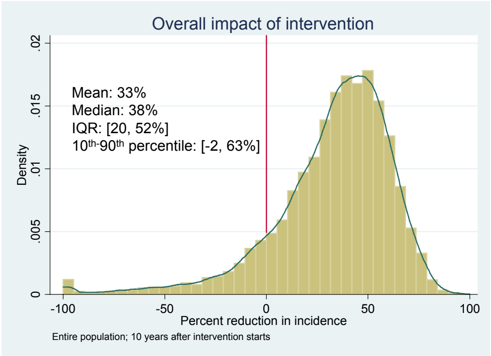

Conclusions

- Impact of circumcision at population level mediated by DIRECT and INDIRECT effects.
- One component of the DIRECT EFFECT (on reduced risk of acquisition) has been quantified by RCTS. The other component (transmission) is not known. The magnitude of INDIRECT EFFECTS depend on the sexual behaviour and the pattern of risk in the population – also not known.
- Therefore, there is considerable uncertainty in the impact of the intervention – but we can make an attempt to quantify this using mathematical models.

Conclusions

- Across all settings:
  Over 10-20 years, circumcision likely to reduce HIV incidence by 20-52% (IQR). This includes:
  - Incidence among women reduced by: ~36%
  - Incidence among circumcised men reduced : ~53%
- In Zimbabwe:
  Over 10-20 years, circumcision likely to reduce HIV incidence by 36-46% (IQR). This includes:

Conclusions

- The impact of circumcision will emerge slowly
  - First among circumcised men
  - Then among women
  - Then among other men

  (Faster if direct effect on transmission)
- There is a chance that the intervention will lead to more infection overall if there is substantial behavioural disinhibition following circumcision.

Conclusions (…cntd)

- For each demographic group and overall, the most important determinant of impact is the extent of risk compensation – especially increases in partner numbers.
- Other important not-quantified determinants are:
  - Changes in condom use – especially with casual partners
  - Rate of transmission from infected circumcised men
- Properties of sexual network have relatively less influence than properties of intervention.

  (Interactions “intervention*epi-context” not yet explored)

Conclusions (…cntd)

- Behavioural changes following circumcision could substantially reduce impact of intervention
- Reduces the impact for women, especially.
- Worst types are: more partners & less condom use with casual partners
- Can even lead to more infections overall if, either:
  - *Moderate* increases number of partners *AND* *substantial* reductions in condom use with casual partners.
  - *VERY LARGE* (>20%) increases in partner change rate

Conclusions (…cntd)

- Age targeting: young men/boys for long-term impact/ 20-29s for short-term
- Optimality will depend on time-scale considered and on age-pattern of acceptance.
- Targeting to this age-range will lead to more infections averted for the same number of operations.
- Infections averted per operation will increase over time, as indirect impact of intervention spreads through population

Spare slides

Output For Comparison Of Alternative Intervention Strategies

- **Cumulative infections averted?***
  - Population size confounds
  - Successful intervention-> Less AIDS burden->Bigger Population->More infections despite lower incidence rate?
- **Incidence rate ratio at time *t*?***
  - Risk distribution confounds?
  - No intervention -> highest risk depleted; average incidence dominated by incidence rate among low-risk groups
  - Successful intervention -> highest risk groups not depleted; average incidence biased upward?
  - Extent will depend on distribution of risk and assumptions made about ‘replacement’
- **Adjusted incidence rate ratio at time *t*?**
  - Incidence rate measured in intervention simulation
  - Compare with incidence rate measured in control simulation – but standardising the risk distribution.
  - i.e. effectively “risk-matched” incidence rate ratio (“How much lower is incidence in the intervention simulation, for each demographic/risk group – then average”)
  - This used for most outputs…

*NB. These measures OK if only interested in making “prediction” – not necessarily fair comparisons

True protective effect of circumcision per act: 60%

$$s = (1 - p_a)^n$$

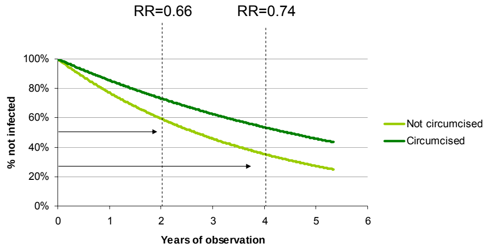

(based on five sex acts per act)

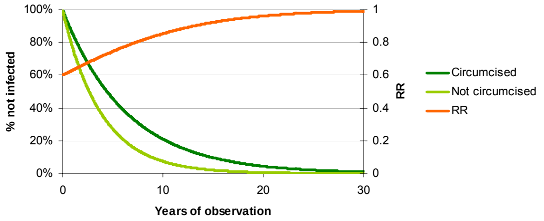

Estimating protection per-act?

Rakai trial	
alpha (transmision: not circ)	0.001
P (fraction infected: not circ)	2.6%
P-c (fraction infected: circ)	1.1%
lambda	0.4199
alpha-c (transmission: circ)	0.0004
% protection per sex act:	58

$$p = 1 - (1 - \alpha)^n$$
$$p_c = 1 - (1 - \alpha_c)^n$$

$$n = \frac{\ln(1-p)}{\ln(1-\alpha)} = \frac{\ln(1-p_c)}{\ln(1-\alpha_c)}$$

$$\ln(1-\alpha_c) = \ln(1-\alpha)\left[\frac{\ln(1-p_c)}{\ln(1-p)}\right]$$

$$\alpha_c = 1 - (1-\alpha)^\lambda$$
$$\lambda = \frac{\ln(1-p_c)}{\ln(1-p)}$$

Rates are so low- bias caused by this is negligible.

Risk:	High	Medium	Low
Num partnerships per year	50	5	1
Sex acts per partnership	2	15	100
Condom use in each partnerships	0.6	0.25	0.05
Number of unprotected sex acts	40	56.25	95
Fraction when exposed to HIV	0.75	0.6	0.05
Unprotectd sex acts, exposed to HIV	30	34	5
% infected - no circ	3.0%	3.3%	0.5%
% infected - circ (60% protection)	1.2%	1.3%	0.2%
Potential reduction in transmission with cirucmcision	59.7%	59.6%	60.0%

Affects all risk-strata equally

Cost-Effectiveness of Male Circumcision for HIV Prevention in a South African Setting … and beyond

Jim Kahn & Elliot Marseille, UCSF
Bertran Auvert, University of Versailles

Background

- RCT in Orange Farm showed a protective effect for adult male circumcision of 60%, consistent with observational studies.
- Resources for HIV prevention are limited, and the economics of a biological intervention of this type are unknown.

Goals

- Assess the cost-effectiveness of MC for Gauteng Province, South Africa
- Use the analysis model to estimate cost-effectiveness in sub-Saharan African settings with different epidemiology or costs.

MC CEA Outcomes

- HIV Infections averted
- Cost per HIV infection averted
  - Unadjusted for averted lifetime cost of HIV treatment
  - Adjusted for averted lifetime cost of HIV treatment

MC CEA inputs

- Cost of each MC, including promotion
- Frequency and cost of side effects
- HIV prevalence and incidence in men
- Protective effect of MC
- Reduction of protective effect due to risk compensation
- Multiplier effects due to epidemic dynamics, e.g. protection to women
- Savings from medical costs averted

CE base case input values

- MC $55 each, mild side effects 5% / $13, serious side effects 0.4% / $334
- HIV prevalence 25.6%, incidence 3.8 /100/yr (> 18 y)
- Protective effect 60% female to male
- Risk compensation 25%
- Epidemic multiplier 1.5
- Not included: effect on male-to-female transmission risk & reduced cases in infants
- Lifetime cost of HIV treatment $8000 (50% ART)
- 20 years

Modeling

- **Intent:**
  - *Flexibly portray effects* over time on HIV infections averted and CE of: HIV prevalence & incidence, MC cost, MC protective effect, risk compensation, 2er benefits.
  - *Simplicity*: close to data, transparent.
  - *Uncertainty of modeling << key inputs*. Limitation: low granularity.
- **Tools:**
  - *Linear extrapolation* of averted infections as function of reduced annual incidence.
  - *Interval epidemic model* with 6 compartments (male circ'd / not, female; HIV infected / not).

CE base case results

Program cost per MC	
Cost of male circumcision	$ 54.72
Cost of adverse events	$ 1.03
Total cost	$ 55.75
HIV infections averted per MC	
Undiscounted	0.43
Discounted	0.31 (3 MCs per HIA)
**Cost-Effectiveness**	
Cost per HIV infection averted (unadjusted for averted medical costs)	$181
Net cost, adjusted for averted medical costs (savings)	($2,411)

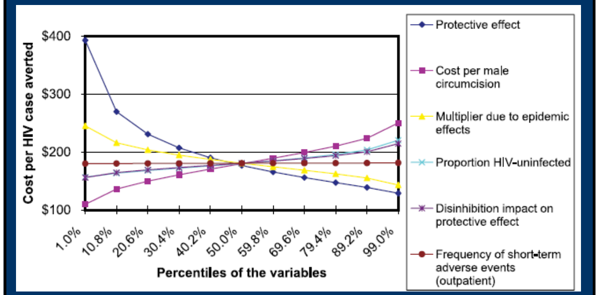

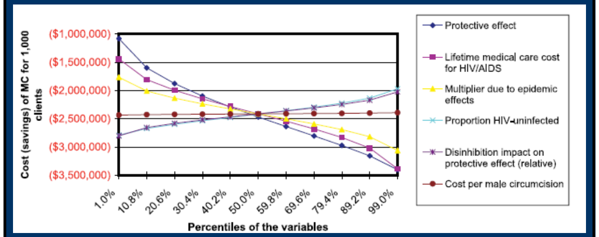

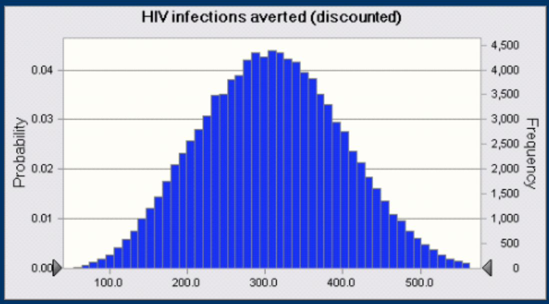

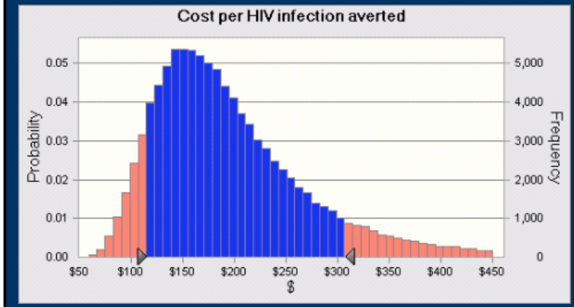

Certainty level 80%

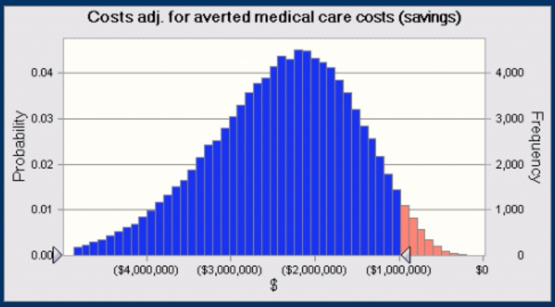

Certainty level 96.5%

Table 3. Three-Way Sensitivity Analysis

Multiplier Value	Protective Effect	Cost per HIA ($) in Three Unit Cost Groups		
		Unit Cost $30	Unit Cost $50	Unit Cost $100
Epidemic multiplier = 1.0	40%	350	545	1,031
	50%	234	363	688
	60%	175	271	516
	70%	140	218	413
Epidemic multiplier = 1.5	40%	234	363	688
	50%	156	242	458
	60%	117	181[a]	344
	70%	93	145	275
Epidemic multiplier = 2.0	40%	175	273	516
	50%	117	182	344
	60%	88	136	258
	70%	70	109	206

(all => *net savings* when adjusted for averted medical costs)

Alternate Epidemic Scenarios

- Base case: HIV prevalence 25.6%, incidence 0.038
- *Lower steady state:* With HIV prevalence = 8.4% & incidence = 0.01, cost per HIA is $551, with net savings of $753 per MC.
- *Declining epidemic:* With HIV prevalence = 25.6% but incidence = 0.01, cost per HIA is $1200, with net savings of $264 per MC.
- *Focus on young men:* Age 18-24, HIV prevalence = 10% and incidence = 0.021, cost per HIA = $135.

Program Scenarios

- *Recruitment at $5 per eligible* but only 10% coverage => $100 per MC performed, with net savings of $253 per MC.
- *To reach net cost of $0*, cost per MC must rise 45-fold to $2,466 (or protective effect must drop to 21%).
- *Scale* has <1% effect on impact (might lower costs)

Cost per HIV Infection Averted Across Prevention Interventions

• Adult MC	$ 181
• VCT	$ 393 - 482
• PMTCT	$ 20 – 21,000
• Condoms	$ 11 – 2108
• Peer educa SWs	$ 68 - $79
• Mass media	$ 58 (maybe)
• Rx of STI	$ 271-$514 (if effective)
• School-based	$ 7,288 - $13,326

Bias

- Favorable:
  - In epidemic model, we assume constant level of circumcision in cohort with replacement without ongoing cost. Impact lessened by discounting. Approx 1.5-fold bias. I.e., $270 per HIA.
- Unfavorable :
  - We costed MC for HIV+ (adds 33% in base case)
  - MC protective effect = 0.60; per receipt of MC = 76%.
  - Time horizon 20 years, so some censoring.
- Balance:
  - Neutral?

Conclusions

- Adult male circumcision in Sub-Saharan Africa appears to save money for a wide range of epidemic and economic conditions, and to compare favorably in cost-effectiveness with other HIV prevention strategies.
- Analyses should be updated from operating programs – costs (by MC delivery model: inputs, prices, efficiency in practice), complications, protective effect, risk compensation.

Rakai modeling and beyond

Tom Lutalo
Rakai Health Sciences Program

UNAIDS/WHO/SACEMA Consultation
Making Decisions on Male Circumcision for HIV Risk Reduction: Modelling the Impact and Costs
Stellenbosch, South Africa 15th Nov 2007

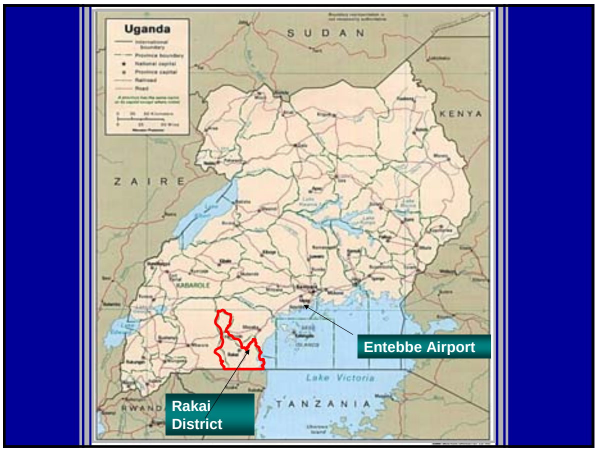

Potential impact of male circumcision: Stochastic simulation model from Rakai

What is the potential effect of circumcision on:

- HIV incidence in the population
- Course of the HIV epidemic (reproductive #: R_o)
- Number of circumcisions needed to avert HIV infection
- Cost per HIV infection averted

Study design and setting

- Based on data from a cohort in Rakai
- A stochastic model with empirically derived parameters used
  - Estimate the population-level incidence of HIV as a function of efficacy of male circumcision
- Rakai cohort has a mature generalized epidemic
  - Adult prevalence 11%
  - HIV incidence 1.24/100 py
- Probability of HIV transmission per sex act was computed

Participants

- Age categories 15-24, 25-29, 30-34, 35+
- Gender specific rates of partner change based on Rakai cohort data
  - 8% of HIV-infected women &
  - 53% of HIV infected men , reported multiple partnerships in a given year
- In Rakai 84% of men were uncircumcised

The Intervention

Impact of MC on HIV incidence and cost per infection averted:
A stochastic simulation model
Gray et al AIDS 2007

- Used empirical data on transmission probability by stage of disease and HIV viral load, age and gender
- Assumed Cx reduces HIV acquisition by an IRR of 0.3-0.6 in men, female partners and both sexes combined.
- An IRR of 0.7 for reduction in acquisition by women was also used.
- Assume varying circumcision coverage levels (0-100%) of HIV –ve uncircumcised men
- A cost per Circumcision of $69 including post operative care.

- Projected incidence over 10 years
- The model simulated HIV transmission from an HIV positive to an HIV negative partner

Primary Outcomes

- Estimates used to calculate cost and number of procedures required per HIV infection averted over a 10 yr period
- The epidemic reproductive number (R_o) was estimated
- For behavioral disinhibition, estimates were derived for increasing numbers of sexual partners per individual (by 25%, 50% or 100%)

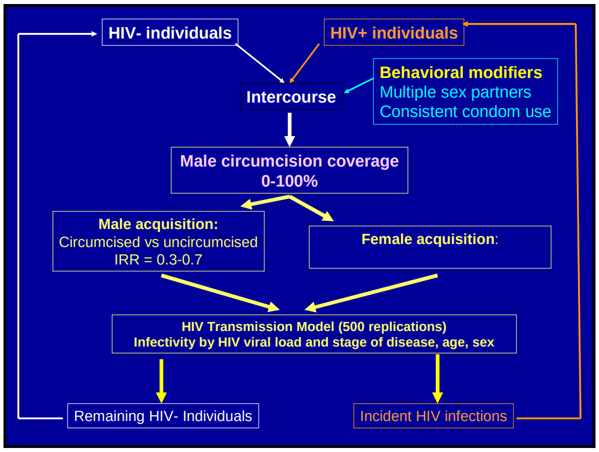

Population HIV incidence by circumcision: reduction in male HIV acquisition, and program coverage

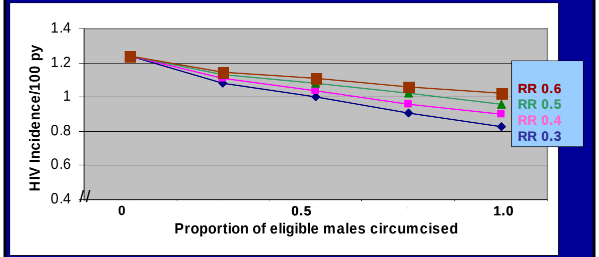

(Gray *et al AIDS 2007*)

R_0 ~ 1.0 if coverage 100%

HIV incidence and R_o by efficacy of circumcision and 75% program coverage

IRR	Male acquisition	
	Incidence	R_o
0.3	0.91	1.01
0.5	1.02	1.14
0.6	1.16	1.27

Initial incidence 1.24/100 py, R_o = 1.44

Effect of behavioral disinhibition on HIV incidence, by circumcision IRR in both sexes combined, 75% coverage

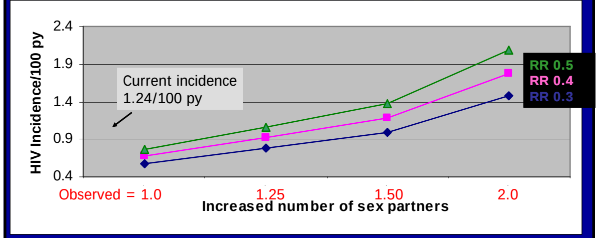

Behavioral disinhibition can offset all benefit, even at high circumcision efficacy

Cost per infection averted

- Requires:
  - An estimate of the infections averted by circumcision over time, with varying efficacy and assumptions about coverage
  - Unit cost of surgery, postoperative and management of adverse events (~$69.0 in Rakai trial)

Estimates of number of surgeries per HIV infection averted in the trial

- Based on 24 month transmission risks of 2.6% in the control arm and 1.11% in the intervention arm. Risk difference 1.49%
- Infections averted = 1/risk diff = 67 surgeries per HIV infection averted over two years
- Rakai
  - HIV prevalence ~ 11%
  - Incidence in uncircumcised men ~ 1.3/100 py

Cost of male circumcision for HIV prevention in Rakai

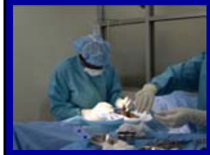
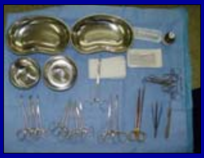
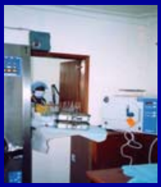

Rakai: Cost per circumcision ~ $69.0 for surgery, postoperative care and AE management
Research study using physicians, fully equipped theaters and disposables.
Program costs are likely to be lower (assume $35)

Cost per infection averted in the trial

- Cost $69 per surgery
- Surgeries per HIV infection averted = 67
- Cost per infection averted = $4623
  - Ignores possible secondary reduction in transmission to female partners
  - Does not account for long term effects of MC
  - Need to consider the population of uncircumcised men and program coverage
  - Need simulation modeling

Number of circumcisions to prevent one incident HIV infection over 10 years with 75% program coverage

Efficacy (1-IRR)	No. Surgeries per infection averted over 10 years
0.7	31
0.5	46
0.4	58

Cost per infection averted over 10 years with 75% program coverage
Research Cost $69 per surgery

Efficacy	Cost per infection averted ($)
0.7	2125
0.5	3136
0.4	3911

Cost per infection averted over 10 years with 75% program coverage
Cost $35 per surgery

Efficacy	Cost per infection averted ($)
0.7	1078
0.5	1591
0.4	1984

Caveats

- Costs per infection averted are high in Rakai due to relatively low incidence and prevalence in this rural setting
  - HIV prevalence = 11%
  - HIV incidence = 1.3/100 py in uncircumcised men
- Costs per infection averted will be lower if
  - HIV prevalence and incidence are high (e.g., SA)
  - Few men are circumcised, and program coverage is high
  - Efficacy of circumcision ~ 0.5
  - Longer time projection (e.g., 20 years)

Limitations and strengths

- Limitations:
  - This model is specific to Rakai epidemiologic parameters
  - Trial data on MC efficacy is only known over 2 years. Efficacy may increase with longer time from surgery (Rakai trial efficacy in 2nd year of follow up was 75%)
- Strengths:
  - Model is based on empirical data with minimum assumptions
  - Model output closely approximates to observed rates (e.g. HIV incidence), suggesting the model accurately reflects the Rakai epidemiology
  - Efficacy based on trial outcomes

Modeling Conclusions

- Male circumcision may potentially:
  - Reduce HIV incidence in the population
  - Reduce R_o ~1
  - The number of surgeries per HIV infection averted is likely to be cost-effective,
- The effects of MC will depend on:
  - Efficacy in males
  - Effects in females unknown (depends on trial results)
  - Duration of efficacy
  - HIV incidence
  - Prevalence of MC
  - Program coverage of uncircumcised males
  - Prevention of disinhibition

Follow-up Study, Post trial Surveillance in Rakai (*NIH*)

- Provide circumcision surgery to uncircumcised, control participants
- Continue with analysis to demonstrate efficacy of circumcision to prevent STIs
- Conduct long-term, post trial surveillance to assess effectiveness of circumcision, monitor sexual risk behaviors and estimate number of surgeries required to avert one HIV infection over five years

Museveni Cautions Youth on Circumcision

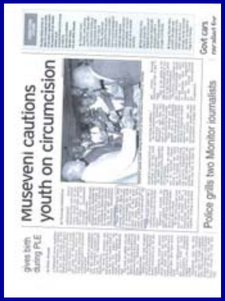

The advise

- “Some NGOs have been saying rubbish about circumcision but I will continue encouraging the youth to abstain and use condoms only if they must. How many Bagisu have died of AIDS and yet all of them are circumcised” he asked.

Thank you

Acknowledgements:

Ron Gray
Maria Wawer
David Serwadda
Nelson Sewankambo
Fred Wabwire-Mangen
GodfreyKigozi
Steven Watya
Fred Makumbi
Fred Nalugoda
Steve Reynolds
Rakai Health Sciences Program Study Teams

Nyanza (Kenya) Modelling Approaches

Nico Nagelkerke[1,2]
Robert Bailey[3]
Steve Moses[2]

1. Dept Community Medicine, United Arab Emirates University, Al Ain, UAE
2. Dept of Medical Microbiology, University of Manitoba, Winnipeg, Canada
3. Division of Epidemiology, University of Illinois at Chicago, Chicago, USA

Nyanza Province (Kenya)

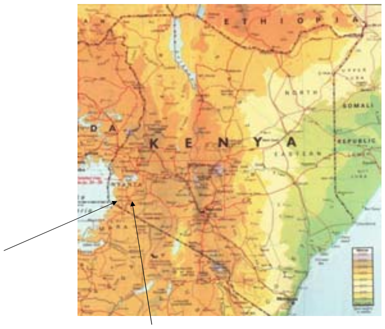

Nyanza Province

- The provincial capital is Kisumu, the third largest city in Kenya.
- The province has a population of 4,392,196 (as of 1999) within an area of 16,162 km^2 . Largely agricultural
- Population is
  - 3 million Nilotic (not Bantu), their native language is Luo (Kiswahili and English learnt in school).
  - 1.5 million Kisii (Gusii), bantus
- Male circumcision is not widely practiced among Luos (unlike most other ethic groups in Kenya)

HIV prevalence over time

- Has the highest HIV prevalence of all Kenyan provinces
  - Variable estimates, all highest in Kenya
  - 20% in men, 30% women (Buvé, AIDS 2001)
  - Women 15-49:18.3%; Men 15-49 11.6%; Kenya 8.7%, and 4.7% (DHS 2003)
- Kisumu (Luo) pregnant women
  - 19.6% (1993)-29.5% (1998; WHO/UNAIDS)

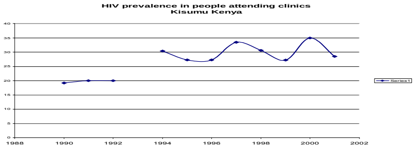

Valley of Life or Death

- High rates of HIV in Luo (Nyanza) compared to neighbouring Luya (Western) was subject of BBC documentary “The valley of Life or Death” (Nov. 2000) which made the circumcision issue widely known
  - *So here in this valley, although the two groups should have the same risk, the people on one side have a 20% HIV infection rate and the people on the other 7%.*

HIV in Nyanza

- In addition to non-circumcision there are other Luo habits that are risk factors for HIV transmission
  - “Cleansing” of widows
  - When a man dies, one of his brothers or other male relatives is required to step in
  - Unsafe sex between FSW and clients common
    - As elsewhere in Kenya

Clinical Trial on MC in Nyanza

RC Bailey et al; *Lancet*. 2007.

- Now 3 trials (Auvert, Gray, Bailey) have shown 50-60% reduction in HIV infection risk after male circumcision
- In Kisumu 22/1391 in intervention *vs* 47/1393 in control group seroconverted
  - 53%-60% risk reduction rates
    - Men 18-24 years old
    - Baseline screening 537/6159 (8.7%) positive/indeterminate
    - Incidence in control group approx. 2%/year
    - 21 adverse events resolved quickly
    - No behavioral risk compensation (disinhibition) observed

Modelling

- Two approaches
  - Random (proportionate) mixing model
  - Compartmental Model
- *BMC Infectious Diseases* 2007, **7:**16

Random Mixing Model

- Only equilibrium ("statics") explored,60% reduction in risk. MC from 0 to 100%
- Used 2-gender Anderson-May model; extended Dushoff's work to calculate equilibrium prevalences
- Distribution N(x) of x (scaled) mixing rates x = βcD , moments N^1, N^2, Of these I(x) infected
- Equilibrium : $I_m(x) = x\Lambda^f(N_m(x)-I_m(x))$
- $R_0^2 = N_m^2 N_f^2 / N_m^1 N_f^1$
- Gamma distributed mixing rates assumed (long tails)
  - Different levels of heterogeneity
- Mathematica used to do calculations

$$I^m(x) = \frac{x\Lambda^f N^m(x)}{1 + x\Lambda^f} \qquad \Lambda^m = \frac{\int_0^\infty x I^m(x)\,dx}{\int_0^\infty x N^m(x)\,dx}$$

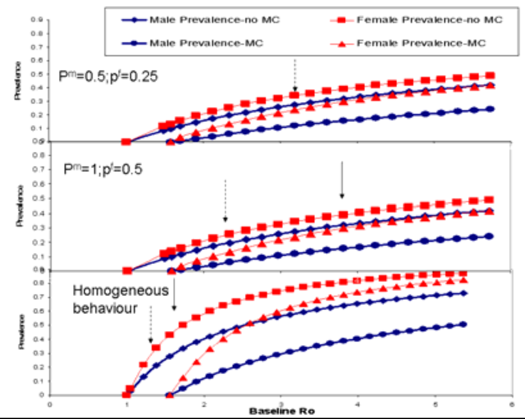

Compartmental Model

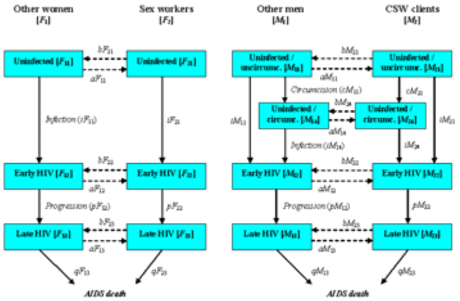

Details

- MC introduced in 2010, but 10% pre-intervention level of MC assumed
  - i.e. includes only Luo
- Rates of circumcision set to obtain specific levels (50%, 80%)
- Parameters gleaned from literature + fitting

Sensitivity Analyses

1. Base RR =0.40;
2. Sensitivity studies:
   - RR = 0.25, 0.60
   - Disinhibition (risk compensation): no condom use during FSW-clients contacts
   - Effect of circumcision on M-F transmission (25% Risk Reduction). But evidence for this effect is still poor

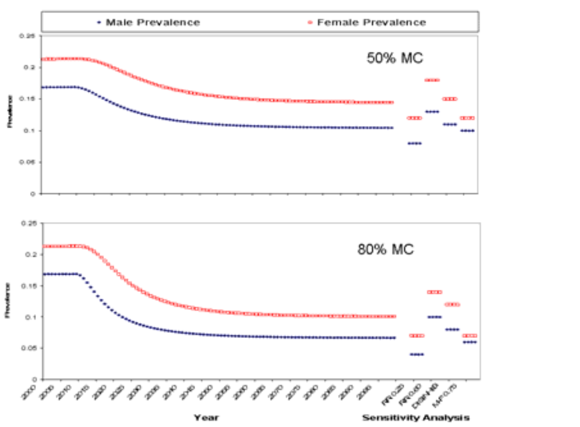

Cost-effectiveness

- MC can increase life expectancy in high prevalence countries by 5-10 years
- When fully operational will avert approximately 10,000 (50% uptake)-15,000 (80% uptake) HIV infections annually
  - But depends on time of MC (pre-puberty is best)
- Programme in equilibrium would require approximately 30.000-45000 (2-3% of 1.5 million men) MC/year
- If costs/MC $50-100, then costs per HIV infection averted $150-400.
  - Per capita costs +/- 1 $/year
  - Per life-year saved $7-20 (assuming conservatively 20 extra years)
- Cost effectiveness much higher than ART
  - US$984 (95% CI 913-1,078) per life year gained in RSA (Cleary et al, 2006); 100 times less cost-effective!

Acceptability in Nyanza

- Will male circumcision be an acceptable intervention?
  - Considered (culturally) acceptable (Bailey, AIDS Care 2002)
  - But also meets with resistance. Quotes from “The Standard” 11-08-2007
    - Luo Council of Elders will have none of it: *Promoting male circumcision as a tool to prevent HIV/Aids infections*, says Riaga, *will fuel prostitution, as people will indulge in careless sex.*
    - KMA chairman Dr Stephen Ochiel says, "*Attitude change should be the best recommendation. Some men believe circumcision gives them a leeway to have multiple partners*," says Ochiel.

Discussion

- Male circumcision with high coverage may lead to > 50% reduction in HIV prevalence
  - Highly cost-effective
- May take 1-2 decades to take full effect
  - But faster if “catch-up” campaigns organized
- Effect larger in men than in women
- Assumed fixed reduction in risk per contact
  - Heterogeneity in susceptibility will lead to higher reduction in *conditional* risk, and thus….
  - Those least susceptible will benefit relatively most (cf. Rakai study). Supported by empirical data:
    - Rakai: 0 of 50 circumcised men with infected spouses became HIV-infected after nearly 2 years of follow-up, whereas 40 of 137 of uncircumcised seroconverted

USAID FROM THE AMERICAN PEOPLE | HEALTH POLICY INITIATIVE

Cost of Male Circumcision and Implications for Cost-Effectiveness of Circumcision as an HIV Intervention

Study Team*: Lori Bollinger[2], Steven Forsythe[2], Bafana Khumalo[5], Gayle Martin[1]; Rejoice Nkambule[4], Tanvi Pandit-Rajani[1], Dean Peacock[5], Tshehlo Relebohile[3], John Stover[2]

June, 2007; Updated November 2007

[1] Constella Futures
[2] Futures Institute
[3] Lesotho Ministry of Health and Social Welfare
[4] Swaziland Ministry of Health and Social Welfare
[5] Sonke Gender Justice

*alphabetical order

USAID FROM THE AMERICAN PEOPLE | HEALTH POLICY INITIATIVE

Study Components

1. Understanding the social, cultural and policy context
2. Costing Male Circumcision
3. Modeling the Impact

USAID FROM THE AMERICAN PEOPLE | HEALTH POLICY INITIATIVE

What the study will not tell you …

- Whether circumcision should be implemented in the selected countries …
- How circumcision should be implemented in the selected countries …
- What the clinical guidelines should be …
- Who should perform male circumcision …

The study is intended to provide some information (alongside other considerations) that will feed into country-led policy and planning processes

USAID FROM THE AMERICAN PEOPLE | HEALTH POLICY INITIATIVE

Methodology

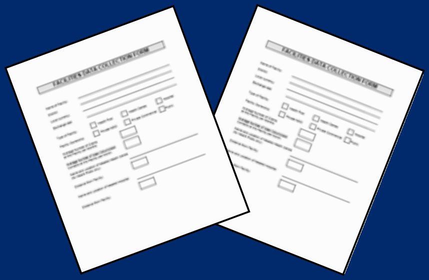

USAID FROM THE AMERICAN PEOPLE | HEALTH POLICY INITIATIVE

Key considerations in design

- Study focuses on adult circumcision only
- From the perspective of the health facility
- Cost data collected on how circumcision currently implemented
- Consideration also given to services not currently part of circumcision (counseling with or without testing, training, communications)
- 3 countries
  - Allows for standardization, comparison and validation
  - Countries: high HIV prevalence; moderate to low MC prevalence (Williams et al 2006)

USAID FROM THE AMERICAN PEOPLE | HEALTH POLICY INITIATIVE

Key considerations in design

- Variation in circumcision practices and patient management made explicit and costed accordingly
- Several provider types considered and levels of health facilities
  - Public and NGO/FBO
  - District hospital, referral hospital and clinic
- Ingredients approach to costing
  - Allows for flexibility in use of the cost analysis
  - Enables specific aspects of intervention to be added or subtracted to inform planning

USAID FROM THE AMERICAN PEOPLE | HEALTH POLICY INITIATIVE

Cost Estimation

- Direct and indirect costs per person circumcised at each provider (j) type:

$$C_j = c_j^{direct} + S * c_j^{indirect}$$

$$c_j^{direct} = \sum_{k,j} c_{k,j}$$

$$c_j^{indirect} = \sum_{l,j} c_{l,j}$$

where:

$c_{k,i}$ = direct cost per person served at provider, j

k = staff, drugs, supplies etc.

$c_{l,i}$ = indirect cost per person served at provider, j

l = facility equipment, facility utilities, facility supervision etc.

S=share of total health facility operation that that MC services account for

USAID FROM THE AMERICAN PEOPLE | HEALTH POLICY INITIATIVE

Cost Estimation

- Direct and indirect costs of MC with complications were also estimated
- Unit cost is weighted by the cost of MC complications and the probability of each complication occurring

$$C_j^{*} = pr(uncomplicated\ MC) * C_j^{uncomplicated} + pr(complication) * C_j^{complication}$$

USAID FROM THE AMERICAN PEOPLE | HEALTH POLICY INITIATIVE

Cost Analysis

- Cost of providing circumcision at provider, j, in year, y:

$$TC_{j,y} = C_j * Q_{j,y}$$

- Costs are discounted and inflation adjusted:

$$TC = \sum_{j,y} \left[TC_{j,y} \big/ (1+r)^a \right]$$

r = real discount rate

a = # years into the future

USAID FROM THE AMERICAN PEOPLE | HEALTH POLICY INITIATIVE

Data Collection

- Site selection:
  - 4 sites per country
  - Combination of rural and urban facilities
  - Hospitals (referral and district), clinics

USAID FROM THE AMERICAN PEOPLE | HEALTH POLICY INITIATIVE

Preliminary Results: Cost Analysis

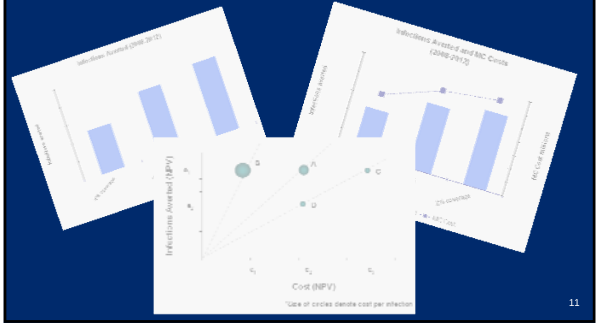

Facility	1	2	3	4	5	6	7	8	9	10	11
**Visit #1 (initial visit, examination, booking)**											
Doctor (minutes)											
Nurse (minutes)											
Counselor (min)											
**Visit #2 (surgical procedure)**											
Doctor (minutes)											
Nurse (minutes)											
Nurse Ast (minut)											
**Visit #3 (post-surgery follow-up; +2-3 days)**											
Doctor (minutes)											
Nurse (minutes)											
**Visit #4 (follow-up; +7 days)**											
Nurse (minutes)											

Facility	1	2	3	4	5	6	7	8	9	10	11
**Visit #1 (initial visit, examination, booking)**											
Doctor (minutes)											
Nurse (minutes)											
Counselor (min)											
**Visit #2 (surgical procedure)**											
Doctor (minutes)											
Nurse (minutes)											
Nurse Ast (minut)											
**Visit #3 (post-surgery follow-up; +2-3 days)**											
Doctor (minutes)											
Nurse (minutes)											
**Visit #4 (follow-up; +7 days)**											
Nurse (minutes)											

Facility	1	2	3	4	5	6	7	8	9	10	11
**Visit #1 (initial visit, examination, booking)**											
Doctor (minutes)											
Nurse (minutes)											
Counselor (min)											
**Visit #2 (surgical procedure)**											
Doctor (minutes)											
Nurse (minutes)											
Nurse Ast (min)											
Anesthesia	Local	Local	Local	Local	Local	Local	Local	Local	Local	Local	Local
Antibiotics	None	None	None	None	Penicillin	Penicillin	None	None	None	None	None
Analgesics	Paracetamol	Paracetamol	Paracetamol	Paracetamol	Ibuprophen	Ibuprophen	Ibuprophen	Ibuprophen	Paracetamol	Paracetamol	Paracetamol
**Visit #3 (post-surgery follow-up; +2-3 days)**											
Doctor (minutes)											
Nurse (minutes)											
Dressing	No	Yes	No	Yes	Yes	Yes	No	No	No	No	No
**Visit #4 (follow-up; +7 days)**											
Nurse (minutes)											

USAID FROM THE AMERICAN PEOPLE | HEALTH POLICY INITIATIVE

Variation in Direct Costs

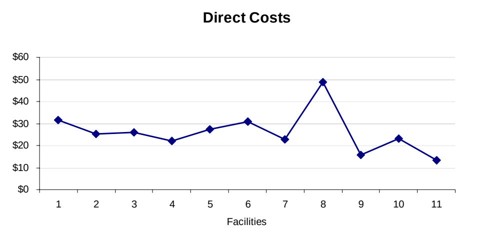

USAID FROM THE AMERICAN PEOPLE | HEALTH POLICY INITIATIVE

Variation in Indirect Costs

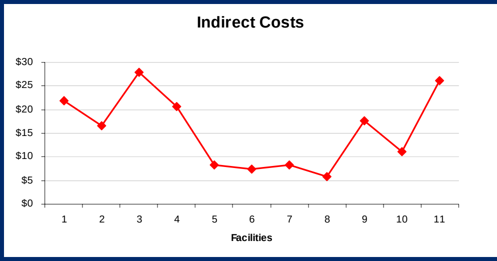

USAID FROM THE AMERICAN PEOPLE | HEALTH POLICY INITIATIVE

Cost of Comprehensive MC Services

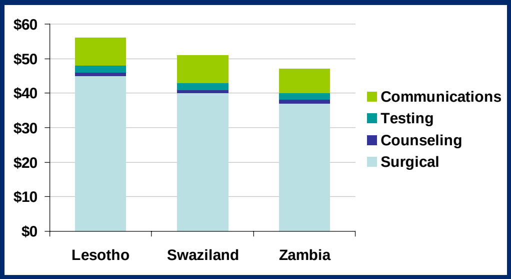

USAID FROM THE AMERICAN PEOPLE | HEALTH POLICY INITIATIVE

Factors that explain differences in Unit Costs

- General convergence in surgical practice in non-private providers
- Small variation:
  - whether or not antibiotics are routinely prescribed
  - whether or not dressings are reapplied at the post-operative visit
- Commodity costs not an important source of variation
- Indirect costs are an important driver of cost differences:
  - level of facility (size, complexity of services available etc.)

USAID FROM THE AMERICAN PEOPLE | HEALTH POLICY INITIATIVE

Preliminary Results: Implications for Cost Effectiveness Analysis

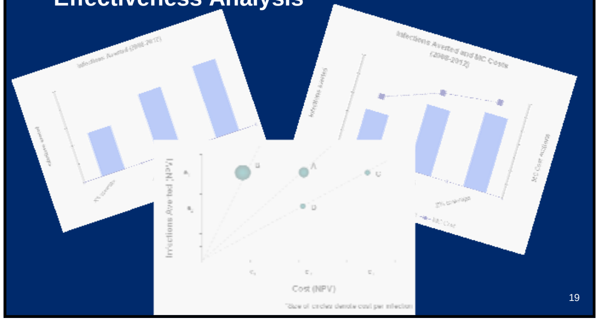

19

USAID FROM THE AMERICAN PEOPLE | HEALTH POLICY INITIATIVE

Impact Projections Made with Spectrum

- Simulation module with four risk groups (none, low, medium, high) by sex
- Model fitted to historical prevalence trends
- Two projection scenarios
  - No change in MC prevalence
  - Target MC prevalence achieved by 2015
    - Lesotho: 0% to 50%
    - Swaziland: 15% to 58%
    - Zambia: 17% to 57%
- 60% reduction in female to male transmission

20

USAID FROM THE AMERICAN PEOPLE | HEALTH POLICY INITIATIVE

Scaling up MC (2008-2020)

		Lesotho	Swaziland	Zambia
MC Target for 2015		53%	58%	58%
MCs per infection averted	2008-2020	6.1	4.1	8.0
Cost per infection averted*	2008-2020	$292	$176	$313

*Real discount rate = 3%

21

USAID FROM THE AMERICAN PEOPLE | HEALTH POLICY INITIATIVE

Summary of Cost-Effectiveness

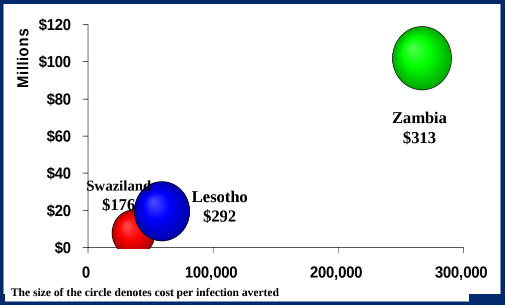

22

USAID FROM THE AMERICAN PEOPLE | HEALTH POLICY INITIATIVE

Health service implications (Average for 2008-2020)

	Lesotho	Swaziland	Zambia
# MC per month	2289	941	14,014
# MC per week	572	235	3234
# MC per day	114	47	461
Average annual cost of MC*	$1.5 million	$0.6 million	$7.9 million
Cumulative cost of MC (2008-2020)*	$20.0 Million	$7.8 Million	$102 million

*Real discount rate = 3%

23

USAID FROM THE AMERICAN PEOPLE | HEALTH POLICY INITIATIVE

What is the impact of the pace of scale-up of circumcision?

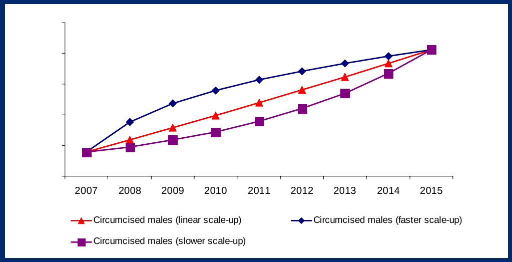

24

What is the impact of the pace of scale-up of circumcision?

Swaziland

	Linear scale-up	Slower scale-up	Faster scale-up
	2008-2015	% difference with linear scale-up	
# MCs	**332,639**	-6%	+9%
# Infections averted	**43,782**	-18%	+31%
Cost per IA*	$437	+15% ($480)	-16% ($350)

*Real discount rate = 3%

25

Conclusion

- Level in the health service where MC is provided matters
- Pace of scaling up matters
- MC can be a cost effective intervention
- Benefits accrue over time
- Scaling up MC is not without challenges …
  - Indirect costs should not be underestimated
  - Health service, financial and human resource implications are significant but not insurmountable
  - Innovative ways have to be found to involve all providers (including the private sector and NGOs)

26

Decision-Maker's Tool

John Stover and Lori Bollinger

Prepared for:
UNAIDS/WHO/SACEMA Consultation
Making Decisions on Male Circumcision for HIV Risk Reduction: Modelling the Impact and Costs

Stellenbosch, South Africa – 15-16 November 2007

Futures INSTITUTE

Purpose

- Allow decision-makers to understand the cost and impact of scaling-up MC services
- Understand the effects of:
  - Service delivery approach
  - Target populations
  - Pace of scale-up

Futures INSTITUTE

Decision-makers Tool: Costing portion

- Based on LSZ costing work
  - Field-tested / applied in 3 countries
  - Similar to CostIt model (WHO-CHOICE)
- Three components:
  - 1. Questionnaire
  - 2. Costing template (Excel)
  - 3. Policy screen (Excel)

Futures INSTITUTE

1. Questionnaire

- Facility section (facility id, # clients)
- Staffing, equipment, maintenance
  - Including transport & utility costs
- Procedure-related (includes staff, drugs, supplies, lab costs)
  - Circumcision procedure, including counseling/testing
  - Post-circumcision care – normal
  - Post-circumcision care – complications (infection, excessive bleeding, excessive pain, other)

Futures INSTITUTE

2. Costing template (Excel)

- Contains default assumptions on prices and quantities
  - Input cost worksheets are separate from quantity worksheets
  - Input cost data available at national level
  - Amortization included for capital costs
- Costs for following WHO/UNAIDS MC guidelines are imputed and included
  - HIV testing & counselling
  - IEC campaign

Futures INSTITUTE

3. Policy screen (Excel)

- % MCs provided by different service delivery modes
  - Public hospital/clinic, NGO hospital, private provider, mobile clinic
- User fee amount (default = $0) can be filled in by delivery mode

Futures INSTITUTE

Representative unit costs by service delivery mode

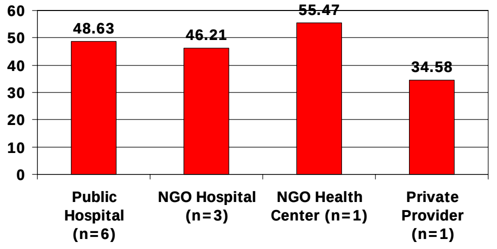

Futures INSTITUTE

Outstanding issues

- Current guidelines are incorporated into workbook – should they be made policy variables?
  - Provides focus on 'other' elements
- Any other policy variables that should be included?
- Other issues?

Futures INSTITUTE

Impact Model - Aggregate

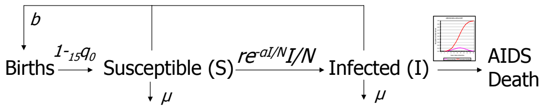

N = S+I

Inputs: S_0, b, ${}_{15}q_0$, μ, progression to AIDS death

Futures INSTITUTE

Fitting the Model

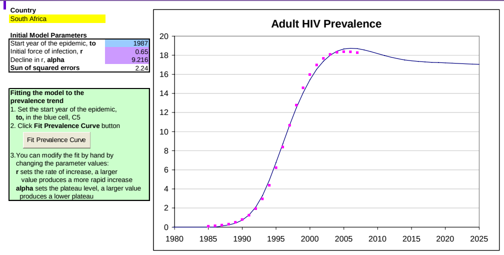

Futures INSTITUTE

Fitting the Model

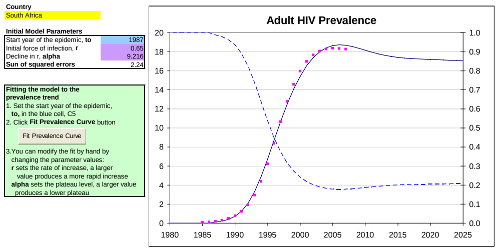

Futures INSTITUTE

Influence of MC

MC Inputs: reduction in transmission due to MC
$(1 - r_{MC} / r_{NoMC})$

Fit parameters: α, r'

Since $r' = r_{NoMC} MC\%_0 + r_{MC}(1-MC\%_0)$

Calculate: r_{NoMC} and r_{MC}

$r_t = r_{NoMC} MC\%_t + r_{MC}(1-MC\%_t)$

Futures INSTITUTE

Impact Model – Age and Sex

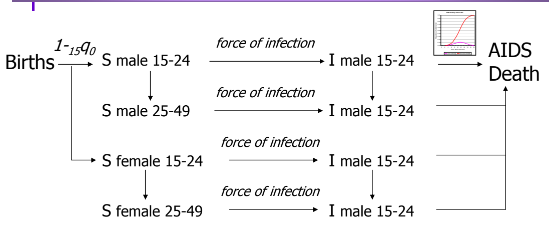

Force of infection by age and sex is a function of $r_{a,s,c}$, average risk, proportion of contacts with 15-24 and 25-49 of opposite sex and HIV prevalence in opposite sex.

Futures INSTITUTE

Extra Inputs Required for Age and Sex Model

- μ, non-AIDS mortality by age and sex
- Initial population by age and sex
- Prevalence by age and sex over time
  - Generally only available for one year
- Sexual mixing matrix

Age/Sex	Female 15-24	Female 25-49	Total
Male 15-24	0.09	0.20	0.29
Male 25-49	0.23	0.48	0.71
Total	0.32	0.68	1.00

Futures INSTITUTE

Fitting the Age/Sex Model

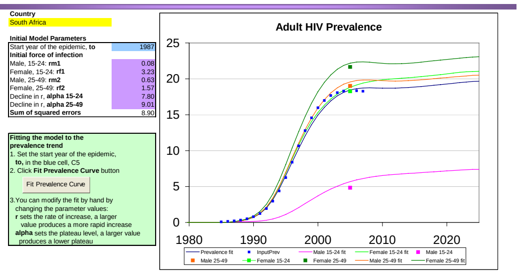

Futures INSTITUTE

Interactive Decision Screen

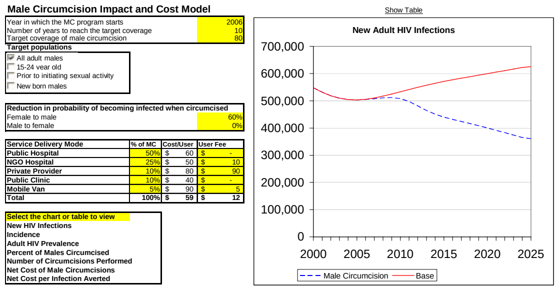

Target = 80% Adult Males by 2015

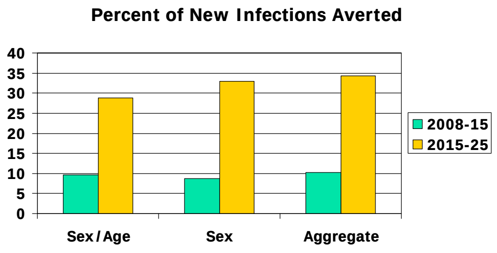

Futures INSTITUTE

Target = 80% 15-24 Males by 2015

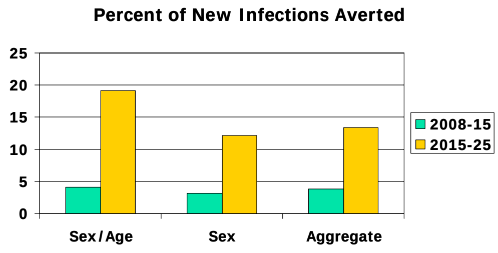

Futures INSTITUTE

Issues for Discussion

- Which model?
  - Aggregate model is easier to set up
    - But we could set up age/sex model for all relevant countries
  - Age/sex model may not be more accurate if data are not available
- Do we need additional population targeting and service delivery options?

Futures INSTITUTE

Male circumcision for HIV prevention: who, what, when?

UNAIDS/ WHO/ SACEMA Consultation, Stellenbosch, 2007

Richard White
and colleagues

Background

- Male circumcision important in explaining the heterogeneous spread of HIV in Africa
- MC reduces HIV acquisition in men by ~60%
- UNAIDS recommends MC should be scaled up in countries with high prevalence heterosexual HIV epidemics with priority given to young HIV- males and high risk groups
- MC is not currently recommended for HIV+ males because of concerns over ↑ risks to F if sex resumed before wound healing
- **Questions**
  - What is population level impact?
  - How does this vary by age, sex, coverage and time to scale-up?
  - How much risk compensation would negate impact?
  - Impact of (inadvertently?) circumcising HIV-infected males?
    - Impact if circ lowers M>F HIV trans. prob.?

Aim and objectives

- Explore the population level impact of various scenarios of the rollout of MC in an urban population in East Africa
  - Estimate the population level impact of a linear increase in the proportion of circumcised HIV-uninfected males in the targeted age groups
    - from 25% (pre-intervention prevalence) to 75% over 5 years,
    - with no risk compensation
  - Estimate impact of varying the coverage and time to scale-up
  - Estimate magnitude of risk compensation required to negate impact of the plausible MC intervention, and the potential of increased condom use(due to counselling) to increase impact
  - Estimate population level impact of inadvertently circumcising HIV infected males

Methods and assumptions (1)

- Individual-level stochastic model *STDSIM*
  - simulates the spread of HIV and STIs
  - dynamic sexual network of simulated individuals and their sexual contacts
  - Present average of 500 iterations per scenario
- Baseline scenario
  - Fitted to the demographic, behavioural and epidemiological characteristics of the urban population (Kisumu) in East Africa in 1997 and 2006, and the impact of MC measured in young men in the Kisumu RCT (59%, 95% CI=30-76)
  - 25% of males already circumcised

Circumcision assumptions

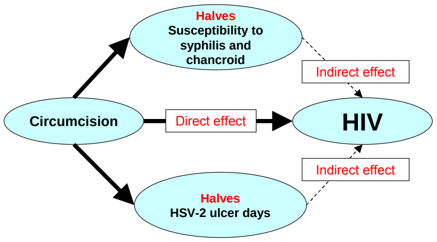

HIV- M: ► reduced susceptibility to HIV acquisition (fitted to RCT)
► double the risk of acquisition from HIV+ F over 6m in 15% M who resume sex before healing
HIV+ M: ► double risk of transmission to F over 6m, in 15% of men who resume sex before healing (only direct effect on M to F)

Methods and assumptions (2)

- Intervention scenarios
  - Intervention starts 1st January 2007
- Default scenario
  - Linear increase in proportion of HIV uninfected males in targeted age group from 25% to 75% over 5 years
  - no risk compensation
  - Target age groups: 15-24y, 25-34y, 35-49y, 15-49y, 15+y, All-ages, neonates and 13 year olds
- Outcomes
  - Impact on HIV incidence (%) :
    = (1 - mean IRR among MF 15-49 years old) * 100
  - Number of HIV infections averted(MF 15-49y)/ 1000 circumcisions in males and females of all ages
  - Over 2,5,10,20,30,40 and 50 years

Methods and assumptions (3)

- Alternative scenarios
  - Coverage of HIV negatives varied between 25% (no increase) and 100%
  - Scale-up period varied between 0 and 20 years
  - Behaviour change
    - In default scenario condoms are used in 40% of casual and sex-contacts after 2000, with 10% failure
    - Vary condom use between 0-30% (risk compensation) and 50-80% (effective safe-sex counselling) in
      - Recently circumcised men
      - All circumcised men (including 25% already circumcised)
  - Direct effect on M to F HIV transmission probability
    - Assume 50% reduction

Methods and assumptions (4)

- Alternative scenarios continued
  - Effect of circumcising HIV infected males
    - Circumcise 15-49 year old males regardless of HIV status
      - With and without assuming circumcising males halves the male-to-female transmission probability of HIV
    - Keep number of circumcisions over each time period equal to that modelled in default scenario

Results: Baseline scenario

Population structure by age and sex

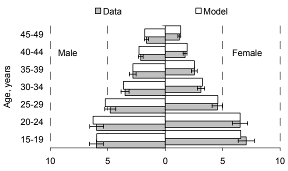

Prevalence of steady partnerships

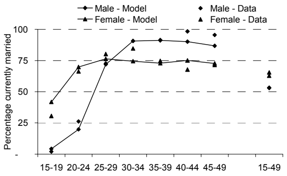

Results: Baseline scenario

STI prevalence

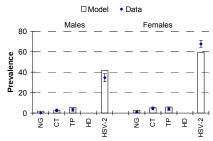

HIV prevalence trend

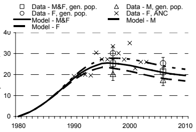

HIV prevalence by age - Males

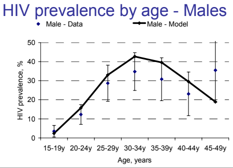

HIV prevalence by age - Females

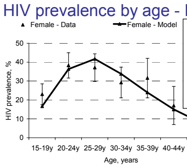

Age difference in casual and steady partnerships:
Data: 5 years
Model: 6.6 years

Results: Fitted RCT impact in young men

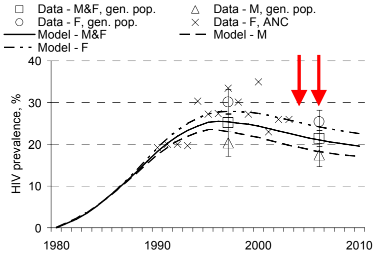

Intention-to-treat impact* (1-IRR) %:

Model	Data
59(30-76)	59(30-76)

Explained by:

Direct ~ 96%
HSV-2 ulcer ~ 4%
TP/HD acq. ~ 0%

* Excluding 4 individuals subsequently known to be HIV+ at baseline. Bailey, R. C., et al (2007). Lancet 369(9562): 643-56.

Population level impact: Targeting by age (1 - mean IRR over period) * 100 (15-49 year olds, %)

Males

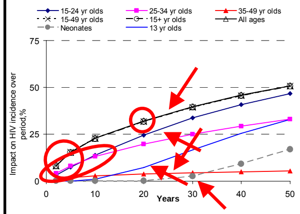

Females

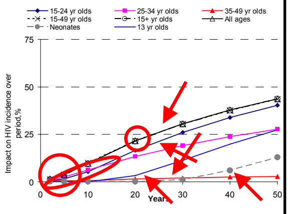

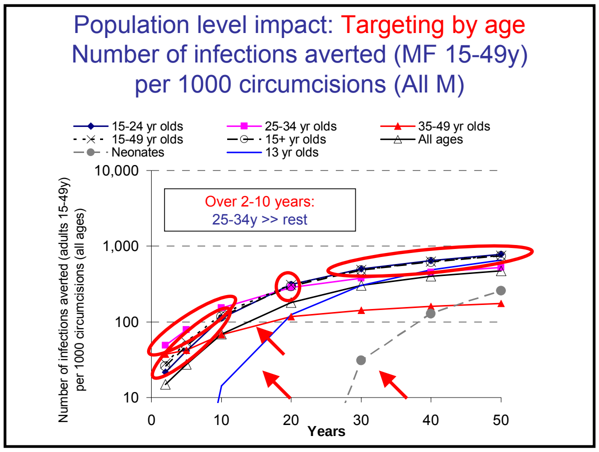
Population level impact: Targeting by age
Number of infections averted (MF 15-49y)
per 1000 circumcisions (All M)
15-24 yr olds
25-34 yr olds
35-49 yr olds
15-49 yr olds
15+ yr olds
All ages
Neonates
13 yr olds
Over 2-10 years:
25-34y >> rest
Number of infections averted (adults 15-49y)
per 1000 circumcisions (all ages)
10,000
1,000
100
10
0
10
20
30
40
50
Years

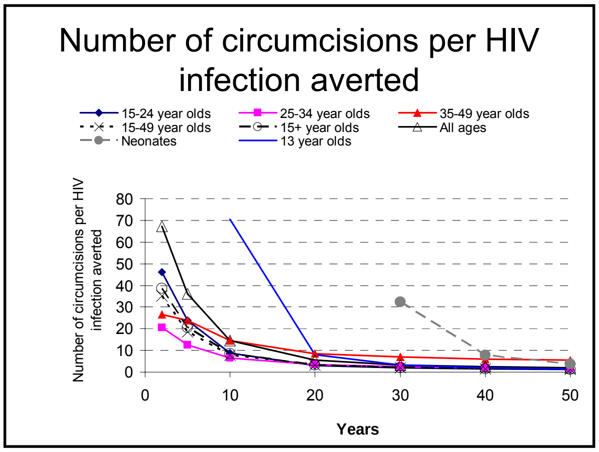
Number of circumcisions per HIV infection averted
15-24 year olds
25-34 year olds
35-49 year olds
15-49 year olds
15+ year olds
All ages
Neonates
13 year olds
Number of circumcisions per HIV infection averted
80
70
60
50
40
30
20
10
0
0
10
20
30
40
50
Years

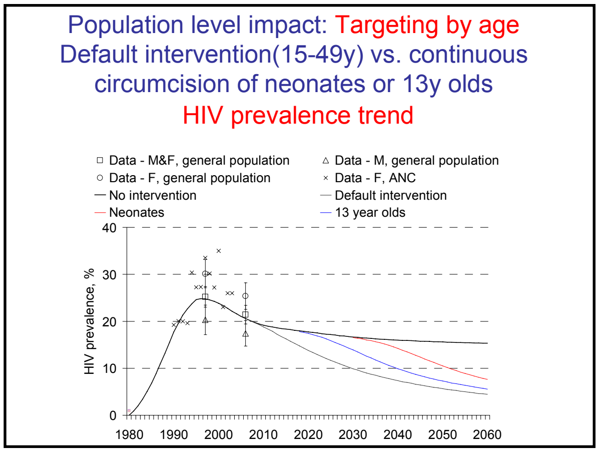
Population level impact: Targeting by age
Default intervention(15-49y) vs. continuous circumcision of neonates or 13y olds
HIV prevalence trend
Data - M&F, general population
Data - M, general population
Data - F, general population
Data - F, ANC
No intervention
Default intervention
Neonates
13 year olds
HIV prevalence, %
40
30
20
10
0
1980
1990
2000
2010
2020
2030
2040
2050
2060

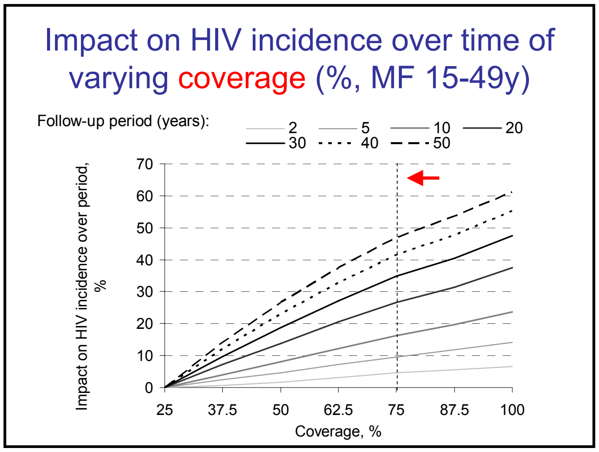
Impact on HIV incidence over time of varying coverage (%, MF 15-49y)
Follow-up period (years):
2
5
10
20
30
40
50
Impact on HIV incidence over period, %
70
60
50
40
30
20
10
0
25
37.5
50
62.5
75
87.5
100
Coverage, %

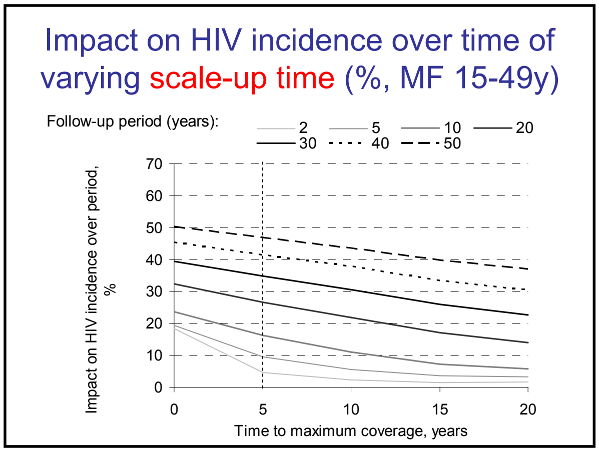
Impact on HIV incidence over time of varying scale-up time (%, MF 15-49y)
Follow-up period (years):
2
5
10
20
30
40
50
Impact on HIV incidence over period, %
70
60
50
40
30
20
10
0
0
5
10
15
20
Time to maximum coverage, years

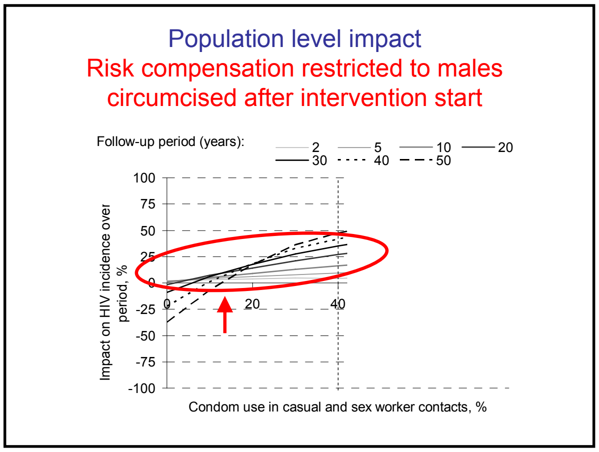
Population level impact
Risk compensation restricted to males circumcised after intervention start
Follow-up period (years):
2
5
10
20
30
40
50
Impact on HIV incidence over period, %
100
75
50
25
0
-25
-50
-75
-100
0
20
40
Condom use in casual and sex worker contacts, %

Population level impact
Risk compensation in all circumcised males

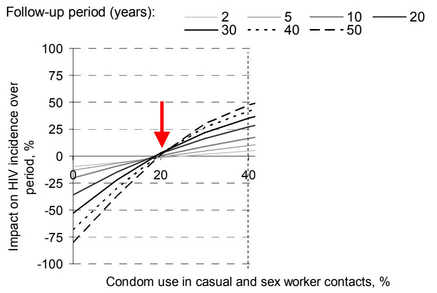

Population level impact in males
of circumcising HIV infected males (15-49y)

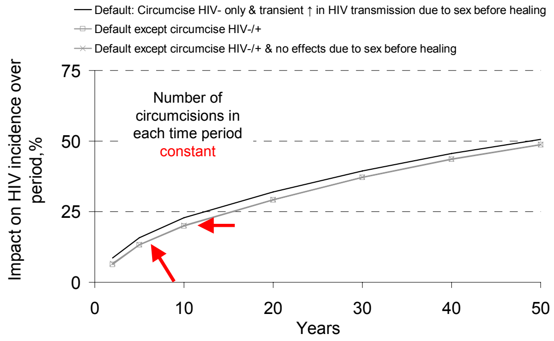

Population level impact in females
of circumcising HIV infected males (15-49y)

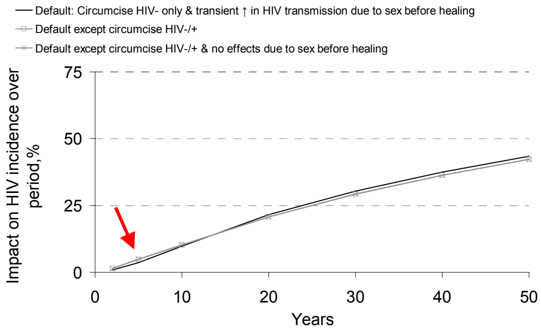

Population level impact in males
of assuming circumcision halves the per-contact
male-to-female HIV transmission probability

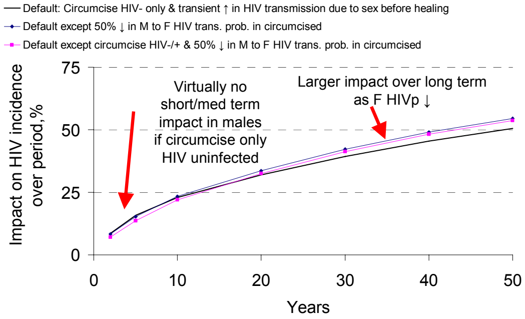

Population level impact in females
of assuming circumcision halves the per-contact
male-to-female HIV transmission probability

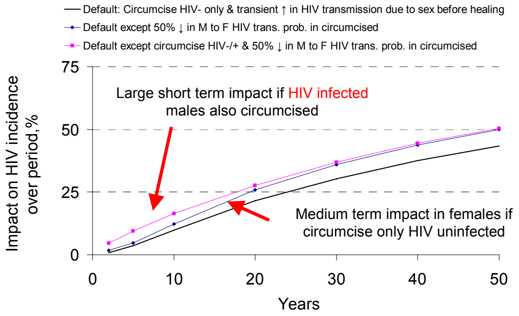

Limitations

- May have overestimated impact
  - Have assumed impact of MC on ulcers
- May have underestimated impact
  - Over longer term because of increasing risk in effective intervention scenarios
- Plausible ranges not shown
- Sensitivity analysis
  - Effect of alternative assumptions of MC on STIs
  - Other key parameter values
- Other scenarios and settings
  - Lower/higher risk behaviours/ STI/ HIV rates

Summary and policy implications

- Short term impact in women will be small unless strong direct impact on M>F transmission *and* circumcision of HIV+s
- Difference M vs F will reduce over time
- Over first 10 years, targeting 25-34 year olds may be more effective per-circumcision than targeting younger males
  - => Change recommended priority age group to 15-34?
- Will wait 20-40 years to get population level impact via neonates

Summary and policy implications

- To maximise the number of infections averted, scale-up quickly with high coverage, while maintaining quality(!)
- Risk compensation
  - If limited to newly circumcised, it will reduce impact over medium term & potential to negate impact over long term
  - Effect is strongly dependent on whether already-circumcised change behaviour
    - => target IEC at already-circumcised?

Summary and policy implications

- Circumcising HIV+ males
  - Will reduce population level impact in males in short term *if* coverage of HIV-uninfected males not maintained
  - Population level impact in females
    - may be small *if* only small proportion of men resume sex before wound healing and circumcision lowers STI rates
    - *or* may increase markedly *if* direct impact on M>F HIV trans. prob.

Acknowledgements

London School of Hygiene and Tropical Medicine
Judith Glynn, Helen Weiss, Kate Orroth, Esther Freeman, Richard Hayes

Institute of Tropical Medicine, Antwerp
Anne Buvé

Erasmus Medical Center, Rotterdam
Roel Bakker

UCSF, US
Craig Cohen

Members of the Study Group on Heterogeneity of HIV Epidemics in African Cities

Funding
Wellcome Trust

The dynamics of male circumcision as an intervention at the population level: preliminary results

Laith J. Abu-Raddad & Ramzi Alsallaq
Fred Hutchinson Cancer Research Center &
University of Washington
Seattle, Washington, U.S.A

MC and HIV Differential prevalence: The Four-City Study

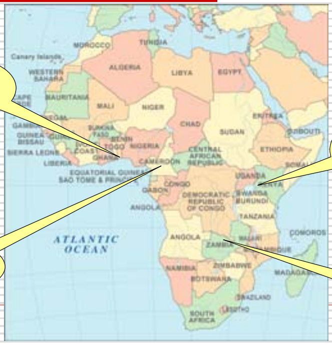

Can male circumcision explain the differential HIV prevalence?

Preliminary results

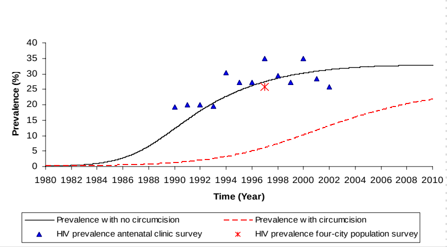

Male circumcision in low prevalence settings?

In any setting with low HIV prevalence but where there is potential for a hyperendemic HIV epidemic, male circumcision is an effective intervention to substantially slow and limit the progression of the epidemic in the population

What does male circumcision do to R_0?

Preliminary results

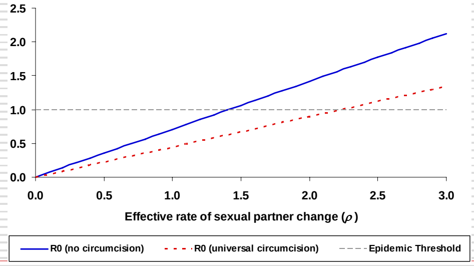

Male circumcision and R_0

Male circumcision increased the epidemic threshold for sustainability (R_0=1) by 57% from ρ = 1.4 to ρ = 2.2 partners per year

HIV epidemiology in a setting with low coverage of male circumcision

Preliminary results

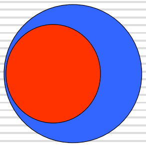

HIV infectious spread is sustainable in the majority of the population

Male circumcision: a “quarantine” measure for HIV infection

Preliminary results

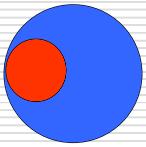

HIV infectious spread is sustainable only in a minority of the population where sexual risk behavior level is high

Epidemiologic evidence from Western Africa

Among the male population of Accra aged 15-59 years, 84% of prevalent cases of HIV were attributable to transactional sex (Cote et al 2004)

Unprotected sex with male clients of female sex workers in Cotonou could account for most if not all of the estimated yearly numbers of HIV infections in Cotonou women (Lowndes et al 2002)

A sexist intervention benefiting only males?

Preliminary results

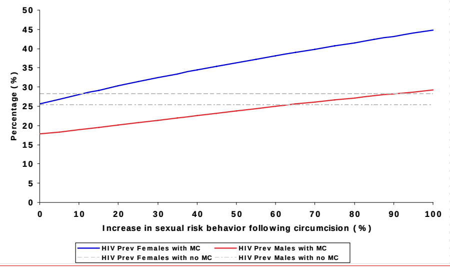

Sensitivity to two assumptions of how risk behavior could increase

Balance of partnerships:

- Increase in risk behavior among men imply increased risk among women
- Differential disease morbidity and mortality of women versus men could create demographic imbalance (HAART could limit this aspect)

MC uptake: Relative decline in incidence rate up to 2020 with slow roll-out

Preliminary results

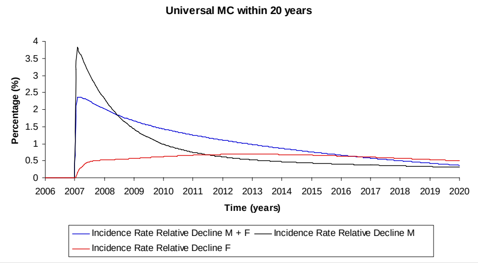

MC uptake: Relative decline in incidence rate up to 2020 with rapid roll-out

Preliminary results

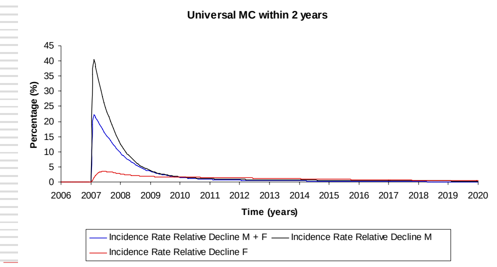

MC uptake: HIV prevalence in 2020 with universal MC but at different rates

Preliminary results

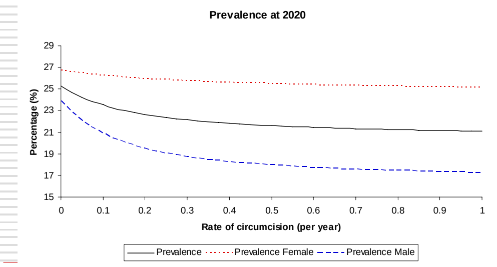

MC uptake: number of MCs per infection averted up to 2020

Preliminary results

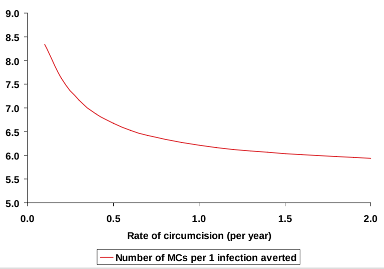

Parameterization of the risk of exposure to HIV in the population

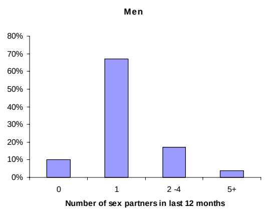

Update of findings from the Rakai trials

Ron Gray

HIV and HSV-2 acquisition in HIV-negative men

Male HIV incidence with completed follow up over 24 months

- The trial was stopped when only 44% of participants could complete the 2nd year follow up
- Updated 2nd year incidence for completed follow up (Nov 2007)

Male HIV Incidence by follow up interval and cumulatively 0-24 months (Nov 2007)

FU Interval	Intervention		Control		IRR (CI)
	Inc/py	Inc/ 100 py	Inc/py	Inc/ 100 py	
0-6	14/1172	1.2	19/1207	1.6	0.76 (0.4-1.6)
6-12	5/1191	0.4	14/1176	1.2	0.35 (0.1-1.0)
12-24	6/2133	0.3	26/2165	1.2	0.23 (0.1-0.6)
0-24	25/4569	0.6	59/4629	1.3	0.43 (0.3-0.7)

HSV-2 acquisition

- 5534 HIV-neg men screened for enrollment HSV-2 using Kalon IgG HSV-2 ELISA
- 3393 (61.3%) HSV-2 negative (index value <0.9)
- 1684 Intervention, 1709 Controls
- HSV-2 acquisition defined as an index value >1.5 during follow up

HSV-2 Incidence by Study Group and Follow-up Interval

	Intervention group	Control group	Incidence rate ratio (95% CI)	p value
**0-6 months follow-up interval**				
Number of participants	1576	1608		
Incident events	26	39		
Person-years	781.5	794.5		
Incidence per 100 person-years	3.33	4.91	0.68 (0.40-1.14)	0.1242
**6-12 months follow-up interval**				
Number of participants	1550	1570		
Incident events	32	45		
Person-years	744.75	741.5		
Incidence per 100 person-years	4.3	6.07	0.71 (.44-1.14)	0.1355
**12-24 months follow-up interval**				
Number of participants	1314	1330		
Incident events	58	75		
Person-years	1279.25	1278.25		
Incidence per 100 person-years	4.53	5.87	0.77 (0.54-1.10)	0.1404
**Total 0-24 months follow-up**				
Number of participants	1576	1608		
Incident events	116	159		
Person-years	2805.5	2814.25		
Incidence per 100 person-years	4.13	5.65	0.73 (0.57-0.93)	0.0103

Kaplan-Meier Cumulative Probabilities of HSV-2 acquisition

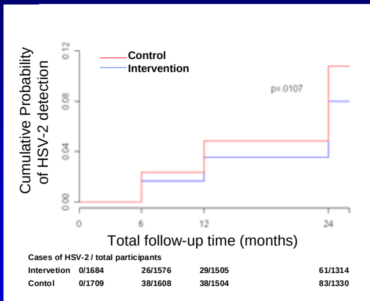

Cox HR estimate = .733 (.489, .977)

HIV acquisition by HSV-2 Infection Status and Study Arm

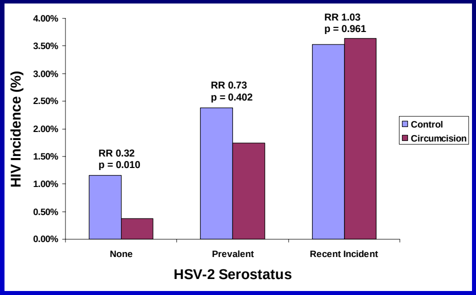

- **The indeterminant baseline samples (index values 0.9-1.5) were counted as initially negative.**

Structure of Two Rakai MC Trials

Screen male volunteers

HIV-neg men enrolled in NIH-funded RCT

- **Immediate MC**
- **MC delayed 24 mths (control)**

HIV+ men enrolled in Gates funded RCT

- **Immediate MC**
- **MC delayed 24 mths (control)**

Married men linked to consenting wives

M+/F- couples assessed for male-to-female HIV transmission

Surgical adverse events (AEs), wound healing and resumption of sex

- AEs in HIV+ and HIV-neg men
- Wound healing in HIV+ and HIV-neg
- Sex before healing and AEs

Surgery-related adverse events in circumcised HIV+ men compared to HIV-neg men

	HIV+ men % (N)	HIV-neg men % (N)
Mild	2.9 (12)	4.0 (94)
Moderate	3.1 (13)	3.1 (73)
Severe	0 (0)	0.2 (5)
Total	6.0 (25)	7.4 (172)
	Total # of surgeries = 420	Total # of surgeries = 2326

Completed wound healing by postoperative time

	HIV +		HIV-neg	
	Healed/ N	%	Healed/ N	%
30 days	287/ 393	73.0	1879/ 2258	83.2*
6 weeks	364/393	92.7	2163/ 2258	95.8**

*P < 0.001, **P = 0.006

Postoperative healing is slower in HIV+.
Most men healed by 6 weeks

AEs in couples initiating sex before and after healing certified

Resumed sex before healing certified		Resumed sex after healing certified	
AEs/N	%	AEs/N	%
35/316	11.1	162/2397	6.8

RR sex before/sex after healing 1.64 (1.16-2.32)

Genital Ulcer Disease (GUD) at FU, HIV+ intervention and control men

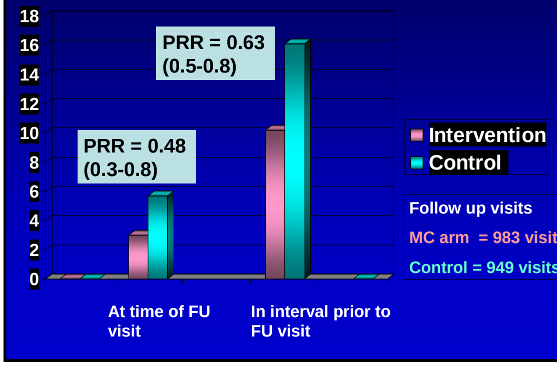

Follow up visits
MC arm = 983 visits
Control = 949 visits

MC and Female HIV/STI acquisition

Trial Profile

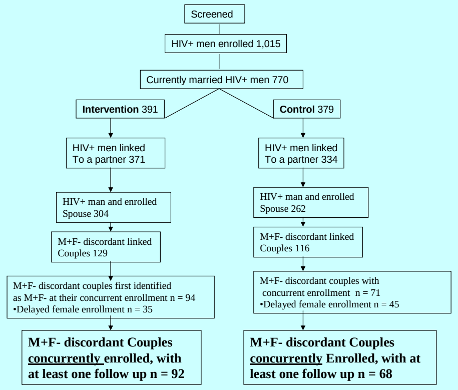

Male-to Female HIV transmission by study arm for concurrently enrolled couples

	Intervention		Control		IRR (95% CI)
	Transmis/ PY	Incidence /100 py	Transmis/ PY	Incidence /100 py	
0-24 mths	17 / 118	14.4	8 / 88	9.1	1.59 (0.7-4.3)

Includes 8 crossover men MC arm and 1 crossover in control arm.

Male-to-female transmission by timing of wound healing and resumption of sex, 0-6 month FU

Timing of resumption of sex	Intervention arm	
	Transmitted / N	%
Sex before wound healing was complete	5 / 18	27.8
Sex at or after wound healing	6 / 63	9.5
Early vs later resumption of sex	RR = 2.92 (1.01-8.46) . p = 0.06	
	Control arm	
	6 / 68	8.8%

Excludes couples who did not resume sex (3) and uncircumcised men (8)

BV, Trichomonas and GUD in HIV-neg female partners

- Identified HIV-negative women married to HIV-neg male trial participants with both partners enrolled at the same time

- Women followed at one year to detect:
- Self-reported vaginal symptoms
- Trichomonas by culture
- BV by Gram stain (Nugent's score)

Male Circumcision for Prevention of Vaginal Infections in Female HIV-negative Partners Trial Design

1608 HIV-negative women married to men in male circumcision trial were also enrolled

825 wives of HIV-negative men that were circumcised

783 wives of HIV-negative men in control arm

12 month follow up
>95% retention

GUD, vaginal discharge and dysuria symptoms
Bacterial vaginosis (Gram stain of vaginal swab)
Trichomonas vaginalis (InPouch TV™ Culture)

Wives of circumcised men in each study arm had similar sociodemographic and behavioral characteristics except for BV (control 38.3%, intervention 30.5%)

Vaginal symptoms at 12 months follow up by husband's study arm

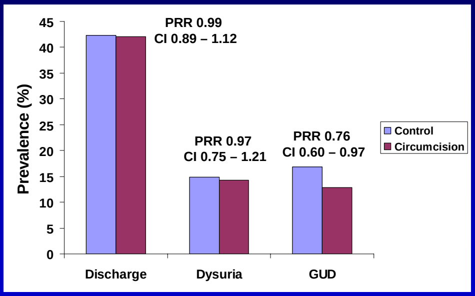

- **Significant reduction of GUD in wives of circumcised men (p = 0.03). No effect on discharge or dysuria.**

Vaginal infections at 12 month follow up by husband's study arm

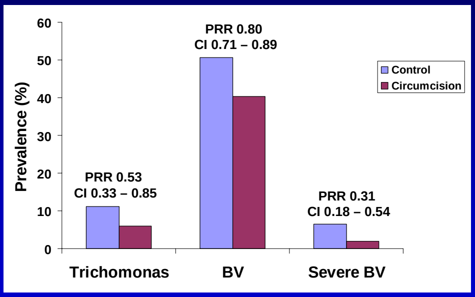

BV at Follow up by BV at Enrollment and Study Arm

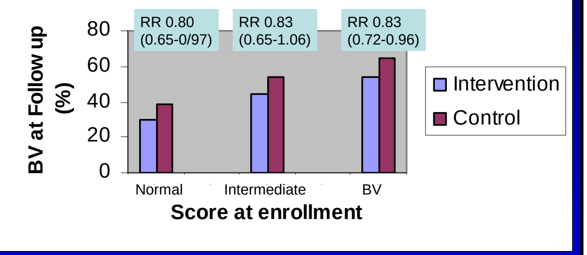

Reduced progression to BV and reduced persistence in women with initial BV

Summary

- HIV-neg men. Completed fu confirms long-term protection from HIV
- MC reduces HSV-2 acquisition in men
- Surgery as safe in HIV+ as in HIV-neg, but healing is slower in HIV+.
- AEs increased with early resumption of sex before wound healing. Recommend abstinence for 6 weeks postop?
- Reduced GUD in HIV+ men a direct benefit of MC
- No reduction of Male to female HIV transmission, possible increased risks with intercourse before completed wound healing
- MC reduces GUD, Tv and BV in female partners

CIRCUMCISION IN ZIMBABWE: THE INFLUENCE OF EPIDEMIOLOGICAL CONTEXT AND QUANTIFYING UNCERTAINTY IN PROJECTIONS

TB Hallett*, J Griffin, J Smith, KJ Singh, RG White, LJ Abu-Raddad, A Ghani, S Gregson, GP Garnett
Imperial College London
Bill & Melinda Gates Foundation
London School of Hygiene and Tropical Medicine
University of Washington
*timothy.hallett@imperial.ac.uk

Contents

Develop understanding of the problem

- **Qualitative insights:**
  - Relative benefit among men and women
  - Interactions with other interventions
  - Best age to circumcise
  - Impact of risk compensation and increased rates of t[illegible]

Provide reliable description of problem and make projections

- **Quantitative insights – for an intervention in Zimbabwe:**
  - Uncertainty in projections due to uncertain epidemiological context; conditions conducive to a successful intervention.
  - Grand uncertainty in key indicators due to context and nature of intervention
  - Key determinants of intervention impact.

Methods

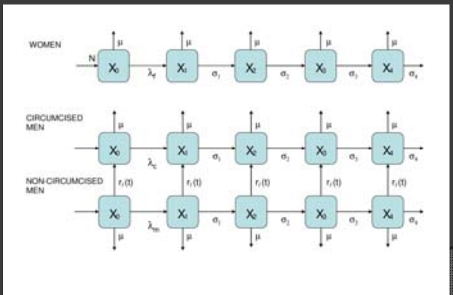

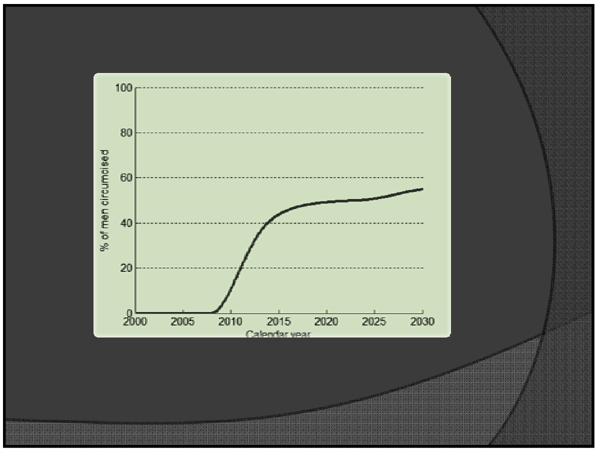

Qualitative insights:

- Relative benefit among men and women
- Interactions with other interventions
- Best age to circumcise
- Impact of risk compensation and increased rates of transmission

Benefits for men and women

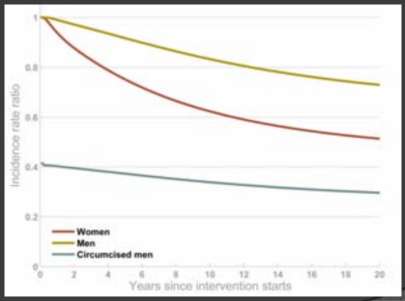

Time & Transmission

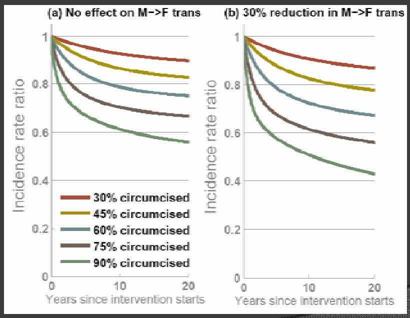

Qualitative insights:

- Relative benefit among men and women
- Interactions with other interventions
- Best age to circumcise
- Impact of risk compensation and increased rates of transmission

… with ART

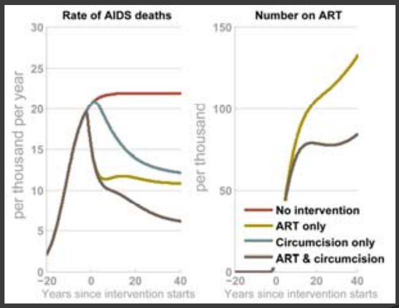

… with reductions in sexual risk behaviour

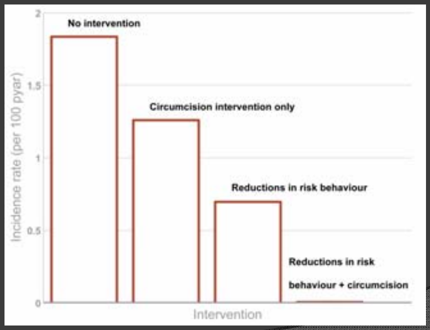

[Synergistic operation]

Qualitative insights:

- Relative benefit among men and women
- Interactions with other interventions
- Best age to circumcise
- Impact of risk compensation and increased rates of transmission

[From CDC report]

Qualitative insights:

- Relative benefit among men and women
- Interactions with other interventions
- Best age to circumcise
- Impact of risk compensation and increased rates of transmission

Risk compensation = less condom use with casual partners

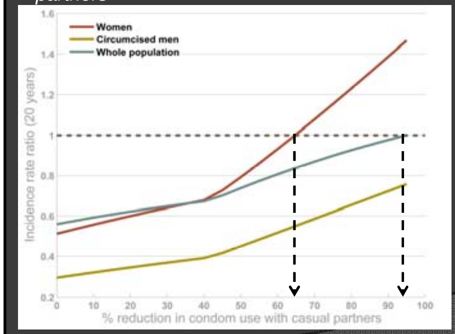

[parameter space plot]

Increased risk of transmission (x3) during wound healing.

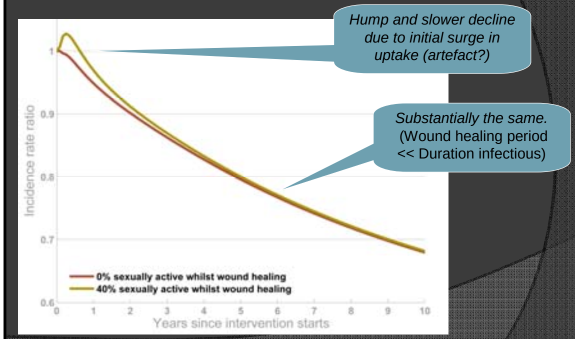

The Testing Issue:

HIV-testing before circumcision:

- Avoids circumcising HIV+ men;
- Avoids putting some individual women at increased risk
- But… may reduce uptake, particularly among high-risk men
- (And the impact of the intervention is greater if high-risk men circumcised).

Individual-level perspective:	Do not circumcised HIV+ men (test all);
Population-level perspective:	Circumcise all men, even if HIV+ (no testing);

Quantitative insights – for an intervention in Zimbabwe:

- Uncertainty in projections due to uncertain epidemiological context; conditions conducive to a successful intervention.
- Grand uncertainty due to context and nature of intervention
- Key determinants of intervention impact.

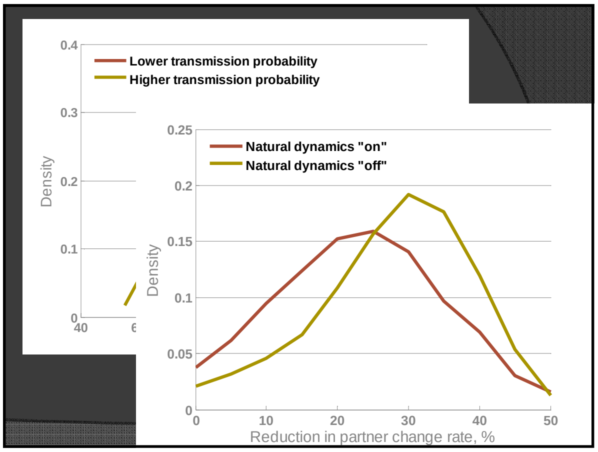

Apply <u>same</u> circumcision intervention to alternative 'epidemiological contexts'

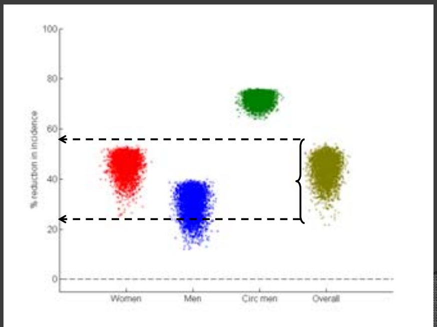

	Parameter	SRCC	95% Confidence Interval		% Variation explained
Urban	Relative change in condom use with non-regular partners	0.35	0.34	0.36	12%
	Risk ratio in medium-risk group (females)	-0.30	-0.32	-0.29	9%
	Risk ratio in medium-risk group (males)	-0.25	-0.26	-0.23	6%
	Relative change in rate of partner change rate	-0.22	-0.24	-0.21	5%
				R-squared (full model):	*84%*
Rural	Fraction of high-risk females in highest risk group	-0.34	-0.37	-0.31	11%
	Relative change in condom use with non-regular partners	0.33	0.31	0.35	11%
	Fraction of females in higher-risk groups	-0.31	-0.34	-0.27	9%
	Fraction of high-risk males in highest risk group	-0.26	-0.29	-0.24	7%
	Relative change in rate of partner change rate	-0.23	-0.25	-0.21	5%
	Fraction of non-regular partnerships with consistent condom use	0.23	0.21	0.24	5%
	Mean partner change rate (males)	0.22	0.19	0.24	5%
	Number of sex acts in a non-regular partnership per year	-0.21	-0.24	-0.19	5%
				R-squared (full model):	*62%*

What conditions are conducive to circumcision interventions?

- "Distributed risk"
  - High prevalence mediated by many people having moderate risk, *cf.* few people having very high risk. [*Effect of circumcision among highly-exposed men is nothing!*]
- High condom use in casual partnerships / casual partnerships short.
  - Circumcision works best when condom use is higher in the partnerships likely to lead to HIV-exposure.
- Recent reductions in risk behaviour
  - Independent behavioural changes have weakened chains of transmission generally.

Quantitative insights – for an intervention in Zimbabwe:

- Uncertainty in projections due to uncertain epidemiological context; conditions conducive to a successful intervention.
- Grand uncertainty due to context and nature of intervention
- Key determinants of intervention impact.

Please contact the author(s)
for more information

… apply range of interventions to the different epidemiological contexts

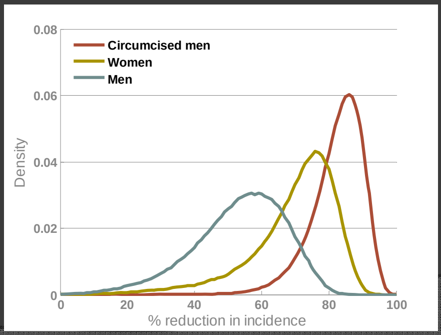

Same intervention:

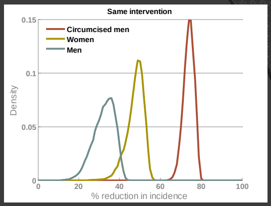

Different intervention:

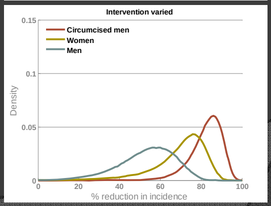

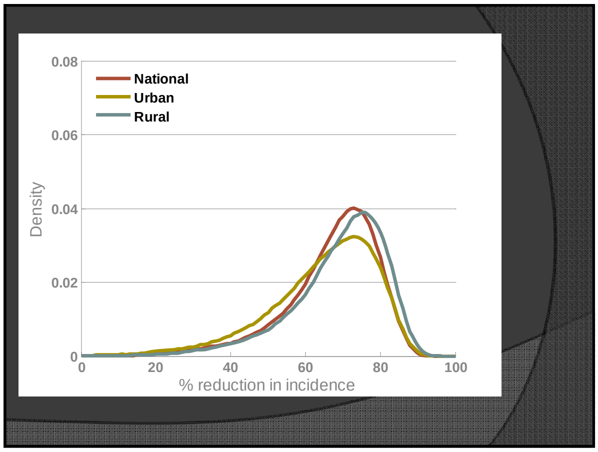

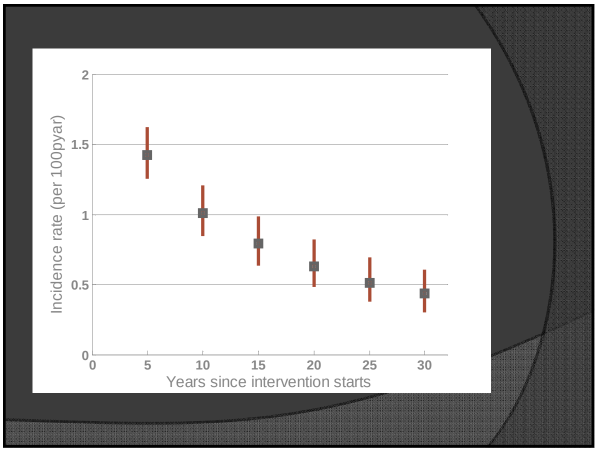

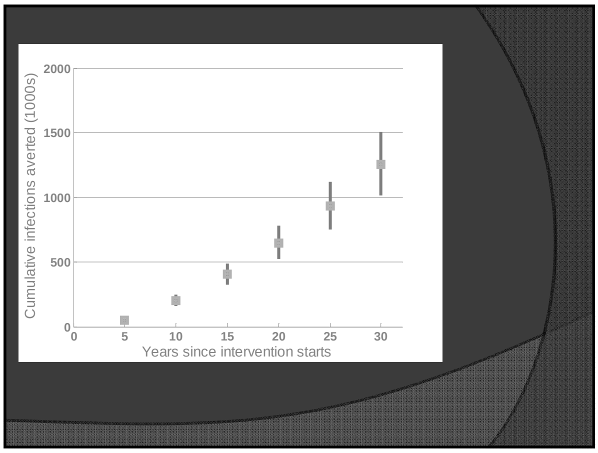

Operations per infected averted

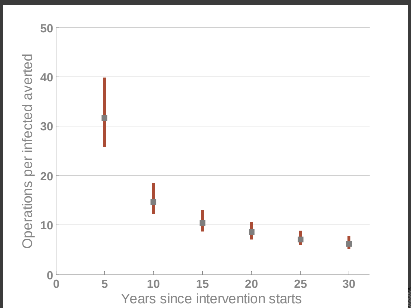

Quantitative insights – for an intervention in Zimbabwe:

Uncertainty in projections due to uncertain epidemiological context; conditions conducive to a successful intervention.

Grand uncertainty due to context and nature of intervention

Key determinants of intervention impact.

	Intervention parameter	Coefficient	% Variation explained	R-squared of full model
5 years:				
Overall	Relative risk of HIV transmission	-0.847	72%	
	HIV+ get circumcised	0.352	12%	86%
Circumcised men	Relative risk of HIV acquisition	-0.910	83%	
	Increase in partner change rate	-0.260	7%	
				96%
Women	Relative risk of HIV transmission	-0.871	76%	
	HIV+ get circumcised	0.376	14%	91%
Men	Relative risk of HIV transmission	-0.770	59%	
	HIV+ get circumcised	0.352	12%	
	Increase in partner change rate	0.343	12%	87%
30 Years:				
Overall	Relative risk of HIV transmission	-0.824	68%	75%
Circumcised men	Relative risk of HIV acquisition	-0.597	36%	
	Relative risk of HIV transmission	-0.564	32%	74%
Women	Relative risk of HIV transmission	-0.862	74%	81%
Men	Relative risk of HIV transmission	-0.747	56%	64%

Key determinants of intervention impact:

- Short-term
  - Circumcised men: Reduction in acquisition
  - Everyone else: Reduce in transmission
- Long-term
  - Circumcised men: Reduction in acquisition; Reduction in transmission
  - Everyone else: Reduction in transmission
- Association with other parameters exists but it swamped by association between these two key parameters:
  - (Even risk compensation/ transmission during would healing).
  - (Even parameters describing epidemiological context – *these add to scatter though*)

Overall conclusions

- Possible to draw broad conclusions on how interventions operate in wide-range if circumstances.
- Model make very useful insights – and the emerging consensus is especially important (more in a minute).
- Uncertainty in quantitative predictions is great and permanent:
  - No more RCTs on transmission?;
  - No vast increase in availability of sex-partner network data on national scale planned?
- Therefore, crucial to fairly represent this in any statement, model, overall finding, etc.

Structural Uncertainty & Model Comparisons

- *All conclusions based on accepting that model structure is true.*

  (Even the uncertainty analyses, which carefully accounts for errors in data, fully accepts model structure).

- **To what extent are the conclusions dependent on the structural assumptions?**

Example of issues where structure might interfere with conclusions:

Partnership Duration:

Some model do not explicitly track duration of partnerships -- all partnerships instantaneous but many partnerships are actually long-term.

Since protective benefit depends on exposure, (men in long-term partnerships get proportionately less benefit if exposed), do we over-estimate benefit of circumcision?

→ Depends on complex structure of network, because we also under-estimate benefit of circumcision for men in very short-term partnerships & how frequently are faithful men held in partnerships with infected women?

Best Age to Circumcise:

This will depend on pattern of mixing by age – older men disproportionate influence on HIV transmission to young women…. stronger indirect effect by circumcising men at older age?

Pattern of condom use varies by age… young men tend to use condoms more…. Stronger direct effect from circumcising men at younger age?

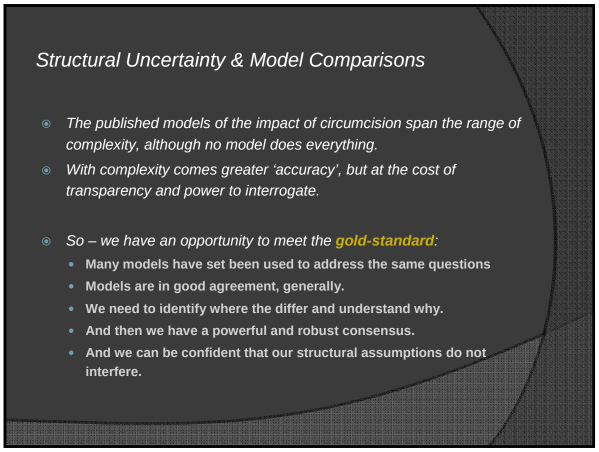

Structural Uncertainty & Model Comparisons
The published models of the impact of circumcision span the range of complexity, although no model does everything.
With complexity comes greater 'accuracy', but at the cost of transparency and power to interrogate.
So – we have an opportunity to meet the gold-standard:
Many models have set been used to address the same questions
Models are in good agreement, generally.
We need to identify where the differ and understand why.
And then we have a powerful and robust consensus.
And we can be confident that our structural assumptions do not interfere.

Male circumcision for HIV prevention: who, what, when?

UNAIDS/ WHO/ SACEMA Consultation

London, March, 2008

Richard White
and colleagues

Aim and objectives

- Explore the population level impact of various scenarios of the rollout of MC in an urban population in East Africa
  - Estimate the population level impact of a linear increase in the proportion of circumcised HIV-uninfected males in the targeted age groups
    - from 25% (pre-intervention prevalence) to 75% over 5 years,
    - with no risk compensation
  - Estimate magnitude of risk compensation required to negate impact of the plausible MC intervention, and the potential of increased condom use(due to counselling) to increase impact
  - Estimate population level impact of inadvertently circumcising HIV infected males
  - Estimate cost per HIV infection averted

Methods and assumptions (1)

- Individual-level stochastic model *STDSIM*
  - simulates the spread of HIV and STIs
  - dynamic sexual network of simulated individuals and their sexual contacts
  - Present average of 500 iterations per scenario
- Baseline scenario
  - Fitted to the demographic, behavioural and epidemiological characteristics of the urban population (Kisumu) in East Africa in 1997 and 2006, and the impact of MC measured in young men in the Kisumu RCT (59%, 95% CI=30-76)
  - 25% of males already circumcised

Circumcision assumptions

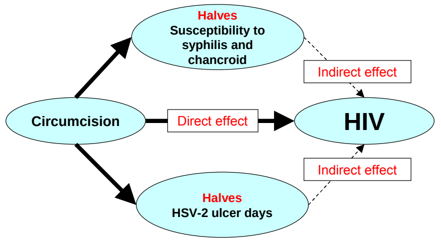

HIV- M: ► reduced susceptibility to HIV acquisition (fitted to RCT)
► double the risk of acquisition from HIV+ F over 6m in 15% M who resume sex before healing

HIV+ M: ► double risk of transmission to F over 6m, in 15% of men who resume sex before healing (only direct effect on M to F)

Methods and assumptions (2)

- Intervention scenarios
  - Intervention starts 1st January 2007
- Default scenario
  - Linear increase in proportion of HIV uninfected males in targeted age group from 25% to 75% over 5 years
  - no risk compensation
  - Target age groups: 15-24y, 25-39y 39-34y, 35-49y, 15-49y, neonates and 15 year olds
- Primary Outcomes
  - Impact on HIV incidence (%) :
    = (1 - mean IRR among MF 15-49 years old) * 100
  - Number of circumcisions per HIV-infection averted (MF 15-49y)
  - Over 2,5,10,20,30,40 and 50 years
- Plausible range
  - Based on 95% confidence estimates from the Kenyan circumcision trial

Methods and assumptions (3)

- Estimate cost per HIV infection averted
  - Assume cost of circumcision
    - Adults : $51 (33-69)
    - Neonates : $15 (7-25)
  - Future costs and effects were discounted at 3% per year, and cost-effectiveness is presented in present value
  - Compared to a recent estimate of the present value of lifetime treatment costs of an HIV infection in Africa ($3,469 in 2004US$), recalculated using a 3% discount rate and adjusted for inflation to 2007US$ ($4,043)

Methods and assumptions (3)

- Alternative scenarios
  - Behaviour change
    - In default scenario condoms are used in 40% of casual and sex-contacts after 2000, with 10% failure
    - Vary condom use between 0-30% (risk compensation) and 50-80% (effective safe-sex counselling) in
      - Recently circumcised men
      - All circumcised men (including 25% already circumcised)
  - Direct effect on M to F HIV transmission probability
    - Assume 50% reduction

Methods and assumptions (4)

- Alternative scenarios continued
  - Effect of circumcising HIV infected males
    - Circumcise 15-49 year old males regardless of HIV status
      - With and without assuming circumcising males halves the male-to-female transmission probability of HIV
    - Keep number of circumcisions over each time period equal to that modelled in default scenario

Results: Baseline scenario

Population structure by age and sex

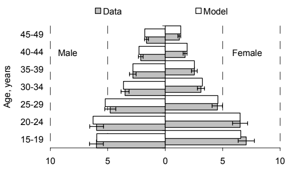

Prevalence of steady partnerships

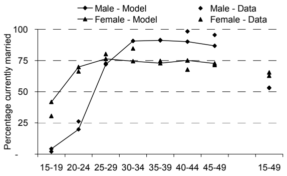

Results: Baseline scenario

STI prevalence

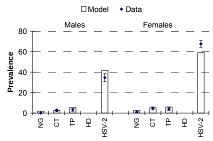

HIV prevalence trend

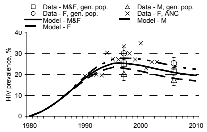

HIV prevalence by age - Males

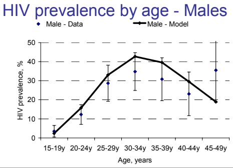

HIV prevalence by age - Females

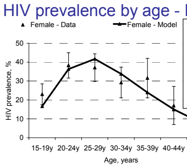

Age difference in casual and steady partnerships:
Data: 5 years
Model: 6.6 years

1) What is the overall impact on men?

Proportional reduction in incidence and number of circumcisions per HIV infection averted in men over 10 year or 20 years

Targeting 15-24 year old males

	Over 10 years	Over 20 years
HIV incidence reduction, % (15-49y)	14 (7-20)	25 (13-34)
Number of circ. per infection averted (15-49y)	13 (9-28)	6 (4-11)

2) What is the overall impact on women?

Proportional reduction in incidence women and number of circumcisions per HIV infection averted in women over 10 year or 20 years

Targeting 15-24 year old males

	Over 10 years	Over 20 years
HIV incidence reduction, % (15-49y)	5 (3-7)	17 (10-22)
Number of circ. per infection averted (15-49y)	30 (25-53)	8 (6-13)

3) How do the effects vary by the age group circumcised?

Ratio of total impact (Number of circ. per HIV inf. averted in MF over 5, 10, 20 & 30 years) for each target age group

relative to targeting 15-24 year olds

	5	10	20	30	5	10	20	30
15-24	1.0	1.0	1.0	1.0	24 (18-60)	9 (6-18)	3 (2-6)	2 (2-3)
25-29	1.8	1.3	0.9	0.7	13 (8-20)	7 (5-12)	4 (3-6)	3 (2-5)
30-34	1.8	1.2	0.7	0.6	13 (9-25)	8 (5-16)	4 (3-9)	4 (3-7)
35-49	1.0	0.6	0.4	0.3	24 (18-49)	15 (9-42)	9 (6-19)	7 (5-14)
15-49	1.3	1.1	1.0	1.0	19 (13-38)	8 (6-15)	3 (2-6)	2 (2-4)
Neonates				0.1				33 (23-75)
15y olds		0.2	0.6	0.7		47 (17-74)	6 (4-10)	3 (2-5)

3) How do the effects vary by the age group circumcised?

Ratio of total impact (Number of circ. per HIV inf. averted in MF over 5, 10, 20 & 30 years) for each target age group

relative to targeting 15-24 year olds

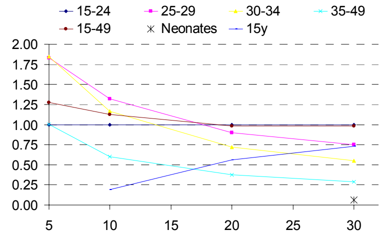

4) How do the effects vary with time scale?

Number of circumcisions per HIV-infection averted in adults aged 15-49 years olds over time

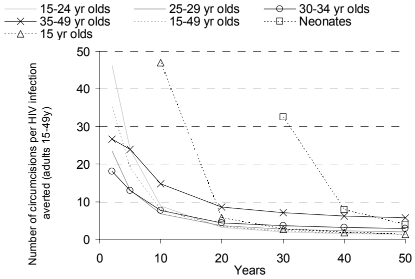

5) Do sexual mixing patterns affect the impact results?

Impact on HIV incidence in 15-24 year old females by circumcising males aged 25-29 years old

Targeted males:

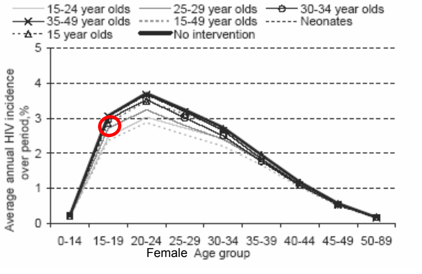

6) How are the impacts affected if other prevention interventions are scaled up at the same time?

Impact in scenario in which HIV incidence in 2020 is reduced from 2.6 to 1.1/100pyrs by increasing condom use in 2000

Targeting 15-49 year old males

		10 years	20 years
HIV incidence reduction, % (15-49y)	- Default	16	27
	- Lower incidence	15	23
Number of circ. per infection averted (15-49y)	- Default	8	3
	- Lower incidence	14	6

7) What is the effect of risk compensation?

Reduction in impact given full risk compensation?

Impact of varying proportion of sexual contacts in casual and sex-worker contacts that are protected by condoms (with 10% failure rate) between 0% and 80% compared to 40% in the default scenario.

Newly circumcised males

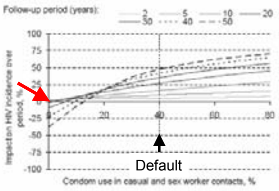

All circumcised males

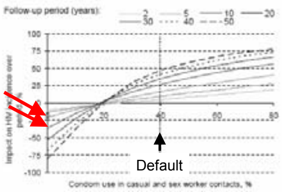

8) What are the discounted savings?

Cost required to avert one HIV infection in adults aged 15-49 years, over time compared with present value lifetime treatment costs (2007US$)

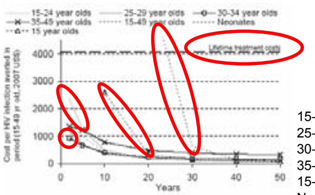

	10	20	30
15-24 yr olds	484	189	123
25-29 yr olds	356	197	148
30-34 yr olds	401	243	196
35-49 yr olds	777	475	392
15-49 yr olds	431	195	132
Neonates		6,546	624
15 yr olds	2,597	334	161

Present value lifetime treatment cost = $4,043 (2007US$)

Population level impact in males of circumcising HIV infected males (15-49y)

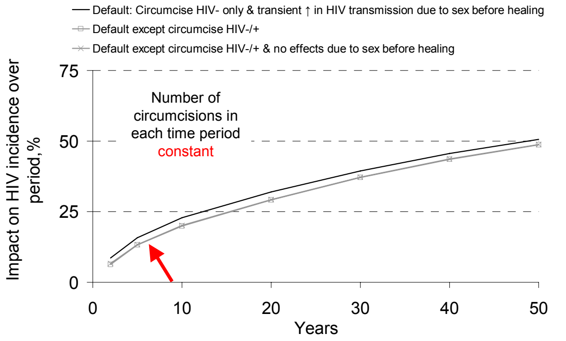

Population level impact in females of circumcising HIV infected males (15-49y)

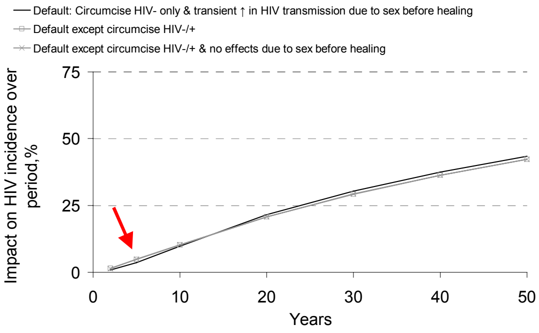

Population level impact in females of assuming circumcision halves the per-contact male-to-female HIV transmission probability

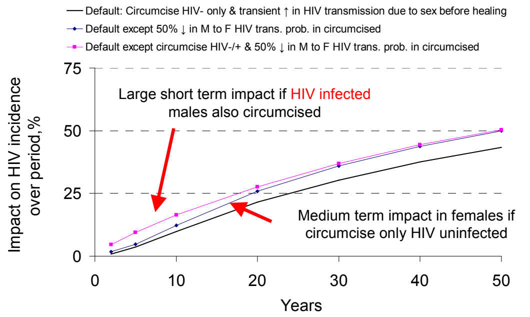

Limitations

- May have overestimated impact
  - Have assumed impact of MC on ulcers
  - Falls in HIV incidence due to other interventions
- May have underestimated impact
  - No direct effect on M>F transmission
  - Over longer term because of increasing risk in effective intervention scenarios

Summary and policy implications

- Short term impact in women will be small unless strong direct impact on M>F transmission *and* circumcision of HIV+s
- Difference M vs F will reduce over time
- Over first 10 years, targeting 25-34 year olds may be more effective per-circumcision than targeting younger males
  - => Change recommended priority age group to 15-34?
- Will wait 20-40 years to get population level impact via neonates

Summary and policy implications

- To maximise the number of infections averted, scale-up quickly with high coverage, while maintaining quality(!)
- Risk compensation
  - If limited to newly circumcised, it will reduce impact over medium term & potential to negate impact over long term
  - Effect is strongly dependent on whether already-circumcised change behaviour
    => target IEC at already-circumcised?

Summary and policy implications

- Circumcising HIV+ males
  - Will reduce population level impact in males in short term *if* coverage of HIV-uninfected males not maintained
  - Population level impact in females
    - may be small *if* only small proportion of men resume sex before wound healing and circumcision lowers STI rates
    - *or* may increase markedly *if* there is a direct impact on M>F HIV transmission

Acknowledgements

London School of Hygiene and Tropical Medicine
Judith Glynn, Helen Weiss, Kate Orroth, Esther Freeman, Richard Hayes
Institute of Tropical Medicine, Antwerp
Anne Buvé
Erasmus Medical Center, Rotterdam
Roel Bakker
UCSF, US
Craig Cohen
Johns Hopkins, US
Ron Gray
Members of the Study Group on Heterogeneity of HIV Epidemics in African Cities

Funding
Wellcome Trust / Medical Research Council
