# Supplementary material for: Male Circumcision for HIV Prevention in High HIV Prevalence Settings: What Can Mathematical Modelling Contribute to Informed Decision Making?
Source: PLoS Med. 2009 Sep 8;6(9):e1000109. doi: 10.1371/journal.pmed.1000109 (PMC2731851; doi:10.1371/journal.pmed.1000109)
Supplement: Text S1 — Details of mathematical models presented to the expert group. Full details and technical specifications of the models presented have been published in the peer-reviewed literature and the presentations made to the Expert Group are provided in Text S2. This document provides a brief unified summary of the setting and the model structure, including key assumptions, along with the analyses performed using the models. (0.63 MB PDF) [file pmed.1000109.s007.pdf]

**Informing Decision Making on Male Circumcision for HIV Prevention in High Prevalence Settings: What Mathematical Modelling Can Contribute**

## SUPPORTING INFORMATION

Text S1: Details of Mathematical Models Presented to the Expert Group

Full details and technical specification of the models presented have been published, and the presentations made to the Expert Group are provided in *Text S2*. This document provides a brief unified summary of the setting, model structure, including key assumptions, and the analyses performed using the models.

**<u>Hallett *et al.*</u>**

Hallett TB, Singh K, Smith JA, White RG, Abu-Raddad LJ, Garnett GP. *Understanding the impact of male circumcision interventions on the spread of HIV in southern Africa*. PLoS ONE 2008,3:e2212.

**Published Abstract:**

***Background:*** *Three randomised controlled trials have clearly shown that circumcision of adult men reduces the chance that they acquire HIV infection. However, the potential impact of circumcision programmes – either alone or in combination with other established approaches – is not known and no further field trials are planned. We have used a mathematical model, parameterised using existing trial findings, to understand and predict the impact of circumcision programmes at the population level.*

***Findings:*** *Our results indicate that circumcision will lead to reductions in incidence for women and uncircumcised men, as well as those circumcised, but that even the most effective intervention is unlikely to completely stem the spread of the virus. Without additional interventions, HIV incidence could eventually be reduced by 25-35%, depending on the level of coverage achieved and whether onward transmission from circumcised men is also reduced. However, circumcision interventions can act synergistically with other types of prevention programmes, and if efforts to change behaviour are increased in parallel with the scale-up of circumcision services, then dramatic reductions in HIV incidence could be achieved. In the long-term, this could lead to reduced AIDS deaths and less need for anti-retroviral therapy. Any increases in risk behaviours following circumcision , i.e. 'risk compensation', could offset some of the potential benefit of the intervention, especially for women, but only very large increases would lead to more infections overall.*

***Conclusions:*** *Circumcision will not be the silver bullet to prevent HIV transmission, but interventions could help to substantially protect men and women from infection, especially in combination with other approaches.*

This model was a simple deterministic compartmental model representing the heterosexual spread in Zimbabwe, or other similar settings in sub-Saharan Africa with HIV prevalence of approximately 20% and low rates of circumcision before the intervention starts.

To capture the heterogeneity in the number of sexual partners, men and women in the model were stratified into risk groups that form different numbers of sexual partnerships. Those in the higher risk groups tend to form more partnerships, but each of these partnerships comprises fewer sex acts and condom use is greater. Men and women form partnerships so that it is more likely that high risk individuals form partnerships with one another. Published data from eastern Zimbabwe were used to inform these parameters specifying sexual behaviour (although the broad behavioural patterns are similar to reports in other settings).

Based on observational data from several longitudinal studies, the course of infection is represented by individuals progressing through several stages: acute infection (short duration, high infectiousness), latent infection (long duration, low infectiousness) and pre-AIDS (short duration, high infectiousness). After pre-AIDS, a fraction of individuals develop full-blown AIDS and die, whist others start anti-retroviral therapy (ART) and survive for eight years with very low infectiousness. In this model, the fraction of individuals that can start treatment increases from 0% two years before the circumcision intervention starts (which we take to be approximately equal to calendar year 2005) to 28% within two years (i.e. 2007) – this is typical for sub-Saharan Africa. It was optimistically assumed that ART coverage will plateau at 90% by 2020. If some risk groups suffer greater AIDS-related mortality than others, the model

allows individuals to move between groups so that the overall mean and distribution of risk in the population is held constant.

Uncircumcised men can be circumcised in the intervention and it is assumed that circumcised men are 60% less likely to acquire infection in each sex act that they are exposed to HIV. The rate at which men are circumcised in the model is such that the eventual level of coverage of circumcision (fraction of men circumcised) is reached within 5 years of the intervention starting. A range of coverage levels were experimented with, ranging from 30 to 90%. In most simulations no effect on male-to-female transmission is assumed. In some simulations it is assumed that there is short period of wound healing (2 months) immediately following the operation, during which time most men are not sexually active but the chance of transmission to women per sex act may be elevated.

The main limitation of the model is the simplified way it represents the actual network of sexual partnership formation and the varied duration and type of sexual partnership. The wide range of possible sexual network structure are crudely captured with a small numbers of parameters determining the extent to which individuals at most risk of infection form partnerships with others at most risk, and the relative chance of transmission in casual and regular sexual partnerships. However, varying these parameter did not qualitatively affect the conclusions drawn.

This model was used to address the following issues:

1. What is the likely reduction in HIV incidence, given certain fractions of men (30-90%) being circumcised in the intervention? And, how does it change over time?
2. How is the impact of the circumcision intervention distributed between the men being circumcised, men not being circumcised and women?
    a. Does this change over time, or with the scale of the intervention?
    b. How is this affected if circumcision also reduces the chance of transmission from men to women?
3. How will circumcision interventions interact with existing interventions?
    a. Are there potential synergies with other interventions changing levels of sexual behaviour?
    b. Are there potential synergies with the scale-up of ART?
4. Is there the potential for perverse impacts on HIV spread from scale up of circumcision?
    a. How is the impact of circumcision interventions affected if the men that are circumcised reduce the frequency that they use condoms in their casual partnerships?
    b. How is the impact of circumcision interventions affected if there is a brief period of wound healing, during which some men are sexually active, and there is an increased chance of transmission to women at this time?

In addition, an age-structured version of the model has also been developed and published (Manicaland HIV/STD Prevention Project 2008, available from the authors). This was used to investigate how the impact of circumcision interventions is affected by alternative forms of targeting to different age-groups. This incorporated the typical pattern of sexual mixing whereby young women form most partnerships with men 5-10 years older. It was found that, over the first 30 years, an intervention targeted to men aged 20-29 years could lead to greatest reduction in incidence. In the longer term, circumcising infants or boys (younger than 19) could lead to a greater reduction in incidence; however, no impact would be detected for the first 20 years of the intervention.

**White *et al.***

White, R. G., J. R. Glynn, K. K. Orroth, E. E. Freeman, R. Bakker, H. A. Weiss, L. Kumaranayake, J. D. F. Habbema, A. Buve and R. J. Hayes (2008). "Male circumcision for HIV prevention in sub-Saharan Africa: who, what and when?" AIDS 22(14): 1841-1850.

**Published Abstract:**

***Background and objective:*** *Male circumcision (circumcision) reduces HIV incidence in men by 50–60%. The United Nations Joint Programme on HIV/AIDS (UNAIDS) recommends the provision of safe circumcision services in countries with high HIV and low circumcision prevalence, prioritizing 12–30 years old HIV-uninfected men. We explore how the population-level impact of circumcision varies by target age group, coverage, time-to-scale-up, level of risk compensation and circumcision of HIV-infected men.*

***Design and methods:*** *An individual-based model was fitted to the characteristics of a typical high-HIV-prevalence population in sub-Saharan Africa and three scenarios of individual-level impact corresponding to the central and the 95% confidence level estimates from the Kenyan circumcision trial. The simulated intervention increased the prevalence of circumcision from 25 to 75% over 5 years in targeted age groups. The impact and cost-effectiveness of the intervention were calculated over 2–50 years. Future costs and effects were discounted and compared with the present value of lifetime HIV treatment costs (US$ 4043).*

***Results:*** *Initially, targeting men older than the United Nations Joint Programme on HIV/ AIDS recommended age group may be the most cost-effective strategy, but targeting any adult age group will be cost-saving. Substantial risk compensation could negate impact, particularly if already circumcised men compensate. If circumcision prevalence in HIV-uninfected men increases less because HIV-infected men are also circumcised, this will reduce impact in men but would have little effect on population-level impact in women.*

***Conclusion:*** *Circumcision is a cost-saving intervention in a wide range of scenarios of HIV and initial circumcision prevalence but the United Nations Joint Programme on HIV/AIDS/WHO recommended target age group should be widened to include older HIV-uninfected men and counselling should be targeted at both newly and already circumcised men to minimize risk compensation. To maximize infections-averted, circumcision must be scaled up rapidly while maintaining quality.*

This study primarily addressed the following questions:

1. What is the likely reduction in HIV incidence (effectiveness) if the prevalence of male circumcision was increased from 25% to 75% over 5 years?
2. What is the likely cost-effectiveness of increasing the prevalence of male circumcision?
3. How does the effectiveness and cost-effectiveness change over time?
4. How does the effectiveness and cost-effectiveness vary by priority age-group?
5. How does the effectiveness and cost-effectiveness vary by coverage?
6. How does the effectiveness and cost-effectiveness vary by time to scale-up?
7. How does the effectiveness and cost-effectiveness vary by the amount of risk compensation in circumcised and uncircumcised males?
8. How does the effectiveness and cost-effectiveness vary by the proportion of circumcised males who are already HIV-infected?

9. How is the effectiveness and cost-effectiveness affected if circumcision also reduces the probability of transmission from men to women?

*METHODS*

*Model*

An individual-based stochastic model was used in this study. It simulates the natural history and heterosexual transmission of HIV and other STIs in a population consisting of individuals with characteristics that can change over time. The formation and dissolution of heterosexual relationships and transmission of STIs during contacts between sexual partners are modelled as stochastic events [1, 2]. The model allows for the simultaneous and interactive simulation of up to 16 different STIs (see supporting-material: section-S1 in [3]). The model has been used to explore the impact of STI treatment, vaccination and male circumcision for HIV-1 prevention in Africa, the heterogeneous spread of HIV-1 in Africa, and the diverging HIV-1 and HIV-2 prevalence trends in West Africa [3-12].

*Baseline scenario*

The STI natural history and the characteristics of the simulated population used in this study were based on extensive literature reviews and previously published work [6, 7, 10]. The model was fitted to the demographic, behavioural and epidemiological characteristics of a typical high-HIV, low-circumcision prevalence population (Kisumu, Kenya) for the years 1997 and 2006 and to available data on trends over time as detailed in earlier publications [6, 10], except that the mean survival time from HIV infection to death was increased from 10 to 11 years in line with the findings of a recent meta-analysis [13], and an additional increase in condom use rates was simulated in 2000 (to 40% of casual and sex worker contacts) in Kisumu to fit recent falls in HIV prevalence [3]. HIV, herpes simplex virus type 2 (HSV-2), chancroid, syphilis, gonorrhoea and chlamydia were simulated. STIs were assumed to enhance HIV susceptibility and infectivity. The assumed per-contact cofactor effects [6] reflected their relative clinical severity [14]. Multiple cofactor effects in the same individual were assumed to sum and overall cofactor effect for each partner in HIV-discordant partnerships to multiply (see supporting-material: section-S1 in [3]). In line with data, we assumed that 25% of men were already circumcised before the start of the intervention [6].

In the model, HIV was represented by four stages: primary, asymptomatic, symptomatic and AIDS [6]. The use of four stages for HIV infection allowed us to simulate changes in infectivity over the course of the infection [15]. Infectivity of HIV was simulated as high in the primary stage, lower during the asymptomatic stage and then increasing again during the symptomatic and AIDS stages [15]. The a-priori ranges for HIV transmission probabilities in each stage were based on a recent review of per contact transmission probabilities [16].

*Simulated effects of male circumcision*

Three direct and three indirect effects of male circumcision on HIV transmission we assumed in this study. Lack-of-circumcision was assumed to directly increase the risk of HIV acquisition in males. The magnitude of the per-contact cofactor for HIV acquisition in males was fitted to empirical individual-level data from the Kenyan trial[17](see below). In this study we also assumed two direct effects due to sexual intercourse before circumcision wound-healing. Among HIV-infected men in the Ugandan trial, there was a non-significant increase in the proportion of wives infected within six months of circumcision in men who resumed sex before wound-healing(25%) compared to those who did not(11%)[18]. Using these data and assuming that the median time to wound-healing was four-weeks in HIV-uninfected men[19], we used a Bernoulli model to calculate that the per-contact cofactor for HIV-infectiousness in contacts between HIV-infected males who resume sex before wound-healing, and their HIV-uninfected partners, was 9.8(supporting-material:section-S2 in [3]). We assumed 15% of HIV-infected males resumed sex before wound-healing[20]. When fitting the model to the trial data in this study we also assumed that 15% of HIV-uninfected males were subject to the same increased risk of HIV acquisition in the month following their circumcision in contacts with HIV-infected females, as postulated[21].

Much uncertainty remains in the effect of circumcision on the acquisition and transmission of cofactor STIs[17, 22-25]. In this study we assumed three indirect effects. We assumed that lack-of-circumcision doubled the per-contact risk of syphilis and chancroid acquisition(but had no effect on HSV-2 acquisition)[24] and HSV-2 ulcer point-prevalence was reduced by 50% in circumcised/HSV-2-infected males, in line with data suggesting that circumcision halves genital ulcer rates[17, 23].

*Fitting to individual-level impact data*

In this study we simulated three scenarios of the individual-level impact of male circumcision in previously uninfected males on HIV-incidence corresponding to the central estimate(59%) and the upper(76%) and lower(30%) 95%-confidence limits from the intervention trial in Kenya[17]. In each scenario the projected impact of the simulated intervention was fitted to the central, upper or lower estimate of impact from the trial by varying the magnitude of the lack-of-circumcision cofactor for HIV acquisition in males and refitting the observed HIV prevalence in 1997 and 2006. The simulated trial intervention circumcised 100% of 15-24 year old HIV-uninfected males on 1/1/2004. Incidence rate-ratios in this age-group were calculated over two-years, corresponding to the median follow-up period[17].

In this study we used the fitted model to estimate the proportion of the observed trial impact that was due to direct and indirect effects of circumcision, by simulating scenarios in which the direct and indirect effects on HIV were removed in turn.

*Simulated interventions*

The three baseline scenarios, fitted to the central, upper and lower bounds of individual-level impact, were then used to estimate the population-level impact of various scenarios of the roll-out of circumcision. All simulated interventions were simulated to start on 1/1/2007. Our ‘Default’ intervention resulted in a linear increase in the proportion of circumcised HIV-uninfected males in the targeted age-group, from 25%(pre-intervention prevalence), to 75% 5 years later with no risk-compensation. In separate simulations the following age-groups were targeted: 15-24,25-29,30-34,35-49,15-49, neonates and 15 year olds. HIV-uninfected males aging into the targeted age-group were also circumcised at the appropriate prevalence for that year of the intervention. This maintained circumcision prevalence in the targeted age-group over time and led to increases in circumcision prevalence in older age-groups as circumcised males aged. Results were based on means over 500 simulation runs.

The (cost-)effectiveness of the intervention was calculated over 2,5,10,20,30,40 and 50 years. In this study we allowed for varying time-horizons to reflect different time-preferences by society.

Impact on HIV-incidence was calculated as one minus the mean annual incidence rate-ratio in 15-49 year olds over the period. Over the same periods we also calculated the number of HIV infections averted in adults 15-49 years old per 1000 circumcisions in men of all ages, and the cost per-HIV-infection-averted. Adult circumcision costs were based on the published data from the trials, and adjusted for inflation to constant 2007US$, using the US consumer price index. Our central, lower and upper estimates of the cost of an adult circumcision were $51, $33 and $69, based on 2007US$ and data from the South Africa intervention excluding publicity[26], the Kenyan intervention[27] and the Ugandan intervention[28], respectively. Costs were incremental to clinic facilities, and include the direct costs of a circumcision procedure including supplies and personnel. Costs also included capital equipment specific to circumcisions but not training. Neonatal circumcision costs in sub-Saharan Africa are poorly known but are likely to be lower than for adults. American studies suggest that neonatal circumcision costs could be as low as 10% of adult costs[29, 30]. Conservatively, we adjusted the adult costs for likely lower supply costs and shorter procedure time. We assumed that the central estimate was 30% of the adult cost ($15), with an upper and lower

estimate of $7 and $25. Future costs and effects were discounted at 3% per year, and cost-effectiveness was shown in present value terms[26]. Our cost per-HIV-infection-averted estimates were compared to a recent estimate of the present value of lifetime treatment costs of an HIV infection in Africa($3,469 in 2004US$)[31], recalculated using a 3% discount rate and adjusted for inflation to 2007US$($4,043).

*Scenario and sensitivity analysis*

In this study we explored various alternative scenarios of coverage, scale-up period, risk-compensation, circumcision of HIV infected males and other uncertain parameter values. We varied the coverage of HIV negatives between 25-100% in line with acceptability-data[32] and the scale-up period between 0-20 years. In this study we modelled two scenarios in which newly-circumcised men or all-circumcised men, changed their condom use rate from 40% to 0-30%(risk-compensation) and 50-80%(effective counselling). We simulated a scenario in which the same proportions of HIV-infected and HIV-uninfected males were circumcised, while ensuring that the number of circumcisions over each period was equal to that in the default scenario in which only HIV-uninfected males were circumcised. In this study we combined this with a scenario in which we assumed a strong direct effect of male circumcision on male-to-female HIV transmission. In this scenario, we assumed that the per-contact cofactor for HIV infectiousness for HIV-infected and circumcised males was 50% of that for HIV-infected uncircumcised males.

To assess the robustness of our results to different epidemiological scenarios and uncertainties in parameters known to affect (cost-)effectiveness, alternative scenarios were simulated and key parameter values were varied while refitting HIV-prevalence where appropriate, and the (cost-)effectiveness of the default intervention targeted at 15-49 year olds was recalculated. First, we removed the effects of circumcision on chancroid, syphilis and HSV-2. Second, we increased the proportion of males who resumed sex before wound-healing. Third, we explored the impact of the intervention in populations in which HIV-prevalence(a) remained around 25% after 1997, (b)fell more steeply after 1997 to 10% in 2020, and (c)was lower overall, peaking at 10% in 1997. These fits were obtained by varying condom use and risk behaviour rates(see supporting-material, section-S3 in [3], for full details). Fourth, we varied the baseline circumcision prevalence from 25% to 0% and 50%. Finally, we varied the unit circumcision-cost for adults/neonates between US$33/7 and US$69/25, respectively, and the discount-rate between 0% and 6%.

*DEFAULT SCENARIO RESUTS ARE SHOWN IN* [3]

*SCENARIO AND SENSITIVITY ANALYSIS RESULTS:*

*Coverage and rollout timing, and effect of behaviour change in circumcised males*

Both coverage and scale-up duration were approximately linearly related to intervention impact (Figure 3a/b in [3]). Increased risk behaviour in circumcised males reduced intervention impact, such that impact was negated if condom use reduced from 40% in casual and sex-worker contacts to 15% if changes were restricted to newly-circumcised males (Figure 3c in [3]), or 20% if changes occurred in all circumcised males(Figure 3d in [3]). More optimistically, a larger number of HIV-infections would be averted if counselling increased condom use.

*Effect of circumcising HIV infected males*

Circumcising the same proportion of HIV-infected as HIV-uninfected males had little effect on the population-level impact(Figure 4 in [3]). The projected impact on HIV-incidence in males

was slightly lower than when circumcision was restricted to HIV-uninfected males, primarily because fewer HIV-uninfected males were circumcised, rather than because recently circumcised males were assumed briefly more infectious and susceptible to HIV. The lower projected impact among males did not translate into lower impact among women, because it was offset by lower cofactor-STI rates in STI/HIV coinfected males.

Figure 4 in [3] also shows the impact of assuming that circumcision halves the male-to-female HIV transmission probability. If only HIV-uninfected males were circumcised, the short-term impact on HIV-incidence was predicted to be small because the reduced transmission probability only applied to HIV-infected males who had become HIV-infected after circumcision. Over the longer-term, the intervention impact was predicted to be larger than in the default scenario. If HIV-infected males were also circumcised, then the short-term impact on HIV-incidence in females was markedly increased(Figure 4 in [3]).

*Further sensitivity analysis*

The impact of the circumcision intervention was robust to the assumed effects of circumcision on chancroid, syphilis and HSV-2(supporting-material:section-S3 in [3]). Removing all indirect effects reduced impact on HIV-incidence over 50 years from 51% to 45% in males and 44% to 36% in females. The population-level impact of circumcision on HIV incidence was robust to increasing the assumed proportion of males who resumed sex before wound-healing to at least 60%. Impact was largely unaffected when HIV prevalence was stable or was lower overall, but the projected longer-term impact was slightly lower when HIV prevalence declined more steeply, because of falls in STI rates due to the higher simulated condom use rates. The intervention was less cost-effective when the HIV prevalence declined more steeply or was lower overall, and more cost-effective when HIV prevalence was stable. The impact of the circumcision intervention was also robust to the assumed baseline prevalence of circumcision, but the intervention became slightly more cost-effective at higher circumcision prevalences, and vice versa. The intervention was more cost-effective when the cost per-procedure was lower, and vice versa.

*References*


1. Korenromp, E.L., et al., *HIV spread and partnership reduction for different patterns of sexual behaviour - a study with the microsimulation model STDSIM.* Mathematical Population Studies, 2000. **8(2)**: p. 135-173.
2. Korenromp, E.L., et al., *Model-based evaluation of single-round mass STD treatment for HIV control in a rural African population.* AIDS, 2000. **14**: p. 573-593.
3. White, R.G., et al., *Male circumcision for HIV prevention in sub-Saharan Africa: who, what and when?* AIDS, 2008. **22**(14): p. 1841-1850.
4. White, R.G., et al., *Treating curable Sexually Transmitted Infections to Prevent HIV in Africa: Still an Effective Control Strategy?* J Acquir Immune Defic Syndr, 2008. **47**: p. 346-353.
5. Schmidt, W.P., et al., *Behaviour change and competitive exclusion can explain the diverging HIV-1 and HIV-2 prevalence trends in Guinea-Bissau.* Epidemiol Infect, 2008. **136**(4): p. 551-561.
6. Orroth, K.K., et al., *Understanding differences across the contrasting epidemics in East and West Africa: results from a simulation model of the Four Cities Study.* STI, 2007. **83**: p. i5-i16.
7. White, R.G., et al., *Can Population Differences Explain the Contrasting Results of the Mwanza, Rakai, and Masaka HIV/Sexually Transmitted Disease Intervention Trials?: A Modeling Study.* J Acquir Immune Defic Syndr, 2004. **37**(4): p. 1500-1513.

8. Korenromp, E.L., et al., *Determinants of the Impact of Sexually Transmitted Infection Treatment on Prevention of HIV Infection: A Synthesis of Evidence from the Mwanza, Rakai, and Masaka Intervention Trials.* J Infect Dis, 2005. **191**(Suppl 1): p. S168-78.

9. Orroth, K.K., et al., *Empirical observations underestimate the proportion of human immunodeficiency virus infections attributable to sexually transmitted diseases in the Mwanza and Rakai sexually transmitted disease treatment trials: Simulation results.* Sex Transm Dis, 2006. **33**(9): p. 536-44.

10. Freeman, E., et al., *The proportion of new HIV infections attributable to HSV-2 increases over time: simulations of the changing role of sexually transmitted infections in sub-Saharan African HIV epidemics.* STI, 2007. **83**: p. i17-i24.

11. White, R.G., et al., *Population-level effect of HSV-2 therapy on the incidence of HIV in sub-Saharan Africa.* Sex Trans Inf, 2008. **84**(S2): p. 12-18.

12. Freeman, E.E., et al., *Population-level effect of potential HSV2 prophylactic vaccines on HIV incidence in sub-Saharan Africa. http://dx.doi.org/10.1016/j.vaccine.2008.11.074* Vaccine, 2009. **27**: p. 940–946.

13. Todd, J., et al., *Time from HIV seroconversion to death: a collaborative analysis of eight studies in six low and middle-income countries before highly active antiretroviral therapy.* Aids, 2007. **21 Suppl 6**: p. S55-63.

14. Rottingen, J.A., D.W. Cameron, and G.P. Garnett, *A systematic review of the epidemiologic interactions between classic sexually transmitted diseases and HIV: how much really is known?* Sex Transm Dis, 2001. **28**(10): p. 579-97.

15. Wawer, M.J., et al., *Rates of HIV-1 Transmission per Coital Act, by Stage of HIV-1 Infection, in Rakai, Uganda.* J Infect Dis, 2005. **191**(9): p. 1403-9.

16. Boily, M.C., et al., *Heterosexual risk of HIV-1 infection per sexual act: a systematic review and meta-analysis of observational studies.* Lancet Infectious Diseases, In press.

17. Bailey, R.C., et al., *Male circumcision for HIV prevention in young men in Kisumu, Kenya: a randomised controlled trial.* Lancet, 2007. **369**(9562): p. 643-56.

18. WHO, *Study Presents New Information on Male Circumcision to Prevent Spread of HIV in Africa*. 2007, WHO/Rakai Health Sciences Program: Montreux.

19. Gray, R., *Personal communication: Email dated 25/1/2008*. 2008: Johns Hopkins.

20. Matovu, J.K., et al., *Roundtable: Sexually Transmitted Infection Management, Safer Sex Promotion and Voluntary HIV Counselling and Testing in the Male Circumcision Trial, Rakai, Uganda.* Reprod Health Matters, 2007. **15**(29): p. 68-74.

21. Hankins, C., *Roundtable: male circumcision: implications for women as sexual partners and parents.* Reprod Health Matters, 2007. **15**(29): p. 62-7.

22. Auvert, B., et al., *Randomized, controlled intervention trial of male circumcision for reduction of HIV infection risk: the ANRS 1265 Trial.* PLoS Med, 2005. **2**(11): p. e298.

23. Gray, R.H., et al., *Male circumcision for HIV prevention in men in Rakai, Uganda: a randomised trial.* Lancet, 2007. **369**(9562): p. 657-66.

24. Weiss, H.A., et al., *Male circumcision and risk of syphilis, chancroid, and genital herpes: a systematic review and meta-analysis.* Sex Transm Infect, 2006. **82**(2): p. 101-9; discussion 110.

25. Tobian, A., et al. *Trial of Male Circumcision: Prevention of HSV-2 in Men and Vaginal Infections in Female Partners, Rakai, Uganda*. in *14th Conference on Retroviruses and Opportunistic Infections*. 2008. Boston.

26. Kahn, J.G., E. Marseille, and B. Auvert, *Cost-effectiveness of male circumcision for HIV prevention in a South African setting.* PLoS Med, 2006. **3**(12): p. e517.

27. Krieger, J.N., et al., *Adult male circumcision: results of a standardized procedure in Kisumu District, Kenya.* BJU Int, 2005. **96**(7): p. 1109-13.

28. Gray, R.H., et al., *The impact of male circumcision on HIV incidence and cost per infection prevented: a stochastic simulation model from Rakai, Uganda.* Aids, 2007. **21**(7): p. 845-850.

29. Jayanthi, V.R., J.E. Burns, and S.A. Koff, *Postneonatal circumcision with local anesthesia: a cost-effective alternative.* J Urol, 1999. **161**(4): p. 1301-3.

30. Schoen, E.J., C.J. Colby, and T.T. To, *Cost analysis of neonatal circumcision in a large health maintenance organization.* J Urol, 2006. **175**(3 Pt 1): p. 1111-5.

31. Stover, J., et al., *The global impact of scaling up HIV/AIDS prevention programs in low- and middle-income countries.* Science, 2006. **311**(5766): p. 1474-6.

32. Westercamp, N. and R.C. Bailey, *Acceptability of male circumcision for prevention of HIV/AIDS in sub-Saharan Africa: a review.* AIDS Behav, 2007. **11**(3): p. 341-55.

**Nagelkerke et al**

Nico JD Nagelkerke, Stephen Moses, Sake J de Vlas, and Robert C Bailey. *Modelling the public health impact of male circumcision for HIV prevention in high prevalence areas in Africa.* BMC Infectious Diseases 2007, 7:16

**Published Abstract:**

***Background:*** *Recent clinical trials in Africa, in combination with several observational epidemiological studies, have provided evidence that male circumcision can reduce HIV female-to male transmission risk by 60% or more. However, the public health impact of large-scale male circumcision programs for HIV prevention is unclear.*

***Methods****: Two mathematical models were examined to explore this issue: a random mixing model and a compartmental model that distinguishes risk groups associated with sex work. In the compartmental model, two scenarios were developed, one calculating HIV transmission and prevalence in a context similar to the country of Botswana, and one similar to Nyanza Province, in western Kenya.*

***Results****: In both models, male circumcision programs resulted in large and sustained declines in HIV prevalence over time among both men and women. Men benefited somewhat more than women, but prevalence among women was also reduced substantially. With 80% male circumcision uptake, the reductions in prevalence ranged from 45% to 67% in the two "countries", and with 50% uptake, from 25% to 41%. It would take over a decade for the intervention to reach its full effect.*

***Conclusion****: Large-scale uptake of male circumcision services in African countries with high HIV prevalence, and where male circumcision is not now routinely practised, could lead to substantial reductions in HIV transmission and prevalence over time among both men and women.*

In this paper two different models were used. One a proportionate (random) “Anderson-May type” mixing model. This model is mathematically tractable, and equilibrium values for the basic reproduction number $R_0$, and HIV prevalence in equilibrium, both with 0% and 100% male circumcision, were calculated. However, this model was less appropriate for studying the dynamic behaviour of the HIV epidemic before and after the introduction of an MC programme. Also, we felt that in all likelihood high risk (specifically sex workers and their clients) and low risk individuals do not mix randomly. We therefore also developed a deterministic compartmental model that did have these features. Our projections of the impact of MC were mostly based on the latter model.

*Model structure*

The model structure of the compartmental model is shown in Fig. 1. The only mode of HIV transmission in the model is heterosexual transmission. The model subdivides the population according to different dimensions (gender, involvement in commercial sex, circumcision status, HIV infection status and stage of infection), into discrete high-risk (male clients and female sex workers for the high-risk groups) and low-risk (general population, not involved in paid sex) groups. Each of these groups is again subdivided into compartments of individuals who are either HIV infected or not, and, for males, whether they are circumcised or not. In addition, HIV infection is split into two compartments, early and late, to incorporate non-exponential progression to AIDS and mortality. Altogether 6 compartments for (living) women and 8 for (living) men are distinguished. For women these 6 compartments are:

1. F11. HIV negative low risk women, not involved in sex work.
2. F12. Women not involved in sex work, during the early stages of HIV infection.
3. F13. Women not involved in sex work, during late(r) stage HIV infection.
4. F21. HIV negative female sex workers.
5. F22. Female sex workers during the early stages of HIV infection.
6. F23. Female sex workers during late(r) stage HIV infection.

For men these 8 compartments are:

1. M11. Men who are not sex worker clients, HIV uninfected, uncircumcised.
2. M14. Men who are not sex worker clients, HIV uninfected, circumcised.
3. M12. Men who are not sex worker clients, during the early stages of HIV infection, either circumcised or not.
4. M13. Men who are not sex worker clients, during late(r) stage HIV infection, either circumcised or not.
5. M21. Male sex worker clients, HIV uninfected, uncircumcised. 6. M24. Male sex worker clients, HIV uninfected, circumcised.
7. M22. Male sex worker clients, during the early stages of HIV infection, either circumcised or not.
8. M23. Male sex worker clients, during late(r) stage HIV infection, either circumcised or not.

We did not incorporate an age-structure in the model in view of evidence that age-mixing in Sub-Saharan Africa is probably so large that ignoring age would not distort reality too much. It did however limit our choice of MC strategies to those that did not involve age-targeting. Interactions, i.e. sexual relationships with the opposite sex, in the model depend on the compartment (high-risk or low-risk) that individuals are in. For example, "non-client" men do not visit sex workers at all. HIV transmission between low risk population groups consists of

two types, *viz.* entering into a new marriage-like or other stable relationship, or through "leakage" from infected individuals, such as non-paid casual sex and all existing sexual relationships at the time an individual becomes HIV infected. The rate of becoming a sex-worker client, and the frequency of intercourse with sex-workers is controlled by independent mechanism, but the rate of becoming a sex worker and also the average number of client contacts per sex worker modeled as demand driven. Group membership (e.g. being a sex worker) of individuals changes over time (e.g. women may practice sex work for a few years only).

We set the parameters of the model to values obtained from epidemiological literature and by fitting the model to reported prevalence data from two regions in Africa where MC is uncommon (approximately 10% of all adult males) and HIV prevalence is high, *viz*. Nyanza Province in Kenya, and Botswana in southern Africa

On the basis of the Orange farm trial, the only one published at the time of developing the model, a 60% reduction in female-to-male transmission risk following MC was assumed. The risk of early resumption of intercourse, before wound healing, was not incorporated in the model.  For our main analyses we assumed absence of behavioral disinhibition (risk compensation, i.e. increased risk taking) after circumcision, and no effect of male circumcision on male-to-female transmission In our model specification, men were not circumcised  instantaneously, but uncircumcised men were recruited as a constant rate leading to a specified (50% or 80%) equilibrium prevalence of circumcision among HIV uninfected men. As initially most men are uncircumcised, the total number of men getting circumcised is higher initially than in equilibrium when circumcision numbers are just high enough to maintain equilibrium circumcision levels.

*Sensitivity analyses* For our main (baseline) model specifications, we assumed a relative risk of MC for female- to-male transmission of 0.4, no risk compensation (behavioral disinhibition), and no effect on male-to-female transmission. These three assumptions were subjected to sensitivity analyses as follows:

1) As a first sensitivity analysis we considered two "extreme" relative risks of the effect of circumcision on female-to-male transmission, *viz*. a RR of 0.25, and a RR of 0.6 for female-to-male transmission. These values correspond roughly to the per-protocol estimate of the RR from the Orange Farm trial, and the upper limit of the 90% CI of the RR, respectively, and thereby constitute moderately optimistic and pessimistic estimates. The high RR value of 0.6 also somewhat exceeds the estimated effects in Uganda and Kenya and thus seems to be a "safe" upper limit of the RR.

2) As a second sensitivity analysis we explored the effect of a (moderate) protective effect of MC on male-to-female transmission, which we had assumed absent in our main analysis. Instead of a relative risk of 1 for MC (i.e. no effect on male-to-female transmission), we assumed a relative risk of 0.75.

3) As a third sensitivity analysis, we considered a specific form of risk compensation, viz. that circumcised men would abandon condom use in high-risk sexual contacts (assumed to be 20% effectively initially).

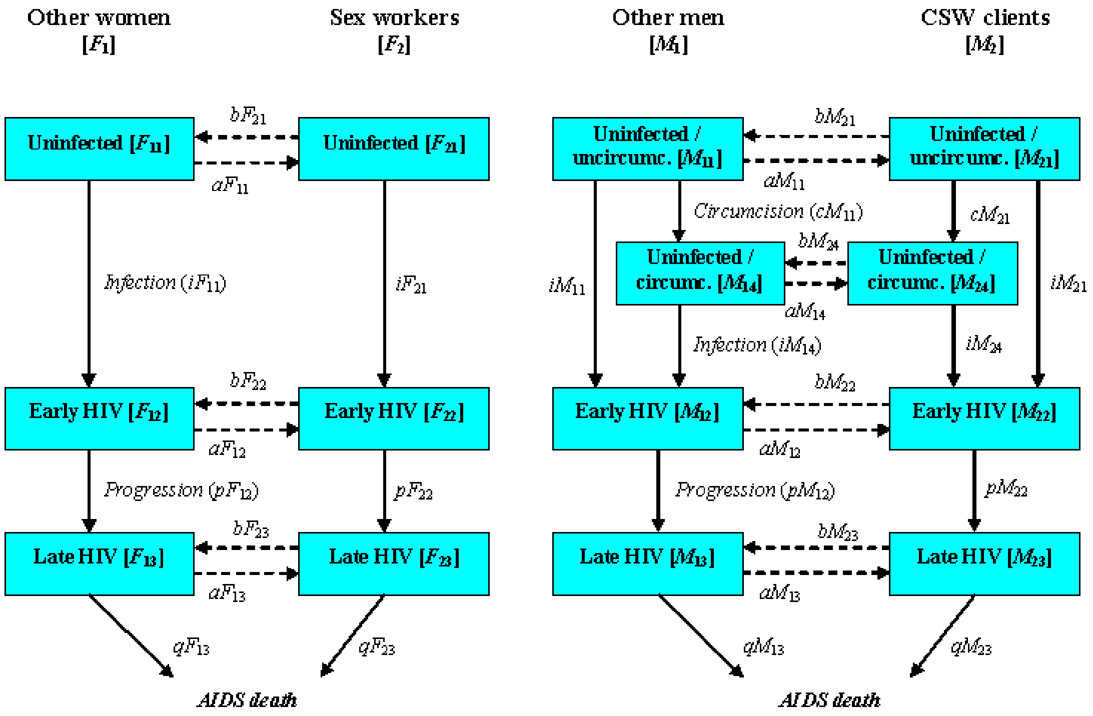


**Structure of the compartmental model**. Boxes represent compartments, i.e. the states males or females can be in. Arrows represent flows of individuals between compartments. High risk groups are male clients of sex workers, and female sex workers (csw). Disease progression is subdivided into 2 stages: early and late, including AIDS. Individuals (men) move to the circumcised boxes after circumcision. The flow diagram for women is similar except that there exists no circumcised compartment. Symbols refer to compartments and flows formally defined in the Additional file 1 (Compartments: M = male, F = female; first subscript: 1 = low risk group, 2 = high risk group; second subscript: 1 = uninfected, 2 = early HIV, 3 = late HIV, 4 =circumcised and uninfected. Flows: a = from low risk to high risk group, b = from high risk to low risk group, c = circumcision, i = infection, p =progression (to late stage HIV infection), q = death)

**Gray RH**

Gray, Ronald, Li, Xianbin, Kigozi, Godfrey, Serwadda, David, Nalugoda, Fred, Watya, Stephen, Reynolds, Steven, Wawer, Maria

The impact of male circumcision on HIV incidence and cost per infection prevented: a stochastic simulation model from Rakai, Uganda.

AIDS. 21(7):845-850, April 23, 2007.

**Published Abstract:**

*Objectives: To estimate the impact of male circumcision on HIV incidence, the number of procedures per HIV infection averted, and costs per infection averted.*

*Methods: A stochastic simulation model with empirically derived parameters from a cohort in Rakai, Uganda was used to estimate HIV incidence, assuming that male circumcision reduced the risks of HIV acquisition with rate ratios (RR) ranging from 0.3 to 0.6 in men, their female partners, and in both sexes combined, with circumcision coverage 0-100%. The reproductive number (R0) was also estimated. The number of HIV infections averted per circumcision was estimated from the incident cases in the absence of surgery minus the projected number of incident cases over 10 years following circumcision. The cost per procedure ($69.00) was used to estimate the cost per HIV infection averted.*

*Results: Baseline HIV incidence was 1.2/100 person-years. Male circumcision could markedly reduce HIV incidence in this population, particularly if there was preventative efficacy in both sexes. Under many scenarios, with RR <= 0.5, circumcision could reduce R0 to < 1.0 and potentially abort the epidemic. The number of surgeries per infection averted over 10 years was 19-58, and the costs per infection averted was $1269-3911, depending on the efficacy of circumcision for either or both sexes, assuming 75% service coverage. However, behavioral disinhibition could offset any benefits of circumcision.*

*Conclusion: Male circumcision could have substantial impact on the HIV epidemic and provide a cost-effective prevention strategy if benefits are not countered by behavioral disinhibition.*

The model simulated HIV transmission from an HIV-positive person to a negative partner within an HIV-discordant relationship. The parameters used in the simulation are listed in Table 1. The probability of transmission per sex act was computed first by stage of disease in the HIV-positive individuals (acute infection/early disease, latency, and advanced disease/AIDS), by age (15–24, 25–29, 30–34, and 35+ years), and by gender. Gender-specific rates of partner change were based on data from the Rakai cohort, with approximately 8% of HIV-positive women and 53% of HIV-positive men reporting multiple partnerships in a given year. Interview information on sexual networks was used to generate distributions of multiple partnerships, non-marital relationships, coital frequencies, and durations of relationships; on the basis of these distributions, HIV-positive individuals were randomly assigned a probability of such relationships. In this setting, virtually all multiple partnerships were concurrent. To estimate sexual contacts of infected persons with HIV-negative partners, the number of reported sexual partners in the model was randomly reduced by 0.14, since 14% of the infected population in Rakai was in a concordant HIV-positive relationship. (Details of model parameters can be provided on request.)

Each coital act was then simulated to determine whether the HIV-negative partner seroconverted, based on the transmission probability per sex act and characteristics of the couple. Partners who remained uninfected were recycled into the pool of uninfected persons and exposed to risk in subsequent replications. New seroconverters generated by the simulation were recycled into the pool of HIV-infected persons, and transmissions to their subsequent HIV-negative partners were simulated as described above. Simulations were run using SAS (version 8; SAS Institute, Cary North Carolina, USA) with 500 replications.

The total number of seroconversions and total person-years at risk among HIV-negative persons were obtained from the simulation for calculation of average HIV incidence rates per 100 person-years in the Rakai population. To assess the simulated effect of circumcision on the future course of the HIV epidemic, the approximate basic reproductive number ($R_0$) was estimated using the equation $R_0$ = [gamma]Dc [7], where [gamma] is the probability of HIV transmission per sex act, D is the total number of coital acts during the infectious period (approximately 10 years, with an average of 106.8 acts of intercourse per year per couple), and c is the average number of HIV-negative partners for each infected individual (the mean value of c in Rakai is 1.25 HIV-negative partners per HIV-positive individual per year for both sexes combined).

To estimate the possible effects of male circumcision, it was assumed that circumcision might reduce the incidence of HIV with incidence rate ratios (IRR) varying from 0.3 to 0.6 in either males or females alone, or both sexes combined. Efficacy, the reduced incidence of HIV afforded by circumcision, was estimated from 1 - IRR. These ranges of potential IRR and efficacy are comparable to those reported in the literature [1–6]. It was assumed that the coverage of circumcision services might vary from 0 to 100% of HIV-negative uncircumcised men, and simulations were run for quartiles of program coverage (25%, 50%, 75%, and 100%). Uncircumcised men constitute 84% of the Rakai male population, so coverage only pertained to this proportion. From Rakai data in male HIV-positive/female HIV-negative couples, a non-significant decreased risk of female HIV acquisition was previously observed, with a rate ratio of 0.4, comparable to the South African trial effects in men [2]; however, in an updated analysis, the HIV IRR in uninfected females with circumcised versus uncircumcised HIV-positive male partners was 0.7 [10]. Therefore, a scenario was also simulated in which the male rate ratio was 0.4 (comparable to the South African trial), and the female IRR was 0.7.

The number of HIV infections potentially averted by each surgery was calculated from the total number of incident cases expected in the population in the absence of a circumcision program minus the number of incident cases estimated with varying circumcision efficacies. To simplify presentation, it was assumed that program coverage was 75% of eligible uncircumcised men. The cost per surgery in the Rakai trial was $69.0, including postoperative care. This was used to estimate the cost per HIV infection averted by circumcision over a period of 10 years.

If circumcision reduces HIV risk, it is possible that individuals will develop exaggerated beliefs in the protective effects and increase sexual risk behaviors. Therefore, models were constructed in which number of sexual partners per individual was randomly increased, so that the average number of partners increased by 25%, 50%, or 100% in the population. The impact on HIV incidence was then compared with that estimated from the observed number of partners in the Rakai population.

**Alsallaq et al**

R. A. Alsallaq, B. Cash, H. A. Weiss, I. M. Longini, S. B. Omer, M. J. Wawer, R. H. Gray and L. J. Abu-Raddad (2008) *Quantitative assessment of the role of male circumcision in HIV epidemiology at the population level. Epidemics under review.*


**Published Abstract:**

***Background:*** *Three recent randomized trials have shown that male circumcision (MC) reduces HIV incidence in heterosexual men by about 60%. Mathematical models are needed to assess the historical role of MC in the observed disparate levels of prevalence in sub-Saharan Africa and to translate these findings into estimates of the population-level impact of MC on HIV prevalence.*

***Methods and findings****: A deterministic compartmental model of HIV dynamics with MC was parameterized by empirical data from the Rakai, Masaka and Four-City studies. The model predicts that in Kisumu, Kenya, and in Rakai, Uganda, universal MC implemented in 2008 would reduce HIV prevalence by 19%, and 14% respectively by 2020, and would avert more than 10,000 and 8,000 new infections respectively. In Kisumu, a setting with high HIV prevalence, about six MCs would be needed for each infection averted while in Rakai, eleven MCs would be needed. Females will also benefit from MC with a substantial reduction in prevalence of about 8% in Kisumu and 4% in Rakai within a few years of universal MC. The beneficial impact of MC for both males and females will not be undermined by risk behavior compensation unless the increase in risk behavior is in excess of 20%. The effectiveness of MC as an intervention is maximized by universal MC within 2-3 years.*

***Conclusion****: In West Africa, MC may have “quarantined” the spread of HIV by limiting sustainable transmission to within high risk groups and bridge populations. Our findings indicate that MC is an effective intervention in both high and intermediate HIV prevalence settings. MC coverage should be expanded as soon as possible to optimize the epidemiological impact.*

## Supporting Online Material

# The impact of male circumcision as an HIV prevention intervention in sub-Saharan Africa

Ramzi A. Alsallaq[1], Brianna Cash[2], Helen A. Weiss[3], Ira M. Longini[4,5], Jr., Saad B. Omer[6] ,Maria J. Wawer[6], Ronald H. Gray[6], and Laith J. Abu-Raddad[1]

[1]*Vaccine and Infectious Disease Institute, Program in Biostatistics and Biomathematics, Fred Hutchinson Cancer Research Center, Seattle, Washington, United States of America*
[2]*Disaster Round Table, The National Academies of Sciences, Washington, DC, United States of America*
*Fred Hutchinson Cancer Research Center, Seattle, Washington, United States of America*
[3] *Infectious Diseases Epidemiology Unit, Department of Epidemiology and Population Health, London, School of Hygiene and Tropical Medicine, London, United Kingdom*
[4]*Program in Biostatistics and Biomathematics, Fred Hutchinson Cancer Research Center, Seattle, Washington, United States of America*
[5]*Department of Biostatistics, University of Washington, Seattle, Washington, United States of America*
[6]*Johns Hopkins University, Bloomberg School of Public Health, Baltimore, Maryland, United States of America*

## Key results

We list here key results presented at the meeting of the UNAIDS Expert Group on Modelling the Impact and Cost of Male Circumcision for HIV Risk Reduction, March 2008, London, United Kingdom.

 

**Figure 1** Impact of uptake rate on outcome of MC intervention. The annual decline in HIV incidence as the male circumcision intervention is being rolled out slowly over 10 years (**A**), or rapidly over two years (**B**) in Kisumu, Kenya. Rapid rollout is much more beneficial in achieving substantial declines in incidence rapidly.

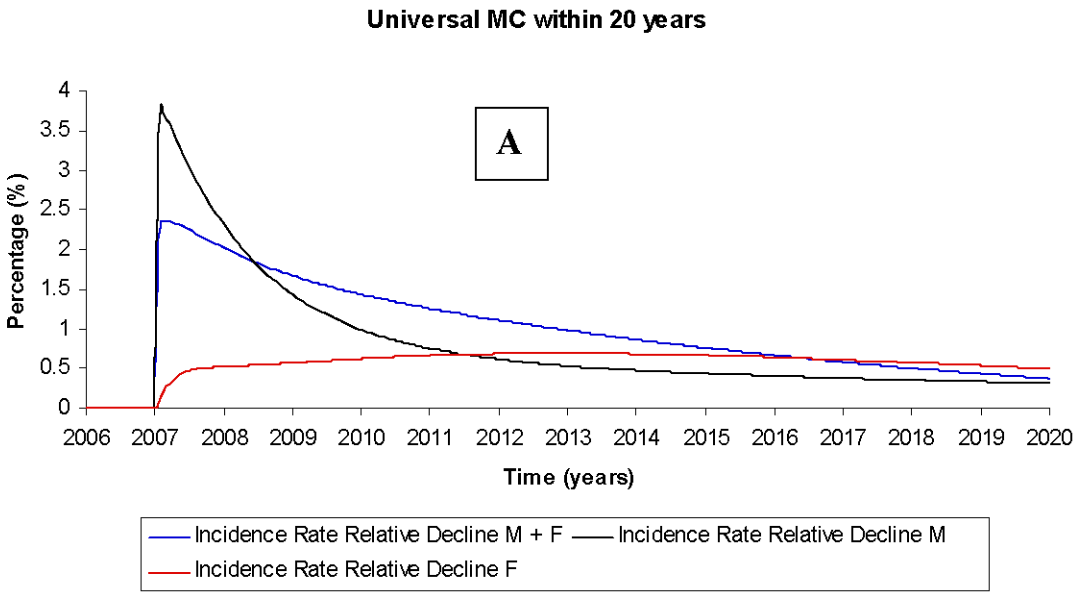


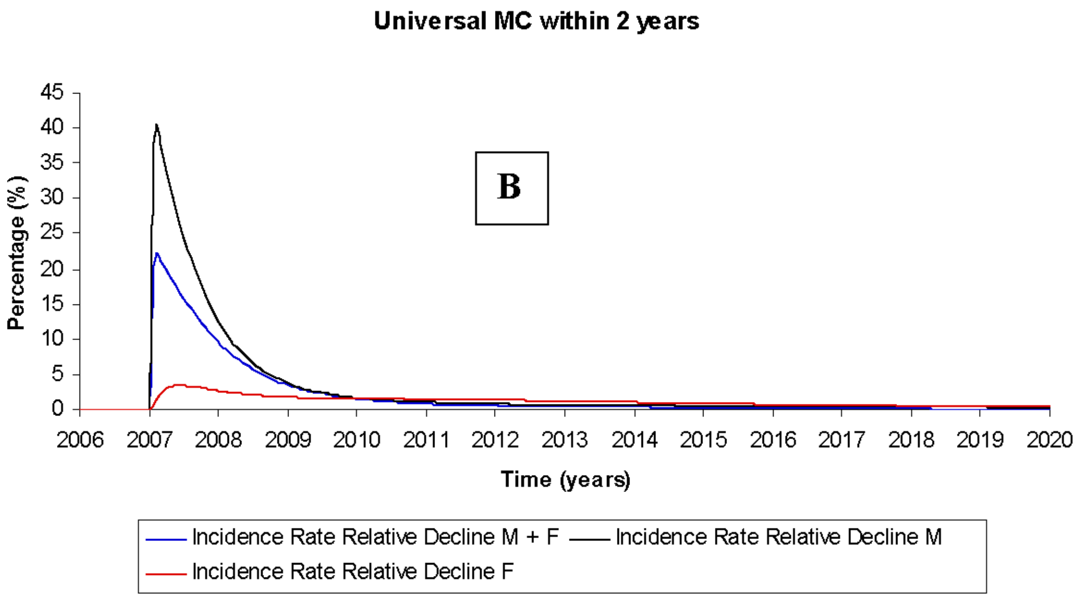

**Figure 2** Number of MC procedures needed per each HIV infection averted at varying rates of MC uptake.

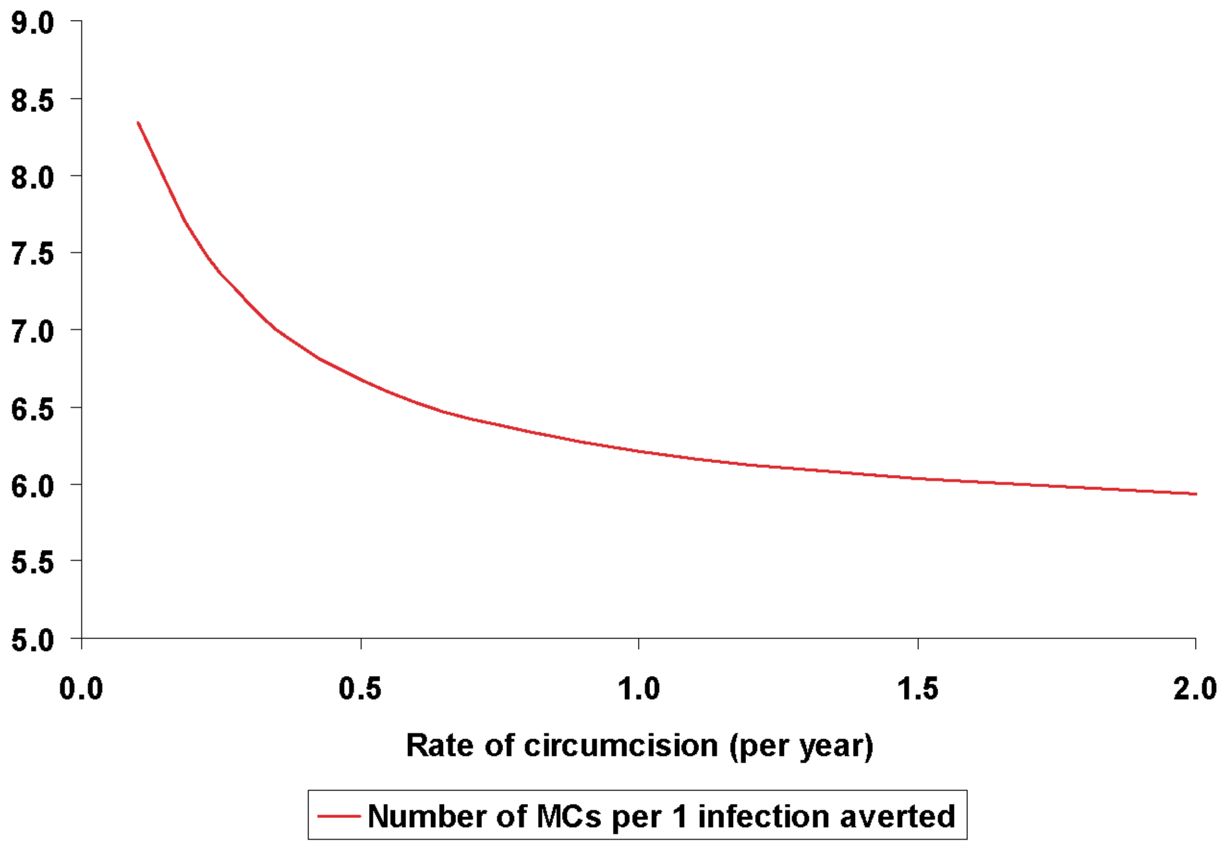

## Mathematical model description

Our model consists of a system of 12 differential equations for each risk group:

$$
\begin{aligned}
\frac{dX_f(i)}{dt} &= \mu N_{f0}(i) - \mu X_f(i) - \Lambda^{X_f(i)} X_f(i) \\
\frac{dY_f(1,i)}{dt} &= \Lambda^{X_f(i)} X_f(i) - \mu Y_f(1,i) - \omega_1 Y_f(1,i) \\
\frac{dY_f(2,i)}{dt} &= \omega_1 Y_f(1,i) - \mu Y_f(2,i) - \omega_2 Y_f(2,i) \\
\frac{dY_f(3,i)}{dt} &= \omega_2 Y_f(2,i) - \mu Y_f(3,i) - \omega_3 Y_f(3,i) \\
\\
\frac{dX_{nm}(i)}{dt} &= (1-f)\mu N_{m0}(i) - \mu X_{nm}(i) - \Lambda^{X_{nm}(i)} X_{nm}(i) - \eta X_{nm}(i) \\
\frac{dY_{nm}(1,i)}{dt} &= \Lambda^{X_{nm}(i)} X_{nm}(i) - \mu Y_{nm}(1,i) - \omega_1 Y_{nm}(1,i) \\
\frac{dY_{nm}(2,i)}{dt} &= \omega_1 Y_{nm}(1,i) - \mu Y_{nm}(2,i) - \omega_2 Y_{nm}(2,i) \\
\frac{dY_{nm}(3,i)}{dt} &= \omega_2 Y_{nm}(2,i) - \mu Y_{nm}(3,i) - \omega_3 Y_{nm}(3,i) \\
\\
\frac{dX_{cm}(i)}{dt} &= f_{cm}\mu N_{m0}(i) - \mu X_{cm}(i) - \Lambda^{X_{cm}(i)} X_{cm}(i) + \eta X_{nm}(i) \\
\frac{dY_{cm}(1,i)}{dt} &= \Lambda^{X_{cm}(i)} X_{cm}(i) - \mu Y_{cm}(1,i) - \omega_1 Y_{cm}(1,i) \\
\frac{dY_{cm}(2,i)}{dt} &= \omega_1 Y_{cm}(1,i) - \mu Y_{cm}(2,i) - \omega_2 Y_{cm}(2,i) \\
\frac{dY_{cm}(3,i)}{dt} &= \omega_2 Y_{cm}(2,i) - \mu Y_{cm}(3,i) - \omega_3 Y_{cm}(3,i)
\end{aligned}
\tag{0.1}
$$

The index $i$ stands for an $i$-sexual risk population where $i = 1, 2, 3, 4$ represent low, low to intermediate, intermediate to high, and high risk groups respectively. The population is stratified into three groups based on sex and male circumcision (MC) status: females,

non-circumcised males, and circumcised males (subscripts $f$, $nm$, and $cm$, respectively). Here, $X_\gamma(i)$ is the HIV susceptible population belonging to sex and MC group $\gamma$. The $Y_\gamma(\alpha,i)$ are the HIV infected populations where the index $\alpha$ marks the stage of HIV pathogenesis; $\alpha = 1,2,3$ stand for acute, latent, and late stages, respectively. The $N_f(i)$, $N_m(i)$ are the female and male population sizes of each $i$-risk group respectively, and $N_{f0}(i)$, $N_{m0}(i)$ are the corresponding initial population sizes. Lastly, $f_{cm}$ is the fraction of the male population that are circumcised before starting sexual activity.

The progression of HIV is described by $\omega_1$, the rate of progression from acute to latent stage, $\omega_2$, the rate from latent to late stage, and $\omega_3$, the rate of HIV/AIDS disease mortality. $\mu$ is the birth (and death) rate. We assume a constant birth rate in the model and we do not stratify the population explicitly according to age. $\eta$ denotes the average rate at which non-circumcised males are being circumcised as part of a MC intervention. The reciprocal (i.e. $\eta^{-1}$) provides the average waiting time before circumcision. The rates $\Lambda^{X_\gamma(i)}$ are the HIV forces of infection (hazard rates of infection) experienced by each susceptible population $X_\gamma(i)$:

$$\Lambda^{X_{nm}(i)} = \rho_{X_{nm}(i)} \sum_{j=1,2,3,4} \sum_{\alpha=1,2,3} \mathcal{G}_{f\to m}(i,j) \frac{t_{Y_f(\alpha,j)\to X_{nm}(i)} \rho_{Y_f(\alpha,j)} Y_f(\alpha,j)}{\rho_{X_f(j)} X_f(j) + \sum_{\alpha'=1,2,3} \rho_{Y_f(\alpha',j)} Y_f(\alpha',j)}$$

$$\Lambda^{X_{cm}(i)} = (1-q)(1+r)\Lambda^{X_{nm}(i)} \qquad (0.2)$$

$$\Lambda^{X_f(i)} = \rho_{X_f(i)} \sum_{j=1,2,3,4} \sum_{\alpha=1,2,3} \mathcal{G}_{m\to f}(i,j)$$

$$\left\{ \frac{\rho_{Y_{nm}(\alpha,j)} t_{Y_{nm}(\alpha,j)\to X_f(i)} Y_{nm}(\alpha,j)}{\rho_{X_{nm}(j)} X_{nm}(j) + (1+r)\rho_{X_{nm}(j)} X_{cm}(j) + \sum_{\alpha'=1,2,3} \left[ \rho_{Y_{nm}(\alpha',j)} Y_{nm}(\alpha',j) + (1+r)\rho_{Y_{nm}(\alpha',j)} Y_{cm}(\alpha',j) \right]} \right.$$

$$\left. + \frac{(1+r)\rho_{Y_{nm}(\alpha,j)} t_{Y_{cm}(\alpha,j)\to X_f(i)} Y_{cm}(\alpha,j)}{\rho_{X_{nm}(j)} X_{nm}(j) + (1+r)\rho_{X_{nm}(j)} X_{cm}(j) + \sum_{\alpha'=1,2,3} \left[ \rho_{Y_{nm}(\alpha',j)} Y_{nm}(\alpha',j) + (1+r)\rho_{Y_{nm}(\alpha',j)} Y_{cm}(\alpha',j) \right]} \right\}$$

In these expressions, $\rho_{Z(i)}$ describes the *effective* new sexual partner acquisition rate for each population variable $Z(i)$. Note that we use the term effective rate of partner change, as opposed to rate of partner change, since this parameter does not merely reflect the actual rate at which individuals change their partners, but also represents other behavioral mechanisms that effectively enhance this quantity such as concurrency and topology of sexual networks (Watts and May 1992; Kretzschmar and Morris 1996; Morris 1997), as well as variability in risk behavior (May and Anderson 1988). The parameter $r \in [0,\infty)$ models the relative increase in the effective rate of partner change due to behavioral dis-inhibition experienced by circumcised males following circumcision. On the other hand, the parameter $q \in [0,1]$ measures the efficacy of male circumcision against HIV acquisition (Auvert, Taljaard et al. 2005; Gray, Kigozi et al. 2007; Nagelkerke, Moses et al. 2007). At the extremes, $q = 0$ implies no protection against HIV and $q = 1$ implies total protection against HIV.

The parameters $t_{Y_\gamma(\alpha,i)\to X_{\gamma'}(j)}$ stand for HIV transmission probability per partnership in a heterosexual partnership between a member of the susceptible population $X_{\gamma'}(j)$ and a member of the HIV infected population $Y_\gamma(\alpha,i)$. These transmission probabilities are expressed in terms of HIV transmission probability per coital act per HIV stage in this partnership ( $p^{HIV}_{Y_\gamma(\alpha,i)\to X_{\gamma'}(j)}$), the frequency of coital acts per HIV stage in this partnership

($n_{Y_\gamma(\alpha,i)\leftrightarrow X_{\gamma'}(j)}$), and the duration ($\tau_{Y_\gamma(\alpha,i)\leftrightarrow X_{\gamma'}(j)}$) of this partnership, using the binomial model

$$t_{Y_\gamma(\alpha,i)\to X_{\gamma'}(j)} = 1-\left(1-p^{HIV}_{Y_\gamma(\alpha,i)\to X_{\gamma'}(j)}\right)^{n_{Y_\gamma(\alpha,i)\leftrightarrow X_{\gamma'}(j)}\tau_{Y_\gamma(\alpha,i)\leftrightarrow X_{\gamma'}(j)}} \quad (0.3)$$

The best data on HIV transmission probability per coital act indicates that there are no differences between HIV transmission probability per coital act from a non-circumcised male to a female and from a female to a non-circumcised male (Gray, Wawer et al. 2001; Wawer, Gray et al. 2005). For completeness, we included in our model a reduction factor $g$ in the transmission probability per coital act from an infected circumcised male to a susceptible female, but we set it to zero in our results due to the absence of a concrete evidence for this effect. Thus,

$$p^{HIV}_{Y_{cm}(\alpha,i)\to X_f(j)} = (1-g)\times p^{HIV}_{Y_{nm}(\alpha,i)\to X_f(j)} \quad (0.4)$$

The mixing among the four risk groups between females and males is dictated by the sexual-mixing matrices $\mathcal{G}_{a\to b}(i,j)$ which provides the probability that an individual of gender $a$ in risk group $j$ would choose a partner of gender $b$ in risk group $i$ (Garnett and Anderson 1993). The two mixing matrices are given by the expressions

$$\mathcal{G}_{m\to f}(i,j) = e\delta_{i,j} + (1-e)\frac{\tilde{\rho}_m(j)}{\sum_{k=1,2,3,4}\tilde{\rho}_m(k)}$$

$$\mathcal{G}_{f\to m}(i,j) = e'\delta_{i,j} + (1-e')\frac{\tilde{\rho}_f(j)}{\sum_{k=1,2,3,4}\tilde{\rho}_f(k)} \quad (0.5)$$

Here, $\delta_{i,j}$ is the identity matrix and the parameters $e,\ e' \in [0,1]$ measures the degree of assortativeness in the mixing. At the extreme $e = e' = 0$, the mixing is fully proportional while at the other extreme $e = e' = 1$, the mixing is fully assortative as individuals choose partners only from within their risk group. $\tilde{\rho}_m(j)$ represents the total number of partnerships acquired by males of risk group $j$ regardless of their circumcision or HIV infection status and is given by

$$\begin{aligned}\tilde{\rho}_m(j) = \rho_{X_{nm}(j)} X_{nm}(j) + (1+r)\,\rho_{X_{nm}(j)} X_{cm}(j) + \\ \sum_{\alpha'=1,2,3} \left( \rho_{Y_{nm}(\alpha',j)} Y_{nm}(\alpha',j) + (1+r)\,\rho_{Y_{nm}(\alpha',j)} Y_{cm}(\alpha',j) \right)\end{aligned} \qquad (0.6)$$

Similarly the total number of partnerships acquired by females of risk group $i$ regardless of their circumcision or HIV infection status is given by

$$\tilde{\rho}_f(i) = \rho_{X_f(i)} X_f(i) + \sum_{\alpha=1,2,3} \rho_{Y_f(\alpha,i)} Y_f(\alpha,i) \qquad (0.7)$$

We assume a balance in partnerships so that the number of partnerships formed by males is equal to the number of partnerships made by females. This is done by constraining the number of partnerships formed by females in any specific population compartment with males in a certain population compartment to be equal to the number of partnerships formed by males in this certain compartment with the females in that specific compartment. For example, the mixing between the two compartments $Y_{cm}(\alpha,i)$ and $X_f(j)$ is balanced through the condition

$$\rho_{X_f(i)} X_f(i) \mathcal{G}_{m\to f}(i,j) \frac{(1+r)\,\rho_{Y_{nm}(\alpha,j)} Y_{cm}(\alpha,j)}{\tilde{\rho}_m(j)} = (1+r)\,\rho_{Y_{nm}(\alpha,j)} Y_{cm}(\alpha,j) \mathcal{G}_{f\to m}(j,i) \frac{\rho_{X_f(i)} X_f(i)}{\tilde{\rho}_f(i)} \qquad (0.8)$$

Similar conditions are established between all population compartments leading consistently to the balance equation

$$\tilde{\rho}_m(j)\mathcal{G}_{f\to m}(j,i) = \tilde{\rho}_f(i)\mathcal{G}_{m\to f}(i,j) \quad (0.9)$$

This equation defines an expression for $\mathcal{G}_{f\to m}(i,j)$ in terms of $\mathcal{G}_{m\to f}(j,i)$. $\tilde{\rho}_f(i)$ can be written in terms of $\tilde{\rho}_m(i)$ by summing over risk group $j$ in (0.9), using (0.5) and matrix inversion to yield

$$\tilde{\rho}_f(i) = \sum_{j=1,2,3,4}[\mathcal{G}^T_{m\to f}]^{-1}_{(i,j)}\tilde{\rho}_m(j) = \tilde{\rho}_m(i) \quad (0.10)$$

and

$$\mathcal{G}_{f\to m}(i,j) = \mathcal{G}_{m\to f}(i,j) \quad (0.11)$$

These equations determine the partner change rates of one of the sexes if those of the other sex are determined.

For Kisumu, Kenya, we used the general population survey of the Four City study (Buve, Carael et al. 2001) to fit HIV prevalence levels in the year 1997-1998 for the sexually active population. Meanwhile, we used the antenatal clinic surveillance data (WHO/AFRO 2002; UNAIDS/WHO 2004) to fit the time series trends in HIV prevalence. For Rakai, Uganda, we used data from the Rakai group survey (Gray 2008). Please note that antenatal surveillance data do not necessarily reflect the HIV population prevalence level (UNAIDS/WHO 2003), though they are valuable in describing the trends in prevalence. Our prediction for HIV prevalence in Kisumu appears to underestimate the epidemic since the general population survey for Kisumu found substantially lower prevalence than provided by the antenatal surveillance data.

## Sensitivity and uncertainty analyses

We performed sensitivity and uncertainty analyses to assess the robustness of our predictions to the uncertainty in the MC efficacy, behavioral parameters, and HIV progression parameters used to parameterize the model.

The parameter values in our model such as HIV transmission probabilities, HIV stage durations, and MC efficacy are set by empirical data and one of the main goals of this work is to assess the implications of these measured values on the impact of MC as an intervention in sub-Saharan Africa. We examined the sensitivity of our MC intervention predictions to variations of 1) 43-69% in the MC efficacy (efficacy is 58% (95% CI 43-69%) according to a random-effect meta-analysis of results of the three MC trials (Weiss, Halperin et al. 2008)); 2) 15% in the duration from onset of HIV infection to death; 3) 15% in the fraction of the population in the highest risk group with a corresponding variation in the rest of the risk groups; 4) 15% in all values of the new sexual partner acquisition rates; 5) 15% in the degree of assortativeness in the mixing between the risk groups.

Figure 3 shows the results of the sensitivity and uncertainty analyses with respect to our predictions for the epidemiologic measures at 2020. The analyses were done by Monte Carlo sampling from the specified ranges of uncertainty using the uniform distribution for 1000 runs of the model. The predictions are largely invariable to the specified variations in the fraction of the population in the highest risk group, the rates of partner change, the level of assortativeness in the mixing between the risk groups, and the duration from onset of infection to death. However, not surprisingly, the epidemiologic measures are

sensitive to the variations in the MC efficacy and vary by about 15-20% with the specified range of uncertainty in the MC efficacy.

**Figure 3** Sensitivity and uncertainty analyses with respect to MC efficacy, behavioural parameters, and HIV progression parameters. The impact of universal MC intervention with respect to variations in (**A**) MC efficacy; (**B**) fraction of the population in the highest risk group; (**C**) new sexual partner acquisition rates; (**D**) assortativeness in the mixing between the risk groups; (**E**) duration from onset of HIV infection to death.

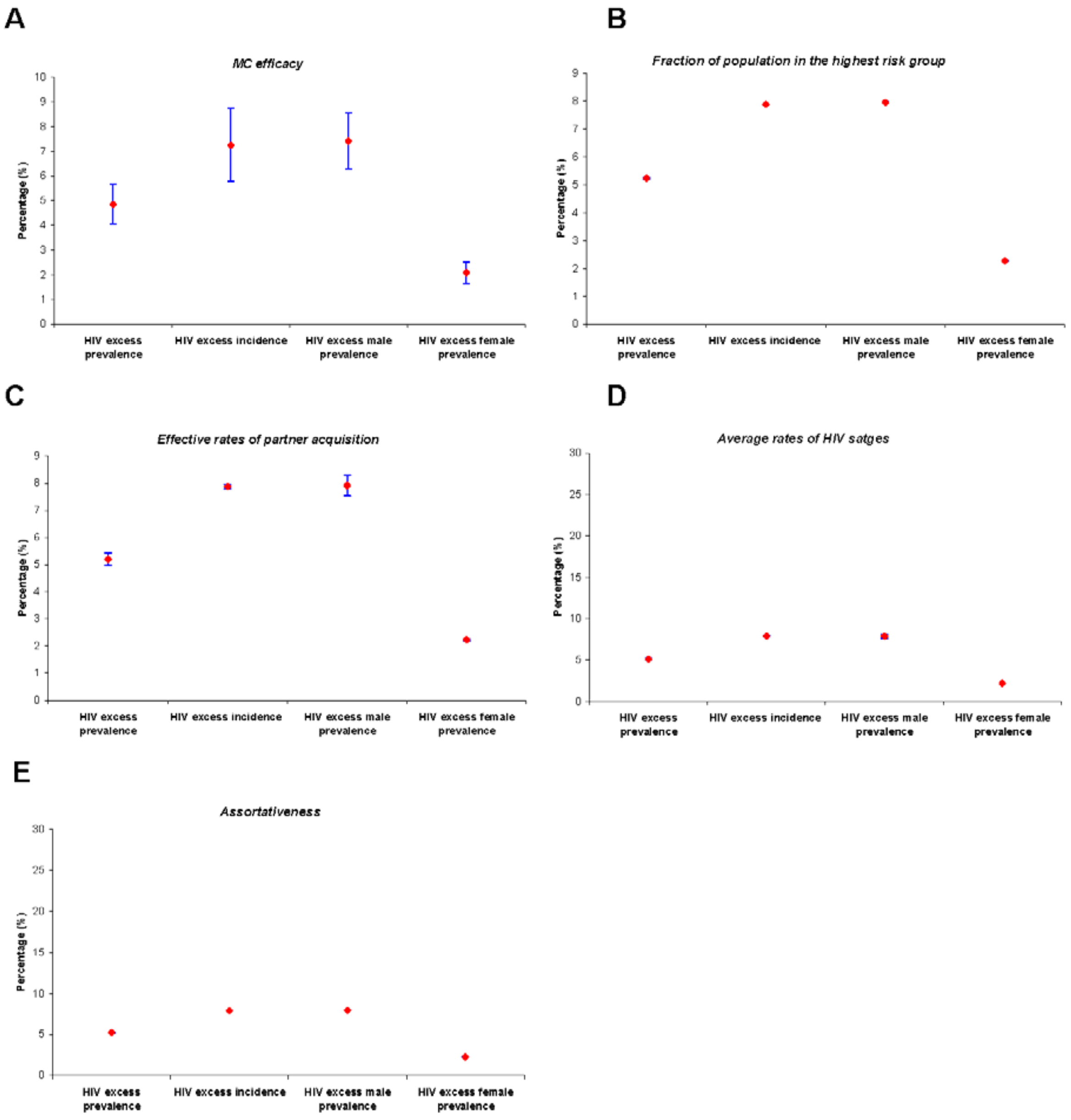

## References


Auvert, B., D. Taljaard, et al. (2005). "Randomized, controlled intervention trial of male circumcision for reduction of HIV infection risk: the ANRS 1265 Trial." PLoS Med **2**(11): e298.

Buve, A., M. Carael, et al. (2001). "Multicentre study on factors determining differences in rate of spread of HIV in sub-Saharan Africa: methods and prevalence of HIV infection." Aids **15 Suppl 4**: S5-14.

Garnett, G. P. and R. M. Anderson (1993). "Factors controlling the spread of HIV in heterosexual communities in developing countries: patterns of mixing between different age and sexual activity classes." Philos Trans R Soc Lond B Biol Sci **342**(1300): 137-59.

Gray, R. H. (2008). "Personal communication."

Gray, R. H., G. Kigozi, et al. (2007). "Male circumcision for HIV prevention in men in Rakai, Uganda: a randomised trial." The Lancet **369**(9562): 657-666.

Gray, R. H., M. J. Wawer, et al. (2001). "Probability of HIV-1 transmission per coital act in monogamous, heterosexual, HIV-1-discordant couples in Rakai, Uganda." Lancet **357**(9263): 1149-53.

Kretzschmar, M. and M. Morris (1996). "Measures of concurrency in networks and the spread of infectious disease." Mathematical Biosciences **133**(2): 165-195.

May, R. M. and R. M. Anderson (1988). "The Transmission Dynamics of Human Immunodeficiency Virus (Hiv)." Philosophical Transactions of the Royal Society of London Series B-Biological Sciences **321**(1207): 565-607.

Morris, M. (1997). "Sexual networks and HIV." Aids **11**: S209-S216.

Nagelkerke, N. J. D., S. Moses, et al. (2007). "Modelling the public health impact of male circumcision for HIV prevention in high prevalence areas in Africa." Bmc Infectious Diseases **7**: -.

UNAIDS/WHO (2003). "Reconciling antenatal clinic-based surveillance and population-based survey estimates of HIV prevalence in sub-Saharan Africa."

UNAIDS/WHO (2004). "AIDS epidemic update 2004."

Watts, C. H. and R. M. May (1992). "The influence of concurrent partnerships on the dynamics of HIV/AIDS." Math Biosci **108**(1): 89-104.

Wawer, M. J., R. H. Gray, et al. (2005). "Rates of HIV-1 transmission per coital act, by stage of HIV-1 infection, in Rakai, Uganda." J Infect Dis **191**(9): 1403-9.

Weiss, H. A., D. Halperin, et al. (2008). "Male circumcision for HIV prevention: from evidence to action?" AIDS **22**(5): 567-74.

WHO/AFRO (2002). "HIV/AIDS Epidemiological Surveillance Update for the WHO African Region 2002 Country Profiles."

**Williams *et al.***

Williams BG, Lloyd-Smith JO, Gouws E, Hankins C, Getz WM, et al. (2006) *The potential impact of male circumcision on HIV in Sub-Saharan Africa.* PLoS Med 3: e262.

**Published Abstract:**

***Background:*** *A randomized controlled trial (RCT) has shown that male circumcision (MC) reduces sexual transmission of HIV from women to men by 60% (32%-76%; 95% CI) offering an intervention of proven efficacy for reducing the sexual spread of HIV. We explore the implications of this finding for the promotion of MC as a public health intervention to control HIV in sub-Saharan Africa.*

***Methods and Findings:*** *Using dynamical simulation models we consider the impact of MC on the relative prevalence of HIV in men and women and in circumcised and uncircumcised men. Using country level data on HIV prevalence and MC, we estimate the impact of increasing MC coverage on HIV incidence, HIV prevalence, and HIV-related deaths over the next ten, twenty, and thirty years in sub-Saharan Africa. Assuming that full coverage of MC is achieved over the next ten years, we consider three scenarios in which the reduction in transmission is given by the best estimate and the upper and lower 95% confidence limits of the reduction in transmission observed in the RCT. MC could avert 2.0 (1.1-3.8) million new HIV infections and 0.3 (0.1-0.5) million deaths over the next ten years in sub-Saharan Africa. In the ten years after that, it could avert a further 3.7 (1.9-7.5) million new HIV infections and 2.7 (1.5-5.3) million deaths, with about one quarter of all the incident cases prevented and the deaths averted occurring in South Africa. We show that a) MC will increase the proportion of infected people who are women from about 52% to 58%; b) where there is homogenous mixing but not all men are circumcised, the prevalence of infection in circumcised men is likely to be about 80% of that in uncircumcised men; c) MC is equivalent to an intervention, such as a vaccine or increased condom use, that reduces transmission in both directions by 37%.*

***Conclusions:*** *This analysis is based on the result of just one RCT, but if the results of that trial are confirmed we suggest that MC could substantially reduce the burden of HIV in Africa, especially in southern Africa where the prevalence of MC is low and the prevalence of HIV is high. While the protective benefit to HIV-negative men will be immediate, the full impact of MC on HIV-related illness and death will only be apparent in ten to twenty years..*

# Male circumcision and HIV in Africa

## Mathematical note

The formal definition of a convolution (main text; Equation 14) is

$$A(t)\otimes B(t)\equiv\int_{-\infty}^{+\infty}A(\tilde{t})B(t-\tilde{t})\,d\tilde{t} \qquad 1$$

## Rate of expansion of male circumcision

We assume that the coverage of MC increases logistically with time. The analytical expression for coverage is then

$$\chi=\chi_0+(1-\chi_0)\frac{e^{\alpha(t-t_0)}}{1+e^{\alpha(t-t_0)}} \qquad 2$$

where $\chi_0$ is the initial prevalence of MC, $t_0$ is the time at which the logistic term reaches 0.5, and $\alpha$ determines the rate at which the coverage of MC increases. For the ten year expansion we set $t_0$ = 2010 and $\alpha$ = 0.6/year while for the five year expansion we set $t_0$ = 2007.5 and $\alpha$ = 1.2/year giving the coverage as a function of time, illustrated for South Africa in Figure S1.

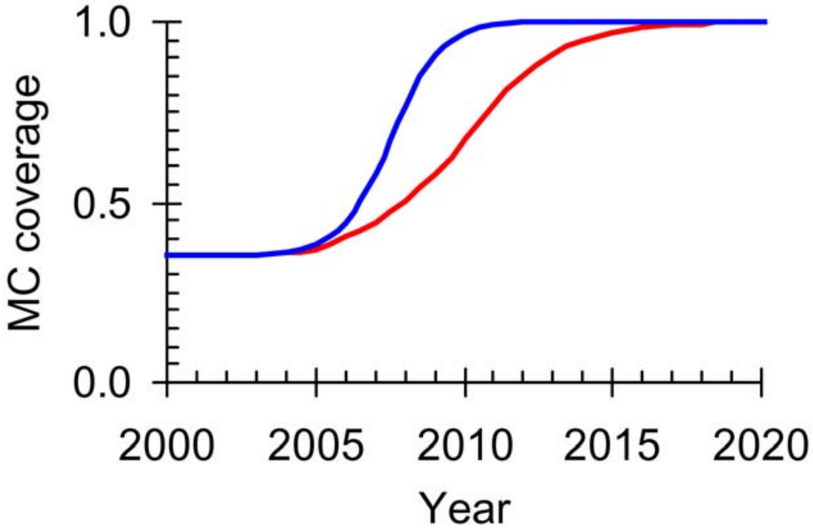


Figure S1. The increase of MC in South Africa starting from a coverage of 35% and reaching full coverage in 2015 (red line) and 2010 (blue line).

## Separating contact and transmission parameters

We focus on interventions that reduce the probability of transmission without changing sexual behaviour. The key result (Equation 6 in the main text) is that $R_0$ can be expressed as

$$R_0=f(\phi)\,g(c) \qquad 3$$

where $\phi$ depends on those parameters that determine the risk of infection given contact with an infected person and $c$ depends on those parameters that determine the number of sexual contacts per unit time. For interventions that reduce transmission probabilities while not affecting sexual behaviour, changes in the epidemic can be estimated from changes in the transmission probabilities without reference to the (unchanging) sexual behaviour. An absolute value of $R_0$ during the intervention can be estimated by multiplying the relative change by an empirical estimate of the pre-intervention $R_0$, which implicitly includes the complexities and heterogeneities of sexual behaviour.

**Random mixing**

If the intervention leads to changes in sexual behaviour, at either the individual or population level, then further analysis is required. Such scenarios have been considered in detail in previous work [1-6]. If only a fraction of the population is covered by the intervention (such as circumcision or vaccination), then the decoupling argument assumes that individuals mix randomly with respect to intervention coverage status. If interventions are targeted at specific risk groups the contact rates and probability of transmission given contact are not independent and cannot be decoupled. If higher-risk groups are targeted successfully, then results from the decoupled analysis will place a conservative lower bound on the benefits of the intervention.

**Differential impact of male circumcision on different groups**

Because of the asymmetry of HIV transmission between men and women, prevalence among women is generally higher than prevalence among men in heterosexual epidemics [7]. Higher levels of circumcision could increase this asymmetry in transmission, and increase the imbalance in prevalence between men and women. Circumcision will reduce the prevalence of infection among all men but will have a greater impact on those that are circumcised. In the main text we calculate the impact of MC averaged over women, circumcised men, and uncircumcised men, and we make separate estimates of the relative impact on men and women. Here we analyse a model that explicitly divides the population into women, circumcised men, and uncircumcised men in order to confirm the validity and test the accuracy of the results obtained in the main text. We employ a simple model formulation for analytic tractability bearing in mind that real-world complexities, including heterogeneity in risk and mixing patterns, could affect the results.

**The three-group model**

The disease process is modelled using an SI (susceptible-infected) framework. Infected individuals die at per capita rate $\delta$, but we assume constant population size for each group so that people who die are replaced immediately by susceptible people of the same type. Each individual is assumed to have heterosexual contacts at an effective contact rate $c$, and women divide their contacts among circumcised and uncircumcised men in proportion to their frequency in the population. We model transmission using frequency-dependent incidence, such that the incidence rate in each group is the product of the number of susceptible people

in the group, their contact rate with members of other groups, the probability of infection given contact with members of other groups, and the prevalence of infection in the other groups. This model is a mechanistically accurate depiction of partnership-based HIV transmission, particularly for populations with frequent partner change [8].

Let $S_j$ and $I_j$ be the numbers of susceptible and infected individuals in each population group where $j = u$, $c$ or $f$ represent uncircumcised men, circumcised men, and women, respectively, and let $N_j = S_j + I_j$ be the total number in each group. The three-group model can then be written:

$$\frac{dS_u}{dt} = \delta I_u - c\phi_m S_u \frac{I_f}{N_f} \tag{4}$$

$$\frac{dI_u}{dt} = c\phi_m S_u \frac{I_f}{N_f} - \delta I_u \tag{5}$$

$$\frac{dS_c}{dt} = \delta I_c - c\phi_m (1-\pi_m) S_c \frac{I_f}{N_f} \tag{6}$$

$$\frac{dI_c}{dt} = c\phi_m (1-\pi_m) S_c \frac{I_f}{N_f} - \delta I_c \tag{7}$$

$$\frac{dS_f}{dt} = \delta I_f - c\phi_f Sf\left( (1-\chi)\frac{I_u}{N_u} + \chi\left(1-\pi_f\right)\frac{I_c}{N_c} \right) \tag{8}$$

$$\frac{dI_f}{dt} = c\phi_f Sf\left( (1-\chi)\frac{I_u}{N_u} + \chi\left(1-\pi_f\right)\frac{I_c}{N_c} \right) - \delta I_f \tag{9}$$

Here $\delta$ is the disease-induced mortality, $c$ is the effective contact rate, $\chi$ is the proportion of men that are circumcised, $\phi_m$ is the probability per contact of female-to-male transmission, and $\pi_m$ is the proportional reduction in this probability if a man is circumcised; $\phi_f$ and $\pi_f$ are the corresponding quantities for male-to-female transmission.

To simplify the model, we convert the equations to proportions, introducing new state variables $i_u = I_u / N_u$, $i_c = I_c / N_c$, and $i_f = I_f / N_f$. The three-group model is then represented by the following system of equations:

$$\frac{di_u}{dt} = c\phi_m (1-i_u) i_f - \delta i_u \tag{10}$$

$$\frac{di_c}{dt} = c\phi_m (1-i_c)(1-\pi_m) i_f - \delta i_c \tag{11}$$

$$\frac{di_f}{dt} = c\phi_f (1-i_f)\left((1-\chi) i_u + \chi\left(1-\pi_f\right) i_c\right) - \delta i_f \tag{12}$$

We found the endemic equilibrium of this system using *Mathematica 5.0* (Wolfram Research, Champaign IL), and calculated relative measures of the long-term burden of HIV in different groups under different circumcision scenarios. The resulting expressions are too complex for direct interpretation, but are available from the authors upon request. The proportion of prevalent cases that are women agrees precisely with Equation 7 of the main text (derived from a simpler two-group model) when $\chi = 0$ or 1, and differs by less than 1.3% at intermediate MC coverage.

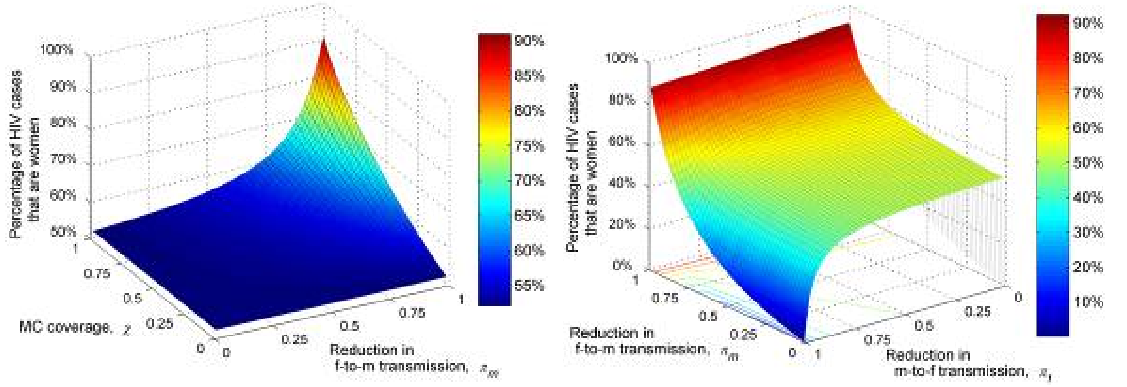


Figure S2. The percentage of all HIV cases that occur in women, as a function of circumcision parameters. In (a) circumcision is assumed to have no protective benefit for women ($\pi_f = 0$). In (b), circumcision coverage is 100% ($\chi = 1$). Other parameters were chosen with reference to the South African HIV epidemic as discussed in the main text: $\delta = 0.102\ \text{yr}^{-1}$, $c\phi_m = 0.52\ \text{yr}^{-1}$, $c\phi_f = 1.05\ \text{yr}^{-1}$.

**The impact of MC on men and women**

Figure S2 shows the proportion of all prevalent HIV cases that are women under two scenarios: assuming that MC has no effect on male-to-female transmission (Figure S2a), and assuming that circumcision coverage is complete but allowing for an effect on female-to-male and male-to-female transmission (Figure S2b). Figure S2a shows that the proportion of cases that are women could only increase to 70% if all men are circumcised and MC reduces female-to-male transmission by 88% or more ($\pi_m \geq 0.88$), or if at least 61% are circumcised and MC reduces female-to-male transmission by 99% ($\pi_m = 0.99$). Figure S2b shows that, with 100% MC coverage, the proportion of prevalent HIV cases that are women could fall to 40% only if MC reduced male-to-female transmission by at least 88% (if female-to-male transmission is not reduced at all), or if MC reduced male-to-female transmission by 99% and female-to-male transmission by 78% or less.

If the per contact probability of HIV transmission from uncircumcised men to women is twice that from women to uncircumcised men (i.e. $\phi_f / \phi_m = 2.0$), then in a population with no male circumcision ($\chi = 0$) we calculate that 52% of HIV cases will be women (Figure S2a). In a wholly circumcised population ($\chi = 1$), if circumcision provides 60% protection to men ($\pi_m = 0.60$) and no protection to women ($\pi_f = 0$), then the proportion of HIV cases who are women will increase to 58%. In this scenario ($\chi = 1$, $\pi_m = 0.60$), even if circumcision reduces

male-to-female transmission by 50% ($\pi_f = 0.5$), women will still comprise 55% of HIV cases (Figure S2b).

**The impact of MC on circumcised versus uncircumcised males**

Figure S3 shows the impact of MC on the ratio of the prevalence in circumcised to uncircumcised men as a function of the reduction in transmission. If circumcision reduces female-to-male transmission by 60%, HIV prevalence among circumcised men is still expected to be about 78% of that among uncircumcised men, and it is only when the reduction in transmission is greater than about 80% that the difference in the prevalence in the two groups is substantial. The chronic nature of the infection and the interconnectedness of the population both act to average out the risk in the long term. Note, however, that this prediction could change if circumcised and uncircumcised men were in different communities and sexual mixing among communities was limited.

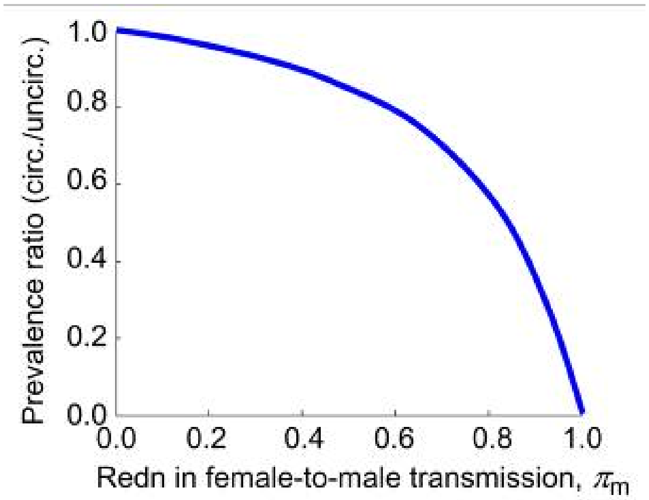


Figure S3. Ratio of the prevalence of HIV in circumcised to uncircumcised men at the steady-state assuming an average infectious period of 9.8 years. The relationship is almost completely independent of the circumcision coverage, $\chi$, and the possible protective effect for women, $\pi_f$. Other parameters: $\chi = 1$, $\pi_f = 0$, $c\phi_m = 0.52$/yr and $c\phi_f = 1.04$/yr

**Collapsing the three-group model to a one-group model**

In the main text, we fit trends and make projections based on country and regional HIV prevalence data. Because separate time series do not exist for females, uncircumcised males, and circumcised males, it is unnecessarily cumbersome to use the three-group model so we collapse the three-group model to a one-group SI model. Here we carry out simulations to determine the levels of error that may introduced by this procedure.

To represent the effects of circumcision in the collapsed model, we use the approximations discussed in the main text to replace Equations 10–12 by a single equation governing the proportion, *i*, of all individuals who are infected:

$$\frac{di}{dt} = c\sqrt{\phi_f \phi_m (1-\pi_m \chi)(1-\pi_f \chi)}(1-i)i - \delta i \qquad 13$$

To compare the two models quantitatively, we choose basic parameters to fit the data for the South African epidemic, as described in the Methods section of the main text. Fitting an exponential trend line to the South Africa prevalence data gives $r = 0.55 \pm 0.16$/year (best estimate ± standard error), which corresponds to an intrinsic doubling time of $d = 1.26 \pm 0.37$ years. The life expectancy of people infected with HIV and without access to ART, standardized to a mean age at infection of 27 years, is $\tau = 9.8 \pm 0.5$ years [9,10], so the average mortality rate is 0.102 ± 0.005/year. We then calculate $R_0 = r/\delta+1$ [11], yielding the value 6.4 ± 1.6 using Monte Carlo simulation to estimate the standard error in the result. Since an estimated $\chi = 0.35 \pm 0.10$ of South African men are circumcised (Table 1) and MC reduces female-to-male transmission by $\pi_m$=0.60 (0.32–0.76), the value of $R_0$ in the absence of circumcision would be $(r/\delta+1)/\sqrt{1-\chi\pi_m}$ or 7.2 ± 1.8. We relate this empirical estimate of $R_0$ to the expression derived from the two-sex model, $R_0 = c\sqrt{\phi_f \phi_m}/\delta$ (Equation 6 in the main text), to estimate male-to-female and female-to-male transmission rates. Taking $\phi_f/\phi_m$ to be 2.0 ± 0.5, we find $c\phi_m = 0.52 \pm 0.16$/year and $c\phi_f = 1.05 \pm 0.29$/year.

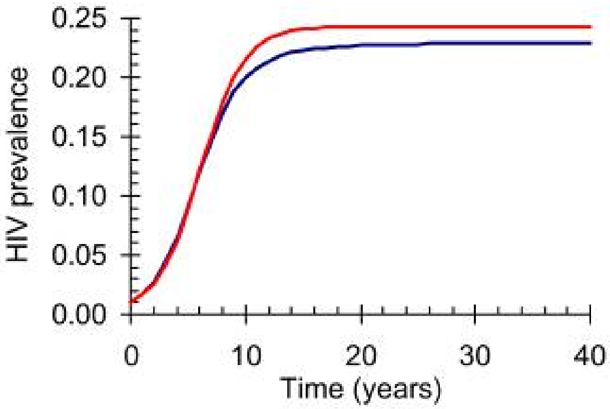


Figure S4. Comparison of three-group (blue line) and the equivalent one-group (red line) models. Parameter values: $\chi = 0.35$, $\pi_m = 0.60$, $\pi_f = 0$, $c\phi_m = 0.52$/yr and $c\phi_f = 1.04$/yr, $\delta = 0.102$/year and $\rho = 0.29$.

Finally, for this model comparison we scale the endemic prevalence using the Epidemiological Projection Package (EPP) method [12] assuming that a fraction $\gamma$ of the population is at risk for HIV infection, while the remaining $1-\gamma$ are at zero risk. For $R_0 = 6.4$ in the presence of 35% MC coverage, we choose $\gamma = 0.29$ to yield a steady state prevalence near 24.6%, the estimated steady state for South Africa (Table 1). We simulated the two models using Berkeley Madonna (Berkeley CA), and the resulting epidemic curves are shown in Figure S4.

The initial doubling times are 1.35 years for the one-group model, and 1.26 years for the three-group model, both close to the data-derived value of 1.26 years. The full three-group model predicts an endemic prevalence of 23.8% while the collapsed one-group model

predicts an endemic prevalence of 24.5%. Thus the collapsed model estimate of endemic prevalence has an absolute error of less than 1% or a relative error of less than 3%.

For the South African epidemic, then, the one-group model reproduces the predictions of the three-group model well, though it slightly overestimates the steady-state prevalence. To explore the accuracy of the one-group model as an approximation to the three group model we varied the value of $\pi_m$, the protective efficacy for female-to-male transmission due to MC (Figure S5). Introducing a protective effect for females ($\pi_f > 0$) always decreases the difference between the models. For parameter ranges of interest to our investigation, output from the collapsed one-group model matches that of the full three-group model with reasonable accuracy (i.e. within a few percentage points). Because other uncertainties in the system far exceed this margin of error, we use the one-group model to fit the prevalence data and estimate the impact of MC.

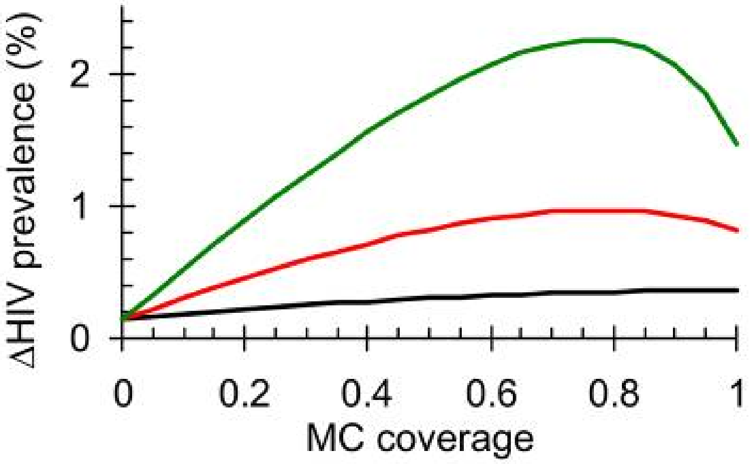


Figure S5. Absolute difference between the percent prevalence as predicted by the one-group (collapsed) model and the three-group model for different levels of MC coverage. The lines give different levels of protective efficacy $\pi_m$: green, 0.76; red, 0.60; black, 0.32. Other parameters are the same as in Figure S4.

## Decline of transmission with increasing prevalence

There is a distribution of sexual risk within all populations depending on gender, age and other social factors [13-16]. The EPP model [12] assumes that people are either at a fixed risk or zero risk and the model adjusts the size of the risk group to get the desired endemic prevalence. Here we make the more realistic assumption that risk varies continuously in the population. Since the functional form of the sexual risk distribution will, to some extent, determine the way in which incidence falls as prevalence rises, it will also determine the way in which the impact of MC varies with increasing efficacy. It must be the case that the average contact rate of uninfected people declines with prevalence. If this were not so then people at lower risk would have to be infected, on average, before those at higher risk.

Let us assume that there are some number of risk groups, each with characteristic contact rate $c_j$ ($j = 1, \ldots, n$), and that whenever a person dies in a certain risk group a new

person is recruited to the same risk group. This ensures that the proportion of people in each risk group remains constant over time whereas, in reality, the attrition of people in higher risk groups may reduce the overall risk of infection. This calculation will tend to provide a conservative estimate of the extent to which average risk among those who are still uninfected declines with prevalence. We denote the number of susceptible and infected individuals in group $j$ as $S_j$ and $I_j$, and the group size $N_j = S_j + I_j$. If individuals choose partners at random (i.e. the fraction of contacts with individuals in group $k$ is equal to the fraction of all contacts contributed by members of group $k$), then the dynamics of each risk group are [4,17]

$$\frac{dS_i}{dt} = \mu I_i - c_i \phi S_i \sum_{j=1}^{n} c_j \frac{I_j}{N} \tag{14}$$

so that at the steady state

$$\frac{I_i}{I_0} = \frac{c_i\left(N_i - I_i\right)}{c_0\left(N_0 - I_0\right)} \tag{15}$$

where we have chosen one of the groups, labelled 0, as the reference group. Let $i_j = I_j/N_j$ be the prevalence in group $j$, and $\Omega_j$ be the odds for the prevalence in group $j$, then

$$\frac{\Omega_i}{\Omega_0} = \frac{c_i}{c_0} \tag{16}$$

Table S1. The number of sexual partners in the last month as reported by men in Carletonville, South Africa [18]. ‘Other’ refers to people who say that they have had no sexual partners in the previous twelve months.

| No. partners | Frequency | No. partners | Frequency |
|---|---|---|---|
| 0 | 186 | 11 | 0 |
| 1 | 257 | 12 | 2 |
| 2 | 99 | 13 | 0 |
| 3 | 44 | 14 | 1 |
| 4 | 17 | 15 | 0 |
| 5 | 4 | 16 | 0 |
| 6 | 5 | 17 | 0 |
| 7 | 3 | 18 | 0 |
| 8 | 2 | 19 | 0 |
| 9 | 0 | 20 | 16 |
| 10 | 4 | Other | 1594 |

Q401: How many times have you had sexual intercourse with anyone in the last 12 months (other than a regular partner)? This includes mistresses, girlfriends, casual partners, prostitutes, or somebody you met in a bar or at a special occasion. (If none, then skip Q402).

Q402: How many different people have you had sexual intercourse with in the last month (apart from your regular partner?)

To obtain an admittedly rough estimate of the relative risk of infection at different prevalences we use data from a survey in Carletonville, South Africa, of the number of sexual partners (excluding regular partners) reported by men in the previous month [18]. We make several simplifying assumptions. First, we assume that the number of partners reported in the survey is indicative of each individual's habitual contact rate. Second, we assume that those who say that they have had a sexual partner in the last year but not in the last month are at the same risk as those who say that they have had one sexual partner in the last month (so that we combine the categories 0 and 1 in Table S1). Third we assume that those who say that they have not had a sexual partner in the last year are indeed at no risk of infection.

To determine the relationship between the prevalence and the average risk we first choose a value for the prevalence among those who have had one sexual partner in the last month, $i_0$. We then calculate the prevalence in all other classes, $i_j$, using Equation 15. Knowing the prevalence in each class we can calculate the overall prevalence

$$i = \frac{\sum_j i_j N_j}{\sum_j N_j} \qquad 17$$

We then calculate the transmission parameter for susceptible individuals in each group

$$\lambda_j = \phi c_j \frac{\sum_k c_k i_k N_k}{\sum_l c_l N_l} \qquad 18$$

To obtain the average value of the transmission parameter for all those who are still susceptible we take a weighted average over the groups

$$\lambda = \frac{\sum_j \lambda_j \left(1 - i_j\right) N_j}{\sum_k \left(1 - i_k\right) N_k} \qquad 19$$

Because the overall prevalence, $i$, is uniquely determined by the group prevalences, $i_j$, by Equations 17 to 19, the average force of infection can be expressed as a function of the overall prevalence, $\lambda(i)$. The normalized force of infection for a given prevalence is therefore $\lambda(i)/\lambda(0)$. We repeat the above process for a range of values of $i_0$ to generate a plot of this relationship (Figure S6).

Although the exponential curve is close to the curve based on the Carletonville data there are many assumptions and approximations involved in the estimation and the result should be treated as illustrative of the effect that variation in the risk of infection might have. However, the blue line must necessarily be conservative and in the absence of further data the exponential model seems reasonable.

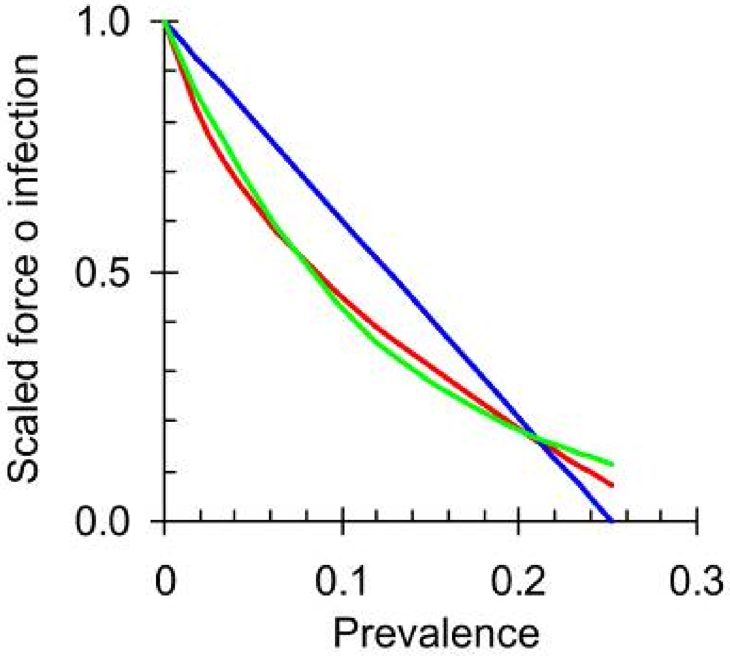


Figure S6. Force of infection, scaled to one at zero prevalence, as a function of prevalence. The red line is estimated from survey data for Carletonville, South Africa. The green line assumes that the risk declines exponentially with prevalence, the blue line that the risk has the same value for all those who are at risk as assumed in the EPP model. The curves are scaled to pass through the point where the curve based on the Carletonville data passes through the prevalence among men in the survey (21%).

## References

1. Anonymous (2004) Report on the Global AIDS Epidemic. Geneva: UNAIDS.
2. McLean AR, Blower SM (1995) Modelling HIV vaccination. Trends in Microbiology 3: 458-462.
3. Hyman JM, Li J (1997) Behavior Changes in SIS STD Models with Selective Mixing. SIAM Journal of Applied Mathematics 57: 1082-1094.
4. Garnett G (1998) The influence of behavioural heterogeneity on the population level effect of potential prophylactic type 1 human immunodeficiency virus vaccines. Journal of the Royal Statistical Society Series A 161: 209-225.
5. Blower S, Schwartz EJ, Mills J (2003) Forecasting the Future of HIV Epidemics: the Impact of Antiretroviral Therapies & Imperfect Vaccines. 5: 113-125.
6. Smith RJ, Blower SM (2004) Could disease-modifying HIV vaccines cause population-level perversity? Lancet Infect Dis 4: 636-639.

7. Williams BG, Gouws E, Colvin M, Sitas F, Ramjee G, et al. (2000) Patterns of infection: using age prevalence data to understand the epidemic of HIV in South Africa. South African Journal of Science 96: 305-312.

8. Lloyd-Smith JO, Getz WM, Westerhoff HV (2004) Frequency-dependent incidence in models of sexually transmitted diseases: portrayal of pair-based transmission and effects of illness on contact behaviour. Proc Biol Sci 271: 625-634.

9. CASCADE Collaboration (2000) Time from HIV-1 seroconversion to AIDS and death before widespread use of highly-active anti-retroviral therapy. A collaborative analysis. Lancet 355: 1131-1137.

10. Morgan D, Mahe C, Mayanja B, Okongo JM, Lubega R, et al. (2002) HIV-1 infection in rural Africa: is there a difference in median time to AIDS and survival compared with that in industrialized countries? AIDS 16: 597-603.

11. Anderson RM, May RM (1992) Infectious Diseases of Humans. Oxford.

12. Ghys PD, Brown T, Grassly NC, Garnett G, Stanecki KA, et al. (2004) The UNAIDS Estimation and Projection Package: a software package to estimate and project national HIV epidemics. Sexually Transmitted Infections 80: 5-9.

13. Carael M, Holmes K (2001) (editors) The multicentre study of factors determining the different prevalences of HIV in sub-Saharan Africa. AIDS 15: S1-S132.

14. Garnett GP, Anderson RM (1996) Sexually transmitted diseases and sexual behavior: insights from mathematical models. J Infect Dis 174 Suppl 2: S150-161.

15. Woolhouse ME, Dye C, Etard JF, Smith T, Charlwood JD, et al. (1997) Heterogeneities in the transmission of infectious agents: implications for the design of control programs. Proceedings of the National Academy of Sciences of the United States of America 94: 338-342.

16. Schneeberger A, Mercer CH, Gregson SA, Ferguson NM, Nyamukapa CA, et al. (2004) Scale-free networks and sexually transmitted diseases: a description of observed patterns of sexual contacts in Britain and Zimbabwe. Sexually Transmitted Diseases 31: 380-387.

17. Jacquez JA, Simon CP, Koopman J, Sattenspiel L, Perry T (1988) Modelling and Analysing HIV transmission: the effect of contact patterns. Mathematical Biosciences 92: 119-199.

18. Williams BG, Gilgen D, Campbell CM, Taljaard D, MacPhail C (2000) The Natural History of HIV/AIDS in South Africa: a biomedical and social survey. Johannesburg, South Africa: CSIR.
